# Three-Drug Regimens Containing Integrase Inhibitor Show Good Efficacy and Safety in Treatment-Naive Patients With HIV-1: A Bayesian Analysis

**DOI:** 10.3389/fphar.2021.603068

**Published:** 2021-07-21

**Authors:** Ke Zhang, Yang Zhang, Xinchao Liu, Aixin Li, Meixia Gao, Jianhua Hou, Chunxiang Guo, Tong Zhang, Hao Wu, Guanzhi Chen, Xiaojie Huang

**Affiliations:** ^1^ Department of Dermatology, The Affiliated Hospital of Qingdao University, Qingdao, China; ^2^ Center for Infectious Disease, Beijing Youan Hospital, Capital Medical University, Beijing, China; ^3^ Beijing Key Laboratory of HIV/AIDS Research, Beijing Youan Hospital, Capital Medical University, Beijing, China; ^4^ Department of Infectious Diseases, Peking Union Medical College Hospital, Beijing, China

**Keywords:** HIV, antiretroviral therapy, randomized controlled trials, integrase inhibitor, network meta-analysis

## Abstract

**Introduction:** The extensive utilisation of antiretroviral therapy has greatly improved the survival rates of those infected with human immunodeficiency virus (HIV). The objective of this study was to compare 3-drug regimens containing non-nucleoside reverse transcriptase inhibitor with 3-drug regimens containing integrase inhibitor (INI) regarding efficacy and safety in treatment-naive HIV-1-infected adults at 48 and 96 weeks, respectively.

**Methods:** This study was a network meta-analysis using a Bayesian methodology. On January 8, 2020, we searched databases and other sources for randomized controlled trials conducted in treatment-naive HIV-1 adults and compared multiple 3-drug antiretroviral regimens containing INI, efavirenz (EFV), or rilpivirine (RPV). We extracted data on the following outcomes: virologic suppression, CD4^+^ cell recovery, discontinuations, deaths, adverse events, serious adverse events, deaths related to study drugs, and drug-related adverse events. We conducted calculations within a Bayesian framework using R software.

**Results:** The network contained 15 randomized controlled trials including 9,745 patients. For efficacy outcomes, regimens containing INI, especially dolutegravir (DTG), were generally superior to other regimens. For virologic suppression at 48 weeks, odds ratios (95% credible intervals) were 0.6 (0.43, 0.82) for EFV+ tenofovir disoproxil fumarate (TDF)+emtricitabine (FTC) versus DTG+ abacavir+ lamivudine (3TC) and 0.52 (0.36, 0.75) for EFV+TDF+FTC vs. DTG+TDF+FTC/3TC. For safety outcomes, regimens containing INI tended to be safer relative to regimens without INI. Outcomes associated with death were unsuitable for network meta-analysis due to low event rates.

**Conclusion:** 3-drug regimens containing INI demonstrate better efficacy and safety than those containing RPV or EFV.

## Introduction

Those infected with human immunodeficiency virus (HIV) presently have a life expectancy similar to that of the general public due in large part to the extensive utilization of antiretroviral therapy (ART), which improves survival (Okulicz et al., 2013; Antiretroviral Therapy Cohort Collaboration, 2017; Gueler et al., 2017). Once HIV infection is confirmed, those infected are advised to receive ART as soon as possible. According to current guidelines, regimens for patients initiating ART usually consist of three antiretroviral (ARV) agents, two nucleoside reverse transcriptase inhibitors as a backbone and one core ARV drug chosen from boosted protease inhibitors with pharmacokinetic enhancers, integrase inhibitors (INIs), and non-nucleoside reverse transcriptase inhibitors [World Health Organization, 2016; Department of Health and Human Services, 2019; European AIDS Clinical Society (EACS), 2019]. INIs [bictegravir (BIC), dolutegravir (DTG), elvitegravir (EVG), and raltegravir (RAL)] are included as part of initial therapies for HIV type 1 (HIV-1) patients in most guidelines. 3-drug regimens containing non-nucleoside reverse transcriptase inhibitor such as rilpivirine (RPV) and efavirenz (EFV) are also used for many patients initiating therapy, especially in developing countries.

However, not all of these regimens have direct, head-to-head comparisons mainly because of the time- and money-consuming nature of randomized controlled trials (RCTs). For example, in phase 3 and phase 4 RCTs that were conducted in treatment-naive adults, BIC+tenofovir alafenamide (TAF)+emtricitabine (FTC) was compared directly only with regimens containing DTG (Gallant et al., 2017; Sax et al., 2017). Network meta-analysis (NMA) can simultaneously assess the relative efficacy and/or safety of more than two various interventions by combining direct and indirect evidence.

Additionally, those with HIV-1 often need to take ART for the rest of their lives, so the effectiveness (e.g., virologic suppression) and toxicity [e.g., adverse events (AEs)] of drugs are of concern. Many previous NMAs were of great significance in clinical practice, and the most commonly selected time point in those studies was 48 weeks (Patel et al., 2014; Gallien et al., 2018; Radford et al., 2019; Snedecor et al., 2019). This NMA compared triple-drug regimens containing INI with those containing RPV or EFV for their efficacy and safety at 48 and 96 weeks, respectively, in treatment-naive HIV-1 adults.

## Methods

We registered our protocol with OSF (https://osf.io/kb8s7) and conducted the current study on the basis of the PRISMA extension statement (Hutton et al., 2015).

### Study Identification and Selection Criteria

A systematic search of PubMed/MEDLINE, Embase, Web of Science, and the Cochrane Central Register of Controlled Trials for phase III/IV RCTs in treatment-naive HIV-1 adults was conducted on January 8, 2020. Relevant terms used and the full PubMed search strategy were provided in Supplementary Table S1. We also searched ClinicalTrials.gov (http://www.clinicaltrials.gov/) and scanned references of relevant systematic reviews and meta-analyses manually to ensure that no data potentially meeting the selection criteria were missing.

Eligible studies were phase 3 or phase 4 RCTs in treatment-naive HIV-1-infected adults. Eligible ARV regimens consisted of three standard dose ARV drugs of our interest, two nucleoside reverse transcriptase inhibitors (backbones) plus one core drug from RAL, EVG, DTG, BIC, EFV, and RPV. The backbones we were interested in were TAF+FTC, tenofovir disoproxil fumarate (TDF)+FTC/lamivudine (3TC), and abacavir (ABC)+3TC. We did not limit the use of pharmacokinetic enhancers such as cobicistat (c) in regimens. We also included 3-drug regimens containing low-dose EFV [EFV 400 mg (EFV400)] in the network. The studies that could be included should compare at least two regimens of interest and presented no less than one of the 48- or 96-weeks efficacy or safety outcomes mentioned later. The language of the publications was restricted in English. Articles that did not specify in a regimen which three drugs were used and studies in which the entire population was HIV-infected with tuberculosis were excluded.

### Outcomes

The following outcomes were frequently reported at multiple time points. Each of the outcomes was analyzed separately for the two time points: 48 and 96 weeks.

Patients with plasma HIV-1 RNA < 50 copies per mL were considered to have achieved virologic suppression. The proportion of subjects (intention-to-treat populations) with virologic suppression was the primary efficacy outcome. This outcome was also analyzed in two subgroups of subjects with viral loads (VLs) of ≤100,000 and >100,000 copies/mL at baseline. With reference to US Food and Drug Administration (FDA) guidance, there are several comparable algorithms for virologic suppression (US Department of Health and Human Services, 2015). If multiple algorithms were described in the same trial, we first selected FDA Snapshot 50, followed by time to loss of virologic response 50 and confirmed virologic response 50, and finally selected HIV RNA with less than 50 copies/mL. Cluster of differentiation 4 positive (CD4^+^) T cell recovery (the mean increase in CD4^+^ cell count from baseline) was the secondary efficacy outcome.

Safety outcomes analyzed were the proportions of subjects with death, death related to study drugs, discontinuations, AEs, drug-related AEs, and serious AEs.

### Data Extraction and Quality Evaluation

Titles/abstracts were independently screened by two investigators, and two investigators did full-text review and data extraction independently. After completing their work, two investigators doing the same work cross-checked the data they extracted. Discrepancies were first resolved by discussion and unresolvable problems relied on a third reviewer’s arbitration. We extracted the relevant data about the outcomes and characteristics of the trials and participants (Supplementary Tables S2–S4).

Cochrane’s risk of bias instrument was adopted to evaluate the quality of each included trial and we used Revman 5.3 software to produce relevant graphs (Higgins et al., 2011). We used the method introduced by Salanti et al. to rate the evidence quality, which was based on the Grading of Recommendations Assessment, Development, and Evaluation (GRADE) system (Salanti et al., 2014).

### Analysis

For each outcome, we conducted pairwise meta-analyses first if there were two or more studies comparing the same regimens. The *I* ^*2*^ statistic was used to identify the degree of heterogeneity, and *I* ^*2*^ of less than 50% was considered acceptable heterogeneity (Higgins et al., 2003). In cases when the data extracted were correct, if the heterogeneity was significant, we excluded the study that resulted in excessive heterogeneity.

We conducted the NMA within a Bayesian framework using R software (version 3.6.1) and the gemtc package (R Core Team, 2019; Brooks and Gelman, 1998), and R code we used can be found in Supplementary.

Bayesian analysis calculates the posterior probability that the research hypothesis is true by adding the information given in the likelihood (present data) to prior probability (previously known information) (Shim et al., 2019). It mainly has the following advantages: firstly, Bayesian analysis can make use of prior information (such as previous studies or empirical knowledge from related diseases), updated posterior information can be inferred by adding the prior information to the present data; secondly, it does not require large sample hypothesis and can reduce statistical errors caused by small sample size (Shim et al., 2019).

The results were calculated *via* Markov chain Monte Carlo methods and convergence was evaluated using the potential scale reduction factor (Valkenhoef and Kuiper, 2016). A potential scale reduction factor of less than 1.2 was acceptable (Valkenhoef and Kuiper, 2016). For binary outcomes (virologic suppression and safety outcomes), we used a binomial likelihood and the logit link function to build a logistic regression model. Continuous outcomes’ effects (CD4^+^ cell recovery) were modeled using an identity link and normal likelihood. We used the fixed-effects model unless the deviance information criterion value of the random-effects model was at least 3 less than that of the fixed-effects model. The consistency test was conducted using node-splitting analysis and we also compared the deviance information criterion values of the consistency and inconsistency models (Dias et al., 2010). The results were represented as odds ratios for binary outcomes or mean differences in continuous outcomes as well as corresponding 95% credible intervals. The probability of each therapeutic regimen at each ranking position was also calculated and we also calculated values of the surface under the cumulative ranking curve (SUCRA).

## Results

### Studies Included

**FIGURE 1:**
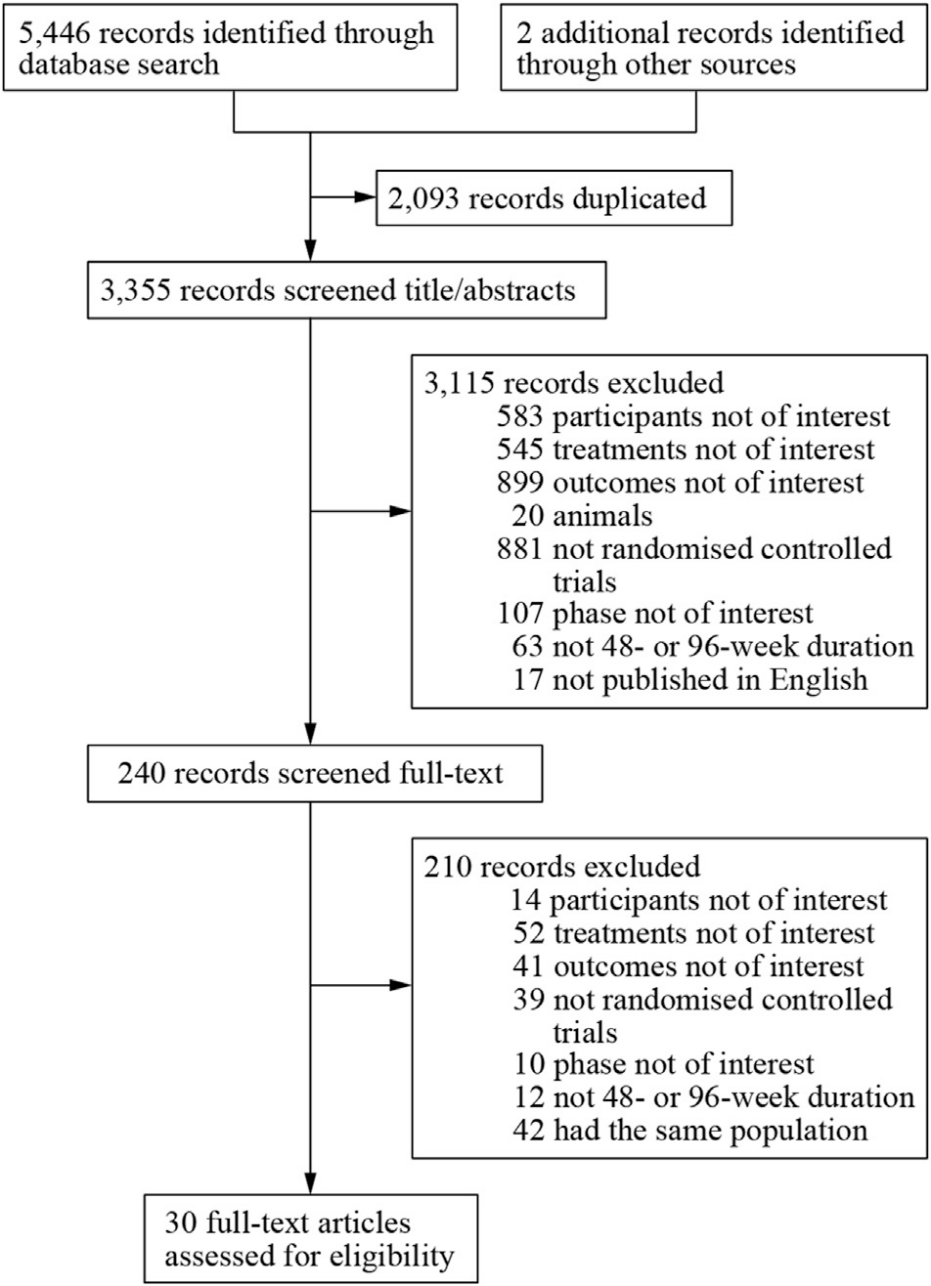
Flow chart of study selection.

**FIGURE 2:**
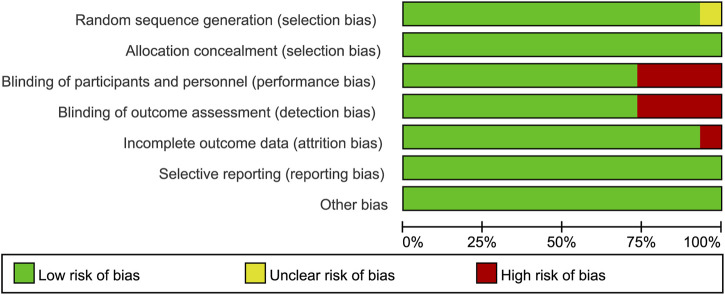
Risk of bias graph.

We obtained 5,448 citations *via* database searches and other sources, and 2,093 duplicates were excluded (Figure 1). We excluded 210 articles after screening full-text (Supplementary Table S5) and finally, 30 manuscripts pertaining to 15 RCTs including 9,745 subjects were in accordance with the inclusion criteria (Lennox et al., 2009; Lennox et al., 2010; Post et al., 2010; Cohen et al., 2011; Molina et al., 2011; Cohen et al., 2012; Sax et al., 2012; Cohen et al., 2013; Moyle et al., 2013; Nelson et al., 2013; Raffi et al., 2013a; Raffi et al., 2013b; Walmsley et al., 2013; Zolopa et al., 2013; Behrens et al., 2014; Clotet et al., 2014; Cohen et al., 2014; ENCORE1 Study Group, 2014; ENCORE1 Study Group, 2015; Molina et al., 2015; Sax et al., 2015; Walmsley et al., 2015; van Lunzen et al., 2016; Wohl et al., 2016; Gallant et al., 2017; Sax et al., 2017; Canadian Drug Expert Committee, 2018; Kouanfack et al., 2019; Stellbrink et al., 2019; Wohl et al., 2019). Most of the trials were phase three and the earliest of these trials began in 2000 while the latest began in 2016. Other information about each trial and the participants’ characteristics at baseline were demonstrated in Supplementary Table S2. Eleven of the 15 RCTs were rated low risk of bias and 4 RCTs [STaR (Cohen et al., 2014; van Lunzen et al., 2016), FLAMINGO (Clotet et al., 2014; Molina et al., 2015), ASSERT (Post et al., 2010; Moyle et al., 2013), ANRS12313 NAMSAL (The NAMSAL ANRS 12313 Study Group, 2019)] were rated high risk of bias (Figure 2 and Supplementary Figure S1). The fixed-effects model was used for all outcomes. Direct evidence and indirect evidence satisfied the condition of consistency in the whole analysis.

### Virologic Suppression

**FIGURE 3:**
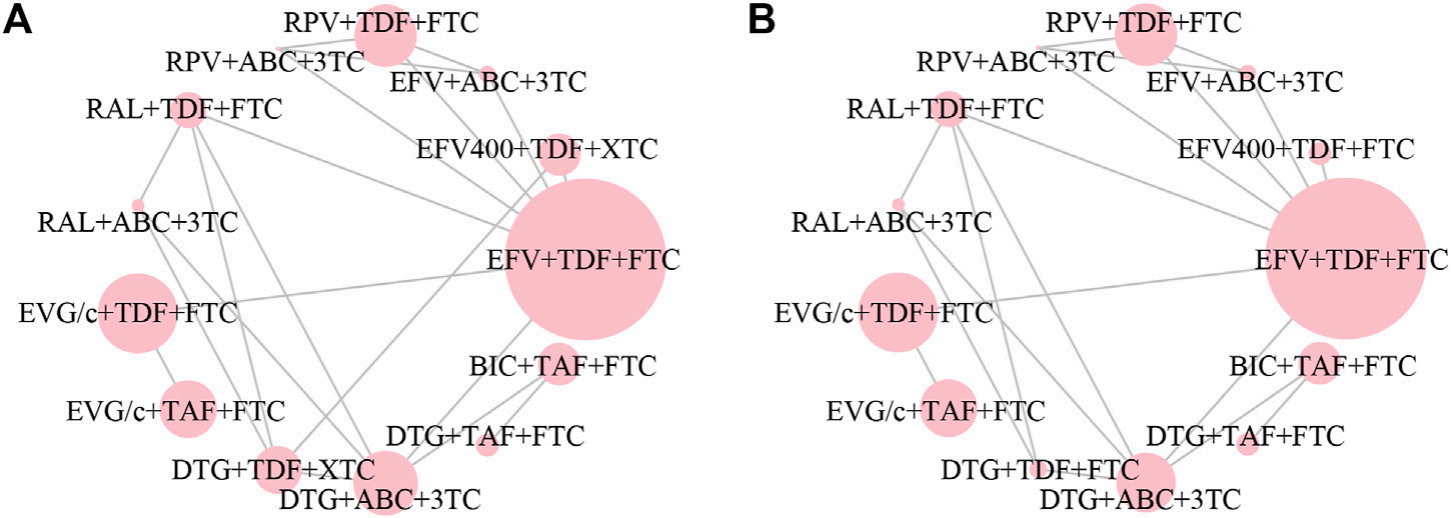
Network plot of treatment comparisons for the outcome of virologic suppression **(A)** at 48 weeks and **(B)** at 96 weeks. The larger the circle, the more participants included. ABC, abacavir; BIC, bictegravir; DTG, dolutegravir; EFV, efavirenz; EFV400, 400 mg efavirenz; EVG/c, cobicistat-boosted elvitegravir; FTC, emtricitabine; RAL, raltegravir; RPV, rilpivirine; TAF, tenofovir alafenamide; TDF, tenofovir disoproxil fumarate; XTC, FTC/3TC; 3TC, lamivudine.

All of the trials reported virologic suppression at 48 weeks. Fourteen trials reported it at 96 weeks but one was excluded from the analysis because of heterogeneity. The network of comparisons between the regimens was well connected, and EFV+TDF+FTC was the most well-connected regimen at 48 and 96 weeks (Figure 3). At 48 weeks, the estimated effects suggested that DTG+TAF+FTC had higher proportions of virologic suppression than the other regimens, although most comparisons had no statistical difference (Table 1). EFV+ABC+3TC was statistically inferior to the other ARV regimens with the exception of RPV+ABC+3TC at 48 weeks (Table 1). Judging from the 96-weeks estimated effects, except for the comparisons of RPV+TDF+FTC with some regimens containing EVG or BIC, the other comparisons showed that the proportions of virologic suppression in regimens containing INI were higher than standard dose regimens not containing INI. Additionally, DTG+TDF+FTC/3TC was statistically better than EFV+TDF+FTC and RPV+TDF+FTC at 48 and 96 weeks (Table 1). DTG+TAF+FTC was most likely to be the best regimen at 48 weeks (Table 2), but at 96 weeks it was replaced by DTG+TDF+FTC (Table 3).

**TABLE 1:** The relative efficacy of antiretroviral regimens for virologic suppression at 48 and 96 weeks.

EFV+ TDF+FTC	1.19 (0.8, 1.78)	0.77 (0.53, 1.11)	1.21 (0.97, 1.51)	0.58 (0.26, 1.45)	1.37 (0.98, 1.91)	1.38 (0.84, 2.29)	1.21 (0.81, 1.8)	1.35 (0.84, 2.18)	**2.64 (1.61, 4.39)**	**1.41 (1.06, 1.88)**	1.41 (0.68, 2.9)	1.16 (0.65, 2.06)
**0.71 (0.51, 0.99)**	EFV400+ TDF+XTC	0.65 (0.37, 1.12)	1.02 (0.65, 1.61)	0.49 (0.2, 1.32)	1.15 (0.68, 1.94)	1.16 (0.61, 2.21)	1.02 (0.58, 1.78)	1.14 (0.61, 2.12)	**2.22 (1.17, 4.24)**	1.19 (0.73, 1.94)	1.18 (0.51, 2.71)	0.98 (0.48, 1.97)
**1.47 (1.01, 2.14)**	**2.06 (1.24, 3.43)**	EFV+ ABC+3TC	**1.58 (1.03, 2.41)**	0.76 (0.31, 2)	**1.78 (1.08, 2.93)**	1.79 (0.97, 3.36)	1.57 (0.91, 2.71)	1.76 (0.96, 3.23)	**3.44 (1.85, 6.44)**	**1.84 (1.15, 2.94)**	1.83 (0.81, 4.12)	1.51 (0.76, 2.99)
0.85 (0.67, 1.08)	1.19 (0.79, 1.8)	**0.58 (0.37, 0.9)**	RPV+TDF+FTC	0.48 (0.21, 1.2)	1.13 (0.76, 1.68)	1.14 (0.66, 1.97)	1 (0.63, 1.57)	1.12 (0.66, 1.89)	**2.17 (1.26, 3.79)**	1.16 (0.81, 1.67)	1.16 (0.54, 2.47)	0.96 (0.51, 1.77)
0.57 (0.16, 1.55)	0.8 (0.22, 2.3)	0.39 (0.11, 1.11)	0.67 (0.19, 1.83)	RPV+ABC+3TC	2.34 (0.89, 5.66)	2.36 (0.84, 6.18)	2.07 (0.77, 5.13)	2.31 (0.83, 5.98)	**4.52 (1.61, 11.84)**	2.42 (0.93, 5.75)	2.4 (0.75, 7.19)	1.98 (0.67, 5.42)
0.73 (0.51, 1.04)	1.03 (0.67, 1.57)	**0.5 (0.3, 0.83)**	0.86 (0.56, 1.31)	1.28 (0.44, 4.75)	RAL+TDF+FTC	1.01 (0.65, 1.58)	0.88 (0.53, 1.49)	0.99 (0.55, 1.78)	**1.93 (1.24, 3.04)**	1.03 (0.73, 1.46)	1.03 (0.48, 2.18)	0.85 (0.46, 1.56)
0.62 (0.34, 1.07)	0.86 (0.47, 1.55)	**0.42 (0.21, 0.82)**	0.72 (0.38, 1.33)	1.08 (0.34, 4.28)	0.84 (0.48, 1.44)	RAL+ABC+3TC	0.88 (0.46, 1.65)	0.98 (0.49, 1.96)	**1.91 (1.16, 3.19)**	1.02 (0.64, 1.63)	1.02 (0.45, 2.3)	0.84 (0.42, 1.67)
0.74 (0.48, 1.14)	1.04 (0.6, 1.8)	**0.51 (0.29, 0.9)**	0.87 (0.53, 1.43)	1.3 (0.44, 4.94)	1.02 (0.58, 1.78)	1.21 (0.6, 2.49)	EVG/c+TDF+FTC	1.12 (0.86, 1.47)	**2.18 (1.16, 4.16)**	1.17 (0.72, 1.9)	1.16 (0.51, 2.66)	0.96 (0.47, 1.93)
0.58 (0.33, 1)	0.81 (0.43, 1.54)	**0.39 (0.2, 0.76)**	0.68 (0.37, 1.23)	1.02 (0.32, 4.01)	0.79 (0.41, 1.51)	0.94 (0.43, 2.09)	0.78 (0.55, 1.09)	EVG/c+TAF+FTC	1.95 (0.98, 3.91)	1.04 (0.6, 1.82)	1.04 (0.43, 2.48)	0.86 (0.4, 1.81)
**0.52 (0.36, 0.75)**	0.73 (0.54, 1)	**0.36 (0.21, 0.6)**	**0.61 (0.39, 0.95)**	0.91 (0.31, 3.4)	0.71 (0.48, 1.07)	0.85 (0.49, 1.5)	0.7 (0.4, 1.24)	0.9 (0.47, 1.75)	DTG+TDF+XTC	**0.54 (0.33, 0.85)**	0.53 (0.23, 1.2)	**0.44 (0.22, 0.87)**
**0.6 (0.43, 0.82)**	0.84 (0.56, 1.26)	**0.41 (0.25, 0.67)**	0.7 (0.47, 1.04)	1.05 (0.37, 3.85)	0.82 (0.55, 1.22)	0.97 (0.56, 1.73)	0.8 (0.47, 1.37)	1.03 (0.55, 1.95)	1.14 (0.77, 1.69)	DTG+ABC+3TC	1 (0.51, 1.94)	0.82 (0.49, 1.35)
0.42 (0.17, 1.01)	0.59 (0.24, 1.47)	**0.29 (0.11, 0.75)**	0.49 (0.2, 1.23)	0.74 (0.19, 3.39)	0.58 (0.23, 1.43)	0.68 (0.25, 1.86)	0.56 (0.21, 1.5)	0.73 (0.26, 2.05)	0.8 (0.32, 2)	0.7 (0.31, 1.6)	DTG+TAF+FTC	0.83 (0.53, 1.28)
0.66 (0.33, 1.3)	0.92 (0.45, 1.92)	**0.45 (0.21, 0.98)**	0.77 (0.37, 1.6)	1.16 (0.34, 4.81)	0.9 (0.44, 1.86)	1.07 (0.47, 2.46)	0.88 (0.39, 1.99)	1.14 (0.47, 2.73)	1.26 (0.61, 2.59)	1.1 (0.6, 2.02)	1.56 (0.9, 2.75)	BIC+TAF+FTC

Data are odds ratios (95% credible intervals) of the antiretroviral regimen column versus the antiretroviral regimen row (e.g., the effect of EFV+TDF+FTC versus EFV400+TDF+XTC was 0.71 with respect to virologic suppression at 48 weeks). Different antiretroviral regimens are on the diagonal. The values below the diagonal are the 48-weeks results and those above the diagonal are the 96-weeks results. Values in bold indicate statistically significant comparisons.

ABC, abacavir; BIC, bictegravir; DTG, dolutegravir; EFV, efavirenz; EFV400, 400 mg efavirenz; EVG/c, cobicistat-boosted elvitegravir; FTC, emtricitabine; RAL, raltegravir; RPV, rilpivirine; TAF, tenofovir alafenamide; TDF, tenofovir disoproxil fumarate; XTC, FTC/3TC; 3TC, lamivudine.

**TABLE 2:** Possibility of each treatment in each rank and the SUCRA value for each treatment for the virologic suppression outcome at 48 weeks.

Treatment	Rank 1	Rank 2	Rank 3	Rank 4	Rank 5	Rank 6	Rank 7	Rank 8	Rank 9	Rank 10	Rank 11	Rank 12	Rank 13	SUCRA
EFV+TDF+FTC	0.00	0.00	0.00	0.00	0.00	0.01	0.07	0.45	2.42	9.98	29.92	55.76	1.39	0.13
EFV400+TDF+XTC	0.11	0.98	3.06	6.05	9.58	13.20	16.33	17.23	15.35	11.11	5.48	1.43	0.09	0.46
EFV+ABC+3TC	0.00	0.00	0.00	0.00	0.00	0.01	0.03	0.07	0.16	0.46	1.39	6.91	90.98	0.01
RPV+TDF+FTC	0.01	0.06	0.26	0.70	1.59	3.37	6.43	11.28	18.14	25.21	26.31	6.36	0.27	0.29
RPV+ABC+3TC	25.11	12.72	7.48	5.74	5.11	4.92	4.86	4.99	5.09	5.61	5.08	9.44	3.85	0.63
RAL+TDF+FTC	0.17	0.86	2.33	4.69	7.79	11.87	15.58	17.46	16.77	12.97	7.02	2.32	0.18	0.44
RAL+ABC+3TC	6.15	10.81	11.86	11.53	11.08	10.54	9.63	8.55	7.19	5.67	3.85	2.76	0.39	0.61
EVG/c+TDF+FTC	0.10	1.61	3.94	5.89	7.58	9.32	11.58	13.49	15.06	14.54	10.47	5.79	0.63	0.42
EVG/c+TAF+FTC	10.28	15.28	13.95	11.58	10.31	9.67	8.78	7.60	5.97	3.64	1.87	0.94	0.13	0.68
DTG+TDF+XTC	10.41	21.85	23.55	18.23	12.27	7.39	3.85	1.67	0.58	0.17	0.04	0.00	0.00	0.79
DTG+ABC+3TC	1.15	5.73	13.60	20.09	20.99	17.13	11.22	6.15	2.79	0.95	0.21	0.01	0.00	0.66
DTG+TAF+FTC	45.88	18.97	8.90	5.92	4.46	3.59	2.97	2.61	2.20	1.76	1.55	0.94	0.25	0.84
BIC+TAF+FTC	0.63	11.14	11.05	9.58	9.24	8.98	8.68	8.47	8.29	7.91	6.82	7.35	1.84	0.53

Units of values except SUCRA values: %

ABC, abacavir; BIC, bictegravir; DTG, dolutegravir; EFV, efavirenz; EFV400, 400 mg efavirenz; EVG/c, cobicistat-boosted elvitegravir; FTC, emtricitabine; RAL, raltegravir; RPV, rilpivirine; SUCRA, the surface under the cumulative ranking curve; TAF, tenofovir alafenamide; TDF, tenofovir disoproxil fumarate; 3TC, lamivudine.

**TABLE 3:** Possibility of each treatment in each rank and the SUCRA value for each treatment for the virologic suppression outcome at 96 weeks.

Treatment	Rank 1	Rank 2	Rank 3	Rank 4	Rank 5	Rank 6	Rank 7	Rank 8	Rank 9	Rank 10	Rank 11	Rank 12	Rank 13	SUCRA
EFV+TDF+FTC	0.00	0.00	0.00	0.02	0.11	0.67	2.69	8.19	18.84	27.73	34.65	6.71	0.38	0.25
EFV400+TDF+FTC	0.43	5.93	6.57	7.41	8.42	9.57	11.02	11.98	11.79	11.39	10.17	4.48	0.84	0.48
EFV+ABC+3TC	0.00	0.02	0.05	0.11	0.21	0.40	0.75	1.40	2.57	5.93	10.47	54.29	23.80	0.10
RPV+TDF+FTC	0.07	2.25	4.51	7.36	10.70	13.94	17.00	16.98	14.51	9.61	2.61	0.44	0.03	0.50
RPV+ABC+3TC	0.23	1.02	0.74	0.72	0.82	0.96	1.24	1.57	1.97	2.88	4.27	14.08	69.51	0.08
RAL+TDF+FTC	0.05	9.38	14.56	15.53	15.54	14.25	11.60	8.71	5.62	3.07	1.30	0.35	0.04	0.63
RAL+ABC+3TC	0.46	18.60	13.63	11.79	10.68	9.76	8.74	7.78	6.54	5.21	4.39	2.00	0.41	0.63
EVG/c+TDF+FTC	0.12	2.19	8.42	8.67	9.27	10.63	12.21	13.40	12.72	10.66	7.99	3.16	0.56	0.48
EVG/c+TAF+FTC	2.34	17.62	12.97	10.59	9.92	9.64	9.81	8.79	7.05	5.27	3.92	1.72	0.36	0.63
DTG+TDF+FTC	90.11	7.54	1.64	0.44	0.15	0.07	0.03	0.01	0.00	0.00	0.00	0.00	0.00	0.99
DTG+ABC+3TC	0.12	9.00	17.10	21.69	19.24	14.65	9.49	5.40	2.36	0.77	0.17	0.03	0.00	0.68
DTG+TAF+FTC	5.93	24.01	10.90	8.21	7.24	6.75	6.45	6.25	5.98	6.51	6.35	4.00	1.42	0.63
BIC+TAF+FTC	0.14	2.44	8.91	7.46	7.71	8.71	8.98	9.54	10.05	10.96	13.71	8.76	2.65	0.43

Units of values except SUCRA values: %

ABC, abacavir; BIC, bictegravir; DTG, dolutegravir; EFV, efavirenz; EFV400, 400 mg efavirenz; EVG/c, cobicistat-boosted elvitegravir; FTC, emtricitabine; RAL, raltegravir; RPV, rilpivirine; SUCRA, the surface under the cumulative ranking curve; TAF, tenofovir alafenamide; TDF, tenofovir disoproxil fumarate; 3TC, lamivudine.

### CD4^+^ Cell Recovery

A total of 7,895 participants were analyzed at 48 weeks and 7,324 subjects were assessed at 96 weeks. Regimens analyzed at the two time points were not exactly the same (Supplementary Figure S2). The CD4^+^ cell count of ARV regimens containing INI resulted in a greater increase than EFV400+TDF+FTC/3TC, EFV+TDF+FTC, and RPV+TDF+FTC from baseline to 48 weeks. EFV+TDF+FTC showed a mean difference in the CD4^+^ cell count of -83.04 cells/μL (95% credible intervals, -131.14, -35.19) compared with DTG+TAF+FTC at 48 weeks (Supplementary Table S6). At 96 weeks, most of the regimens were not statistically different from one another, except a few such as EFV+TDF+FTC and regimens containing DTG. Mean difference (95% credible interval) of BIC+TAF+FTC vs. DTG+TAF+FTC was -43.8 (-78.33, -9.47) showing that DTG+TAF+FTC was superior to BIC+TAF+FTC with respect to CD4^+^ cell recovery at 96 weeks (Supplementary Table S6). At these two time points, the best regimen might be DTG+TAF+FTC and the worst might be EFV+TDF+FTC (Supplementary Tables S7, S8).

### Discontinuations

Overall, 12 trials consisting of 8,207 participants reported discontinuations at 48 weeks. At 96 weeks, 12 trials consisting of 8,000 participants reported discontinuations. EFV+TDF+FTC was still the most well-connected regimen at 48 and 96 weeks (Supplementary Figure S3). The 48-weeks results showed that DTG+ABC+3TC and EVG/c+TDF+FTC had less AEs than regimens containing EFV (Supplementary Table S9). At 96 weeks, most of the statistically significant estimated effects were similar to the 48-weeks results. Except for a few results from the comparisons between regimens containing EFV400 or RPV with regimens containing INI, the results of comparisons between other regimens not containing INI with regimens containing INI showed that the latter had lower proportions of discontinuations at 96 weeks (Supplementary Table S9). DTG+TDF+3TC and DTG+TAF+FTC was the safest ARV regimen at 48 and 96 weeks, respectively (Supplementary Tables S10, S11).

### Deaths and Deaths Related to Study Drugs

At 48 weeks, there were 36 deaths in 12 trials. However, in the 36 deaths, many treatment groups reported no deaths, making the network meta-analysis unreliable. At 96 weeks, a total of 32 deaths were reported in 9 trials. The situation was similar to that at 48 weeks.

For deaths related to study dugs, 16 of the 36 deaths at 48 weeks and 17 of the 32 deaths at 96 weeks did not report whether the cause of death was due to study drugs. Only one death was associated with the study drug at the two time points. In these situations, network meta-analyses were also not suitable.

### Adverse Events and Drug-Related Adverse Events

Only seven regimens from seven trials formed the evidence network for AEs at 48 weeks, and eight comparison regimens from six trials could not be connected to a stable evidence network at 96 weeks (Supplementary Figure S4). At 48 weeks, RAL+TDF+FTC was the safest regimen, followed by BIC+TAF+FTC compared with regimens without INI (Supplementary Table S12). Odds ratio of BIC+TAF+FTC vs. DTG+TAF+FTC was 1.09 showing that the proportion of AEs in DTG+TAF+FTC was higher. DTG+ABC+3TC had a higher risk of AEs than BIC+TAF+FTC at 48 weeks (Supplementary Table S12). At 96 weeks, compared with regimens not containing INI, RAL+TDF+FTC had less AEs, although the results were not statistically significant (Supplementary Table S12). Possibility of each treatment in each rank and the SUCRA values provided some references for the sorting of several regimens for comparison (Supplementary Tables S13, S14).

For drug-related AEs, seven and six treatment regimens were compared at 48 and 96 weeks, respectively (Supplementary Figure S5). At these two time points, the results of comparisons between regimens without INI and regimens with INI indicated that the former had a higher risk of drug-related AEs. And the results of BIC+TAF+FTC vs. DTG+TAF+FTC showed that the former was safer than the latter (Supplementary Table S15). In these compared regimens, BIC+TAF+FTC was the most likely regimen to have the lowest risk of drug-related AEs at the two timepoints (Supplementary Tables S16, S17).

### Serious Adverse Events

For serious AEs, the regimens compared at the two time points were not totally the same (Supplementary Figure S6). There was no statistically significant comparisons compared treatments at 48 weeks (Supplementary Table S18). At 96 weeks, DTG+TAF+FTC showed better safety, it was not only superior to regimens containing EFV, but also to DTG+ABC+3TC and BIC+TAF+FTC (Supplementary Table S18). RPV+TDF+FTC and DTG+TAF+FTC had the highest probability to have the lowest risk of serious AEs at 48 and 96 weeks, respectively (Supplementary Tables S19, S20).

### Subgroup Analysis

Subjects were divided into two subgroups based on whether their VLs were greater than 100,000 copies/mL at baseline. The difference between the treatment regimens compared at the two time points showed in Supplementary Figures S7, S8. For the 96-weeks low VL group, we excluded one trial because of heterogeneity, so the treatment regimens compared at the two groups were different (Supplementary Figure S8). In the 48-weeks low VL group, EFV+ABC+3TC was statistically inferior to almost all of the other ARV regimens, similar to the 48-weeks results for all of the subjects. But unlike the 48-weeks estimated effects of the primary outcome, EFV400+TDF+FTC/3TC had lower proportions of virologic suppression than all of the regimens containing INI. In the 48-weeks group of subjects with VLs more than 100,000 copies/mL at baseline, most of the ARV regimens were not statistically different from the others. In this group, EFV+ABC+3TC were inferior to DTG+TDF+FTC/3TC and DTC+ABC+3TC (Supplementary Table S21). The results of the 96-weeks low VL group also showed that regimens without INI were generally inferior to regimens with INI, but the results were slightly different in another group (Supplementary Table S22). In two 48-weeks subgroups and a 96-weeks high VL group, DTG+TAF+FTC had the greatest potential to be the most desirable regimen, while in the remaining group, that regimen was DTG+ABC+3TC (Supplementary Tables S23–S26).

## Discussion

This NMA was designed to compare multiple 3-drug ARV regimens containing INI, EFV, and RPV in terms of efficacy and safety as initial treatments for HIV-1-infected adults, providing comparative evidence of regimens with no direct, head-to-head RCTs. Our study found that regimens containing INI generally had better efficacy and safety than regimens not containing INI. By comparing the probabilities of each treatment in each ranking position in our analysis, the evidence suggested that EFV+ABC+3TC was the least desirable regimen in almost every aspect. In regimens containing INI, DTG+TAF+FTC and DTG+TDF+FTC/3TC had advantages in efficacy, and BIC+TAF+FTC had a better safety performance.

Previous NMAs comparing core antivirals demonstrated that DTG was significantly better than EFV in terms of virologic suppression at 48 weeks (Patel et al., 2014; Kanters et al., 2016; Snedecor et al., 2019). But the network nodes of our analysis were defined by specific ARV regimens, not specific antivirals. For virologic suppression at 48 weeks, the results of the current NMA were basically consistent with previous studies comparing core antivirals. Except that the comparison between DTG+TAF+FTC and EFV+TDF+FTC was not statistically significant, regimens containing DTG were superior to regimens containing EFV. Both specific antivirals and specific ARV regimens have certain advantages as network nodes for research. When we know that DTG has a better efficacy, comparisons between regimens could provide some reference for choosing a better regimen with DTG. A previous NMA comparing the efficacy and safety of EVG/c+TAF+FTC with other regimens at 48 weeks demonstrated that EFV+ABC+3TC was inferior to EVG/c+TAF+FTC in terms of virologic suppression, and the current NMA also confirmed it (Patel et al., 2014). In addition, unlike previous NMAs that compared treatments only at 48 weeks (Patel et al., 2014; Gallien et al., 2018; Radford et al., 2019; Snedecor et al., 2019), the present study also synthesized data from 96-weeks studies, and three-drug regimens containing INI showed good efficacy and safety at 96 weeks.

Acquired immune deficiency syndrome has become a chronic disease and those living with the virus require lifelong ART. Therefore, reducing drug costs and toxicity warrant investigation. Dose reduction and using 2-drug regimens are the main methods at present. Both trials, ANRS 12313 NAMSAL and ENCORE1, compared the efficacy and safety of EFV400+TDF+FTC/3TC with another regimen (ENCORE1 Study Group, 2014; ENCORE1 Study Group, 2015; The NAMSAL ANRS 12313 Study Group, 2019). Regarding virologic suppression at 48 weeks, the results of the current NMA were basically consistent with the results of ANRS 12313 NAMSAL that DTG+TDF+3TC was non-inferior to EFV400+TDF+3TC. Besides, the evidence in our study suggested that regimens containing DTG had advantages over EFV400+TDF+FTC/3TC in some aspects, such as changes in CD4^+^ cell count and drug-related AEs. The current NMA confirmed that the regimen containing EFV400 was superior to EFV+TDF+FTC in terms of drug-related AEs and 48-weeks virologic suppression, which was partly different from ENCORE1. ENCORE1 concluded that EFV400 was non-inferior to standard-dose EFV when combined with TDF and FTC in terms of 48-weeks virologic suppression.

In our analysis, four trials were rated high risk mostly because these trials were open-label. For each estimated result, we rated it as one of the four grades and most results were rated “high” or “moderate” (Supplementary Tables S27–S34). Study limitations and imprecision were two of the most frequent reasons for downgrading. More double-blind RCTs are needed in the future to assess the clinical efficacy and safety of ARV regimens.

Our study has some limitations. First, we only compared the efficacy and safety of these ARV regimens at 48 and 96 weeks, but do not know how these ARV regimens perform in various outcomes after a longer period of time. Second, not all of the included regimens could be analyzed in each outcome, mainly because of limited data. This deficiency was particularly evident in the outcomes associated with AEs. Moreover, in addition to efficacy and safety, drug resistance is an important area of concern for drugs, especially as people with HIV often require lifelong treatment. Unfortunately, our study failed to investigate drug resistance among different treatment regimens, and we will investigate it further in the future. In addition, baseline characteristics of the included population in each study we included were not exactly the same. Although heterogeneity analysis and consistency analysis were conducted to minimize the bias, it was still impossible to eliminate all the bias. Moreover, increasing numbers of studies have compared the efficacy and safety of 2-drug regimens (Capetti et al., 2018; Llibre et al., 2018; Cahn et al., 2019; Ciccullo et al., 2019), and DTG+3TC has become a newer regimen recommended as a first-line treatment (European AIDS Clinical Society (EACS), 2019). A previous NMA showed that DTG+3TC had similar efficacy and safety to traditional 3-drug regimens containing INI (Radford et al., 2019). Using two-drug regimens may be a new trend, but unfortunately, we have limited the number of drugs and not analyze the two-drug regimen. Nevertheless, despite these limitations, our NMA can still provide some reference value for HIV-1 patients selecting ARV regimens.

In conclusion, we found that 3-drug regimens containing INI show better efficacy and safety than 3-drug regimens containing RPV or EFV for treatment-naive HIV-1 adults.

## Data Availability

The original contributions presented in the study are included in the article/Supplementary Material, further inquiries can be directed to the corresponding authors.

## Abbreviations

HIV, Human immunodeficiency virus; ART, Antiretroviral therapy; ARV, antiretroviral; INI, Integrase inhibitor; BIC, Bictegravir; DTG, Dolutegravir; EVG, Elvitegravir; RAL, Raltegravir; HIV-1, HIV type 1; RPV, Rilpivirine; EFV, Efavirenz; RCTs, Randomized controlled trials; TAF, Tenofovir alafenamide; FTC, Emtricitabine; NMA, Network meta-analysis; AEs, Adverse events; TDF, Tenofovir disoproxil fumarate; 3TC, Lamivudine; ABC, Abacavir; EFV400, EFV 400 mg; VLs, Viral loads; FDA, Food and Drug Administration; CD4^+^, Cluster of differentiation 4 positive; SUCRA, the surface under the cumulative ranking curve.
