# Supplementary material for: Three-Drug Regimens Containing Integrase Inhibitor Show Good Efficacy and Safety in Treatment-Naive Patients With HIV-1: A Bayesian Analysis
Source: Front Pharmacol. 2021 Jul 21;12:603068. doi: 10.3389/fphar.2021.603068 (PMC8334007; doi:10.3389/fphar.2021.603068)
Supplement: Supplementary file 1 [file DataSheet1.docx]

Supplementary Material

**Supplementary Table S1.** PubMed search strategy

| **Term group** | **Query** | **Search terms** | **Tags** |
| --- | --- | --- | --- |
| HIV/AIDS terms | #1 | HIV Infection | MESH |
|  | #2 | HIV | MESH |
|  | #3 | HIV | TIAB |
|  | #4 | Human Immunodeficiency Virus | TIAB |
|  | #5 | Human Immunedeficiency Virus | TIAB |
|  | #6 | Human Immuno-deficiency Virus | TIAB |
|  | #7 | Human Immune-deficiency Virus | TIAB |
|  | #8 | Acquired Immunodeficiency Syndrome | TIAB |
|  | #9 | Acquired Immunedeficiency Syndrome | TIAB |
|  | #10 | Acquired Immuno deficiency Syndrome | TIAB |
|  | #11 | Acquired Immune deficiency Syndrome | TIAB |
|  | #12 | AIDS | TIAB |
|  | #13 | #1 OR #2 OR #3 OR #4 OR #5 OR #6 OR #7 OR #8 OR #9 OR #10 OR #11 OR #12 | |
| Treatment failure and experienced | #14 | Salvage therapy | TIAB |
|  | #15 | Treatment Failure | MESH |
|  | #16 | Treatment-experienced | TIAB |
|  | #17 | Antiretroviral experienced | TIAB |
|  | #18 | ART-experienced | TIAB |
|  | #19 | Experienced patients | TIAB |
|  | #20 | treatment switch* | TIAB |
|  | #21 | #14 OR #15 OR #16 OR #17 OR #18 OR #19 OR #20 | |
| Adults | #22 | Adult* | All Fields |
|  | #23 | Adult | MESH |
|  | #24 | #22 OR #23 | |
| **Population final** | #25 | #13 AND #24 NOT #21 | |
| Intervention and comparators | #26 | "Non-nucleoside reverse transcriptase inhibitor" | TIAB |
|  | #27 | "Non-nucleoside reverse transcriptase inhibitors" | TIAB |
|  | #28 | NNRTI | TIAB |
|  | #29 | Rilpivirine OR RPV OR Edurant OR Compzlera OR Eviplera OR Odefsey OR TMC278 | TIAB |
|  | #30 | Efavirenz OR EFV OR Stocrin OR Sustiva OR Atripla OR DMP-266 | TIAB |
|  | #31 | "Integrase Inhibitor" | TIAB |
|  | #32 | "Integrase inhibitors" | TIAB |
|  | #33 | INSTI | TIAB |
|  | #34 | Dolutegravir OR DTG OR Tivicay OR Triumeq OR GSK1349572 | TIAB |
|  | #35 | Raltegravir OR RAL OR Isentress OR MK-0518 | TIAB |
|  | #36 | Elvitegravir OR EVG OR Vitekta OR Genvoya OR Stribild OR Quad OR GS-9137 OR JTK-303 | TIAB |
|  | #37 | Bictegravir OR BIC OR Biktarvy OR GS-9883 | TIAB |
| **Intervention and comparators final** | #38 | #26 OR #27 OR #28 OR #29 OR #30 OR #31 OR #32 OR #33 OR #34 OR #35 OR #36 OR #37 | |
| Randomized controlled trial terms | #39 | randomized controlled trial | Publication Type |
|  | #40 | controlled clinical trial | Publication Type |
|  | #41 | randomized | TIAB |
|  | #42 | placebo | TIAB |
|  | #43 | clinical trials as topic | MESH,NoExp |
|  | #44 | randomly | TIAB |
|  | #45 | trial | TI |
|  | #46 | #39 OR #40 OR #41 OR #42 OR #43 OR #44 OR #45 | |
|  | #47 | animals | MESH |
|  | #48 | humans | MESH |
|  | #49 | #47 NOT #48 | |
| **Study design final** | #50 | #46 NOT #49 | |
| **Complete search** | #51 | #25 AND #38 AND #50 | |

**Supplementary Table S2.** Characteristics of the trials and participants

| **Study name** | **Treatments** | **ITT popu-lation** | **Year of initiation** | **Trial phase** | **Setting** | **Median age, years** | **% Male** | **Median baseline VL, log_10_ RNA copies/mL** | **Median baseline CD4^+^, cells/mL** |
| --- | --- | --- | --- | --- | --- | --- | --- | --- | --- |
|  |  |  |  |  |  |  |  |  |  |
| ANRS 12313 NAMSAL | DTG+TDF+3TC | 613 | 2016 | 3 | Cameroon | 38 | 36.5 | 5.3 | 289 |
|  | EFV400+TDF+3TC |  |  |  |  | 36 | 31.7 | 5.3 | 271 |
| ASSERT | EFV+ABC+3TC | 385 | 2007 | 4 | Europe | 38 | 83 | 5.01 | 240 |
|  | EFV+TDF+FTC |  |  |  |  | 36 | 80 | 5.12 | 230 |
| ECHO | RPV+TDF+FTC | 690 | 2008 | 3 | USA, Canada, Australia, South Africa, Europe, Asia, and Latin America | 36 | 77 | 5 | 240 |
|  | EFV+TDF+FTC |  |  |  |  | 36 | 80 | 5 | 257 |
| ENCORE1 | EFV400+TDF+FTC | 630 | 2011 | 4 | Argentina, Australia, Chile, Germany, Hong Kong, Israel, Malaysia, Mexico, Nigeria, Singapore, South Africa, Thailand, and the UK | 36.1^a^ | 68.8 | 4.76^a^ | 273^a^ |
|  | EFV+TDF+FTC |  |  |  |  | 35.8 ^a^ | 66.5 | 4.73 ^a^ | 272 ^a^ |
| FLAMINGO | DTG+ABC+3TC | 484^c^ | 2011 | 3B | France, Germany, Italy, Puerto Rico, Romania, Russia, Spain, Switzerland, and the USA | 34 | 87 | 4.49 | 390 |
|  | DTG+TDF+FTC |  |  |  |  |  |  |  |  |
|  | DRV/r+ABC+3TC ^b^ |  |  |  |  | 34 | 83 | 4.48 | 400 |
|  | DRV/r+TDF+FTC ^b^ |  |  |  |  |  |  |  |  |
| GS-US-236-0102 | EVG/c+TDF+FTC | 700 | 2010 | 3 | North America | 37 | 88 | 4.75 | 376 |
|  | EFV+TDF+FTC |  |  |  |  | 38 | 90 | 4.78 | 383 |
| GS-US-292-0104/0111 | EVG/c+TAF+FTC | 1,733 | 2013 | 3 | North America, Europe, Australia, Japan, and Thailand/North America, Europe, and Latin America | 33 | 85 | 4.58 | 404 |
|  | EVG/c+TDF+FTC |  |  |  |  | 35 | 85 | 4.58 | 406 |
| GS-US-380-1489 | BIC+TAF+FTC | 629 | 2015 | 3 | Europe (Belgium, France, Germany, Italy, Spain, and the UK), Latin America (Dominican Republic), and North America (Canada and the USA) | 31 | 91 | 4.42 | 443 |
|  | DTG+ABC+3TC |  |  |  |  | 32 | 90 | 4.51 | 450 |
| GS-US-380–1490 | BIC+TAF+FTC | 645 | 2015 | 3 | Australia, Belgium, France, Germany, Italy, Spain, the UK, Dominican Republic, the USA, and Canada | 33 | 88 | 4.43 | 440 |
|  | DTG+TAF+FTC |  |  |  |  | 34 | 89 | 4.45 | 441 |
| SINGLE | DTG+ABC+3TC | 833 | 2011 | 3 | North America, Europe, and Australia | 36 | 84 | 4.67 | 334.5 |
|  | EFV+TDF+FTC |  |  |  |  | 35 | 85 | 4.70 | 339.0 |
| SPRING-2 | DTG+ABC+3TC | 822 | 2010 | 3 | Canada, USA, Australia, and Europe | 37 | 85 | 4.52 | 359 |
|  | DTG+TDF+FTC |  |  |  |  |  |  |  |  |
|  | RAL+ABC+3TC |  |  |  |  | 35 | 86 | 4.58 | 362 |
|  | RAL+TDF+FTC |  |  |  |  |  |  |  |  |
| STaR | RPV+TDF+FTC | 786 | 2011 | 3B | Australia, Austria, Belgium, Canada, France, Germany, Portugal, Puerto Rico, Spain, Switzerland, the United Kingdom, and the United States | 37 | 93 | 4.8 ^a^ | 396 ^a^ |
|  | EFV+TDF+FTC |  |  |  |  | 35 | 93 | 4.8 ^a^ | 385 ^a^ |
| STARTMRK | RAL+TDF+FTC | 563 | 2006 | 3 | Australia, Brazil, Canada, Chile, Colombia, France, Germany, India, Italy, Mexico, Peru, Spain, Thailand, and USA | 37 | 81 | 5.1 | 212 |
|  | EFV+TDF+FTC |  |  |  |  | 36 | 82 | 5.0 | 204 |
| THRIVE | RPV+TDF+FTC | 678^c^ | 2008 | 3 | US and Puerto Rico, Canada, Australia, Europe, South Africa, Asia, and Latin America | 36 | 74 | 5 | 263 |
|  | RPV+AZT+3TC ^b^ |  |  |  |  |  |  |  |  |
|  | RPV+ABC+3TC |  |  |  |  |  |  |  |  |
|  | EFV+TDF+FTC |  |  |  |  | 36 | 72 | 5 | 263 |
|  | EFV+AZT+3TC ^b^ |  |  |  |  |  |  |  |  |
|  | EFV+ABC+3TC |  |  |  |  |  |  |  |  |

^a^ Mean.

^b^ Treatment regimen that did not meet the inclusion criteria.

^c^ Not all of these people were included in the analysis.

ABC, abacavir; BIC, bictegravir; DTG, dolutegravir; EFV, efavirenz; EFV400, 400mg efavirenz; EVG/c, cobicistat-boosted elvitegravir; FTC, emtricitabine; RAL, raltegravir; RPV, rilpivirine; TAF, tenofovir alafenamide; TDF, tenofovir disoproxil fumarate; VL, viral load; VS, virologic suppression; 3TC, lamivudine.

**Supplementary Table S3.** Data extracted from the included studies

| **Trial** | **Treatment** | **N** | **VS HIV RNA  <50 copies/mL, n/N** | |  | **48-week subgroup analysis, n/N** | |  | **96-week subgroup analysis, n/N** | |  | **Discontinuation, n/N** | |  | **CD4^+^ change, mean (SD)/n** | |
| --- | --- | --- | --- | --- | --- | --- | --- | --- | --- | --- | --- | --- | --- | --- | --- | --- |
|  |  |  | **48-week** | **96-week** |  | **≤100,000** | **>100,000** |  | **≤100,000** | **>100,000** |  | **48-week** | **96-week** |  | **48-week** | **96-week** |
| ANRS 12313 NAMSAL | DTG+TDF+3TC | 310 | 231/310 | ‒ |  | 94/103 | 137/207 |  | ‒ | ‒ |  | 15 | ‒ |  | 185.71 (128.14)/292 | ‒ |
|  | EFV400+TDF+3TC | 303 | 209/303 | ‒ |  | 86/103 | 123/200 |  | ‒ | ‒ |  | 22 | ‒ |  | 155.61 (120.72)/ 279 | ‒ |
| ASSERT | EFV+ABC+3TC | 192 | 114/192 | 98/192 |  | 61/95 | 53/97 |  | 47/95 | 51/97 |  | 63 | 77 |  | 172.84 (131.13)/136 | 252.59 (198.31)/110 |
|  | EFV+TDF+FTC | 193 | 137/193 | 113/193 |  | 62/83 | 75/110 |  | 49/83 | 64/110 |  | 44 | 59 |  | 148.24 (101.02)/156 | 228.79 (123.72)/128 |
| ECHO | RPV+TDF+FTC | 346 | 285/346 | 265/346 |  | 162/181 | 125/165 |  | ‒ | ‒ |  | 50 | ‒ |  | 195.5 (151.7)/346 | 220.7 (167.1)/346 |
|  | EFV+TDF+FTC | 344 | 281/344 | 265/344 |  | 136/163 | 149/181 |  | ‒ | ‒ |  | 56 | ‒ |  | 181.6 (156.9)/344 | 226.7 (188.9)/344 |
| ENCORE1 | EFV400+TDF+FTC | 321 | 266/321 | 264/321 |  | 180/214 | 86/107 |  | 161/197 | 103/124 |  | 26 | 41 |  | 186 (120.46)/311 | 235 (149.48)/297 |
|  | EFV+TDF+FTC | 309 | 242/309 | 246/309 |  | 159/202 | 83/107 |  | 154/197 | 92/112 |  | 34 | 51 |  | 161 (120.46)/295 | 209 (137.57)/284 |
| FLAMINGO | DTG+ABC+3TC | 79 | 71/79 | 65/79 ^a^ |  | 59/66 | 12/13 |  | 54/66 ^a^ | 11/13 |  | ‒ | ‒ |  | ‒ | ‒ |
|  | DTG+TDF+FTC | 163 | 146/163 | 129/163 ^a^ |  | 101/115 | 45/48 |  | 90/115 ^a^ | 39/48 |  | ‒ | ‒ |  | ‒ | ‒ |
| GS-US-236-0102 | EVG/c+TDF+FTC | 348 | 305/348 | 293/348 |  | ‒ | ‒ |  | 197/230 | 96/118 |  | 37 | 53 |  | 239 (167.2)/325 | 295 (213.3)/307 |
|  | EFV+TDF+FTC | 352 | 296/352 | 287/352 |  | ‒ | ‒ |  | 191/236 | 96/116 |  | 46 | 61 |  | 206 (153.4)/315 | 273 (189.7)/302 |
| GS-US-292-0104/0111 | EVG/c+TAF+FTC | 866 | 800/866 | 750/866 |  | 629/670 ^b^ | 171/196 ^b^ |  | ‒ | ‒ |  | 45 | 95 |  | 230 (177.3)/817 | 285 (203)/395  \|274 (184)/369 ^c^ |
|  | EVG/c+TDF+FTC | 867 | 784/867 | 739/867 |  | 610/672 ^b^ | 174/195 ^b^ |  | ‒ | ‒ |  | 71 | 113 |  | 211 (170.1)/804 | 271 (208.1)/384  \|260 (179.6)/369 ^c^ |
| GS-US-380-1489 | BIC+TAF+FTC | 314 | 290/314 | 276/314 |  | 244/261 | 46/53 |  | 233/261 | 43/53 |  | 19 | 36 |  | 233 (185.2)/294 | 287 (207)/276 |
|  | DTG+ABC+3TC | 315 | 293/315 | 283/315 |  | 248/265 | 45/50 |  | 241/265 | 42/50 |  | 16 | 31 |  | 229 (188.8)/300 | 288 (247)/286 |
| GS-US-380–1490 | BIC+TAF+FTC | 320 | 286/320 | 269/320 |  | 229/254 | 57/66 |  | 215/254 | 54/66 |  | 28 | 48 |  | 180 (166.6)/290 | 237 (204.2)/269 |
|  | DTG+TAF+FTC | 325 | 302/325 | 281/325 |  | 251/271 | 51/54 |  | 234/271 | 47/54 |  | 20 | 36 |  | 201 (166.4)/304 | 281 (209.3)/285 |
| SINGLE | DTG+ABC+3TC | 414 | 364/414 | 332/414 |  | 253/280 | 111/134 |  | 237/280 | 95/134 |  | 51 | 72 |  | 267.5 (192.30)/368 | 322.6 (205.35)/343 |
|  | EFV+TDF+FTC | 419 | 338/419 | 303/419 |  | 238/288 | 100/131 |  | 209/288 | 94/131 |  | 84 | 109 |  | 209.5 (164.37)/344 | 286.0 (195.70)/309 |
| SPRING-2 | DTG+ABC+3TC | 169 | 145/169 | 125/169 |  | ‒ | ‒ |  | ‒ | ‒ |  | ‒ | ‒ |  | ‒ | ‒ |
|  | DTG+TDF+FTC | 242 | 216/242 | 207/242 |  | ‒ | ‒ |  | ‒ | ‒ |  | ‒ | ‒ |  | ‒ | ‒ |
|  | RAL+ABC+3TC | 164 | 142/164 | 124/164 |  | ‒ | ‒ |  | ‒ | ‒ |  | ‒ | ‒ |  | ‒ | ‒ |
|  | RAL+TDF+FTC | 247 | 209/247 | 190/247 |  | ‒ | ‒ |  | ‒ | ‒ |  | ‒ | ‒ |  | ‒ | ‒ |
| STaR | RPV+TDF+FTC | 394 | 338/394 | 307/394 |  | 231/260 | 107/134 |  | 205/260 | 102/134 |  | 54 | 78 |  | 200 (158.6)/357 | 278 (189)/327 |
|  | EFV+TDF+FTC | 392 | 320/392 | 284/392 |  | 204/250 | 116/142 |  | 178/250 | 106/142 |  | 72 | 102 |  | 191 (144.3)/346 | 259 (191)/319 |
| STARTMRK | RAL+TDF+FTC | 281 | 241/281 | 228/281 |  | 111/127 | 130/154 |  | ‒ | ‒ |  | 24 | 36 |  | 189 (122.926)/258 | 239.6 (159.41)/249 |
|  | EFV+TDF+FTC | 282 | 230/282 | 222/282 |  | 114/139 | 116/143 |  | ‒ | ‒ |  | 35 | 50 |  | 163 (121.247)/251 | 224.8 (151.51)/243 |
| THRIVE | RPV+TDF+FTC | 204 | 172/204 | 182/204 |  | ‒ | ‒ |  | 239/288 ^d^ | 182/262 ^d^ |  | ‒ | 64/550 ^d^ |  | ‒ | ‒ |
|  | RPV+ABC+3TC | 35 | 31/35 | 27/35 |  | ‒ | ‒ |  | ‒ | ‒ |  | ‒ | ‒ |  | ‒ | ‒ |
|  | EFV+TDF+FTC | 202 | 165/202 | 169/202 |  | ‒ | ‒ |  | 205/255 ^d^ | 216/291 ^d^ |  | ‒ | 92/546 ^d^ |  | ‒ | ‒ |
|  | EFV+ABC+3TC | 33 | 28/33 | 28/33 |  | ‒ | ‒ |  | ‒ | ‒ |  | ‒ | ‒ |  | ‒ | ‒ |

^a^ Data excluded from analysis because of heterogeneity; ^b^ Data excluded from analysis because it cannot be connected to the network; ^c^ Data before "|" belongs to GS-US-292-0104 and after "|" belongs to GS-US-292-0111; ^d^ Pooled data of THRIVE and ECHO

ABC, abacavir; BIC, bictegravir; DTG, dolutegravir; EFV, efavirenz; EFV400, 400mg efavirenz; EVG/c, cobicistat-boosted elvitegravir; FTC, emtricitabine; RAL, raltegravir; RPV, rilpivirine; TAF, tenofovir alafenamide; TDF, tenofovir disoproxil fumarate; VS, virologic suppression; 3TC, lamivudine.

**Supplementary Table S4.** Data extracted from the included studies

| **Trial** | **Treatment** | **N** | **Death, n (%)** | |  | **Drug-related Death, n (%)** | |  | **AEs, n/N** | |  | **drug-related AEs, n/N** | |  | **SAEs, n/N** | |
| --- | --- | --- | --- | --- | --- | --- | --- | --- | --- | --- | --- | --- | --- | --- | --- | --- |
|  |  |  | **48-week** | **96-week** |  | **48-week** | **96-week** |  | **48-week** | **96-week** |  | **48-week** | **96-week** |  | **48-week** | **96-week** |
| ANRS 12313 NAMSAL | DTG+TDF+3TC | 310 | 6 (1.94%) | ‒ |  | ‒ | ‒ |  | ‒ | ‒ |  | ‒ | ‒ |  | ‒ | ‒ |
|  | EFV400+TDF+3TC | 303 | 7 (2.31%) | ‒ |  | ‒ | ‒ |  | ‒ | ‒ |  | ‒ | ‒ |  | ‒ | ‒ |
| ASSERT | EFV+ABC+3TC | 192 | ‒ | ‒ |  | ‒ | ‒ |  | ‒ | 172/192 |  | 98/192 | ‒ |  | ‒ | 31/192 |
|  | EFV+TDF+FTC | 193 | ‒ | ‒ |  | ‒ | ‒ |  | ‒ | 175/193 |  | 91/193 | ‒ |  | ‒ | 20/193 |
| ECHO | RPV+TDF+FTC | 346 | 0 (0%) | ‒ |  | 0 (0%) | ‒ |  | 303/346 | ‒ |  | ‒ | ‒ |  | 23/346 | ‒ |
|  | EFV+TDF+FTC | 344 | 1 (0.29%) | ‒ |  | 0 (0%) | ‒ |  | 317/344 | ‒ |  | ‒ | ‒ |  | 31/344 | ‒ |
| ENCORE1 | EFV400+TDF+FTC | 321 | 2 (0.62%) | 5 (1.56%) |  | 0 (0%) | 0 (0%) |  | 286/321 | 291/321 |  | 118/321 | 126/321 |  | 23/321 | 24/321 |
|  | EFV+TDF+FTC | 309 | 3(0.97%) | 5 (1.62%) |  | 1 (0.32%) | 1 (0.32%) |  | 273/309 | 285/309 |  | 146/309 | 148/309 |  | 22/309 | 32/309 |
| FLAMINGO | DTG+ABC+3TC | 79 | 0 (0%) | ‒ |  | 0 (0%) | ‒ |  | ‒ | ‒ |  | ‒ | ‒ |  | ‒ | ‒ |
|  | DTG+TDF+FTC | 163 | 0 (0%) | ‒ |  | 0 (0%) | ‒ |  | ‒ | ‒ |  | ‒ | ‒ |  | ‒ | ‒ |
| GS-US-236-0102 | EVG/c+TDF+FTC | 348 | 1 (0.29%) | 1 (0.29%) |  | ‒ | ‒ |  | ‒ | ‒ |  | ‒ | ‒ |  | ‒ | ‒ |
|  | EFV+TDF+FTC | 352 | 2 (0.57%) | 2 (0.57%) |  | ‒ | ‒ |  | ‒ | ‒ |  | ‒ | ‒ |  | ‒ | ‒ |
| GS-US-292-0104/0111 | EVG/c+TAF+FTC | 866 | 2(0.23%) | ‒ |  | 0 (0%) | ‒ |  | 779/866 ^a^ | ‒ |  | 346/866 ^a^ | 367/866 ^a^ |  | 69/866 ^a^ | 97/866 ^a^ |
|  | EVG/c+TDF+FTC | 867 | 3 (0.35) | ‒ |  | 0 (0%) | ‒ |  | 780/867 ^a^ | ‒ |  | 364/867 ^a^ | 398/867 ^a^ |  | 61/867 ^a^ | 87/867 ^a^ |
| GS-US-380-1489 | BIC+TAF+FTC | 314 | 0 (0%) | 2 (0.64%) |  | 0 (0%) | 0 (0%) |  | 265/314 | 292/314 |  | 82/314 | 89/314 |  | 19/314 | 36/314 |
|  | DTG+ABC+3TC | 315 | 0 (0%) | 0 (0%) |  | 0 (0%) | 0 (0%) |  | 283/315 | 302/315 |  | 127/315 | 127/315 |  | 25/315 | 39/315 |
| GS-US-380–1490 | BIC+TAF+FTC | 320 | 1 (0.31%) | 3 (0.94%) |  | 0 (0%) | 0 (0%) |  | 264/320 | 283/320 |  | 57/320 | 64/320 |  | ‒ | 55/320 |
|  | DTG+TAF+FTC | 325 | 2 (0.62%) | 3 (0.92%) |  | 0 (0%) | 0 (0%) |  | 272/325 | 288/325 |  | 83/325 | 92/325 |  | ‒ | 33/325 |
| SINGLE | DTG+ABC+3TC | 414 | 0 (0%) | 0 (0%) |  | 0 (0%) | 0 (0%) |  | 369/414 | ‒ |  | 180/414 | 182/414 |  | 37/414 | 44/414 |
|  | EFV+TDF+FTC | 419 | 2 (0.48%) | 2 (0.48%) |  | 0 (0%) | 0 (0%) |  | 387/419 | ‒ |  | 278/419 | 280/419 |  | 35/419 | 51/419 |
| SPRING-2 | DTG+ABC+3TC | 169 | ‒ | ‒ |  | ‒ | ‒ |  | ‒ | ‒ |  | ‒ | ‒ |  | ‒ | ‒ |
|  | DTG+TDF+FTC | 242 | ‒ | ‒ |  | ‒ | ‒ |  | ‒ | ‒ |  | ‒ | ‒ |  | ‒ | ‒ |
|  | RAL+ABC+3TC | 164 | ‒ | ‒ |  | ‒ | ‒ |  | ‒ | ‒ |  | ‒ | ‒ |  | ‒ | ‒ |
|  | RAL+TDF+FTC | 247 | ‒ | ‒ |  | ‒ | ‒ |  | ‒ | ‒ |  | ‒ | ‒ |  | ‒ | ‒ |
| STaR | RPV+TDF+FTC | 394 | 0 (0%) | 0 (0%) |  | 0 (0%) | 0 (0%) |  | 349/394 | 362/394 |  | ‒ | ‒ |  | ‒ | 36/394 |
|  | EFV+TDF+FTC | 392 | 2 (0.51%) | 2 (0.51%) |  | 0 (0%) | 0 (0%) |  | 365/392 | 368/392 |  | ‒ | ‒ |  | ‒ | 48/392 |
| STARTMRK | RAL+TDF+FTC | 281 | 2 (0.71%) | 3 (1.07%) |  | 0 (0%) | 0 (0%) |  | 253/281 | 266/281 |  | 124/281 | 132/281 |  | 28/281 | 40/281 |
|  | EFV+TDF+FTC | 282 | 0 (0%) | 0 (0%) |  | 0 (0%) | 0 (0%) |  | 272/282 | 275/282 |  | 217/282 | 220/282 |  | 27/282 | 34/282 |
| THRIVE | RPV+TDF+FTC | 204 | ‒ | 0/550 (0%) ^b^ |  | ‒ | ‒ |  | ‒ | ‒ |  | ‒ | ‒ |  | ‒ | 52/550 ^b^ |
|  | RPV+ABC+3TC | 35 | ‒ | ‒ |  | ‒ | ‒ |  | ‒ | ‒ |  | ‒ | ‒ |  | ‒ | ‒ |
|  | EFV+TDF+FTC | 202 | ‒ | 4/546 (0.73) ^b^ |  | ‒ | ‒ |  | ‒ | ‒ |  | ‒ | ‒ |  | ‒ | 61/546 ^b^ |
|  | EFV+ABC+3TC | 33 | ‒ | ‒ |  | ‒ | ‒ |  | ‒ | ‒ |  | ‒ | ‒ |  | ‒ | ‒ |

^a^ Data excluded from analysis because it cannot be connected to the network; ^b^ Pooled data of THRIVE and ECHO

ABC, abacavir; AE, adverse event; BIC, bictegravir; DTG, dolutegravir; EFV, efavirenz; EFV400, 400mg efavirenz; EVG/c, cobicistat-boosted elvitegravir; FTC, emtricitabine; N, number of participants; n, number of people; RAL, raltegravir; RPV, rilpivirine; SAE, serious adverse event; TAF, tenofovir alafenamide; TDF, tenofovir disoproxil fumarate; 3TC, lamivudine.

**R code**

library(gemtc)

setwd("D:\\aa")

# Read data

data <- read.csv("data.csv", sep=",", header=T)

treatments <- read.csv("treatments.csv", sep=",", header=T)

num <- read.csv("number.csv", sep=",", header=T)

# Network plot

network <- mtc.network(data, description="HIV network", treatments=treatments)

plot(network, use.description = TRUE, vertex.shape="circle", vertex.lable.color=

"black", vertex.color="pink", vertex.frame.color="pink",edge.color="grey77", dynamic.edge.width=FALSE, vertex.size=num$sampleSize)

#Model

# continuous values

model.ran <-mtc.model(network, likelihood="normal", link="identity", type="consistency/ume",

factor = 2.5, n.chain=4, linearModel="fixed",dic=TRUE)

# binary values

model.ran <-mtc.model(network, likelihood="binom", link="logit", type="consistency/ume", factor = 2.5,

n.chain=4, linearModel="fixed",dic=TRUE)

result.ran <- mtc.run(model.ran, n.adapt = 20000, n.iter = 100000, thin = 1)

# Potential scale reduction factor

gelman.diag(result.ran)

# Deviance information criterion

summary(result.ran)

# Table

table<-round(exp(relative.effect.table(result.ran)),2)

write.csv(table, "table.csv")

# Node-split analysis

result.ns <- mtc.nodesplit(network, comparisons= mtc.nodesplit.comparisons(network), linearModel="fixed", factor = 2.5, n.chain=4, n.adapt = 20000, n.iter = 100000, thin=1)

summary(result.ns)

# *I^2^*

result.anohe <- mtc.anohe(network, linearModel="fixed", likelihood="binom", link="logit", factor = 2.5, n.chain=4, n.adapt = 20000, n.iter = 100000, thin=1)

summary(result.anohe)

plot(summary(result.anohe), xlim=log(c(0.2, 6)))

# Ranks

ranks <- rank.probability(result.ran,preferredDirection=1/-1)

print(ranks)

print(SUCRA(ranks))

**Supplementary Table S5.** Articles excluded after screening full-text

| Reason | No | Auhor | Year | Title |
| --- | --- | --- | --- | --- |
| Participants not of interest | 1 | Maggiolo F, et al | 2002 | Strategic selective treatment in highly pre-treated HIV patients harbouring multiply resistant viruses |
|  | 2 | Arranz-Caso JA, et al | 2004 | Treatment of HIV-infected patients with a combination of efavirenz, nevirapine and nucleoside reverse transcriptase inhibitors. |
|  | 3 |  | 2010 | Recent FDA approvals and changes. |
|  | 4 | Menezes CN, et al | 2014 | A randomized clinical trial comparing metabolic parameters after 48 weeks of standard- and low-dose stavudine therapy and tenofovir disoproxil fumarate therapy in HIV-infected South African patients. |
|  | 5 | Gandhi RT, et al | 2006 | Effect of baseline- and treatment-related factors on immunologic recovery after initiation of antiretroviral therapy in HIV-1-positive subjects: Results from ACTG 384 |
|  | 6 | Dube MP, et al | 2007 | Long-term body fat outcomes in anti retroviral-naive participants randomized to nelfinavir or efavirenz or both plus dual nucleosides - Dual X-ray absorptiometry results from A5005s, a substudy of adult clinical trials group 384 |
|  | 7 | Riddler SA, et al | 2008 | Class-sparing regimens for initial treatment of HIV-1 infection. |
|  | 8 | Daar ES, et al | 2011 | Atazanavir plus ritonavir or efavirenz as part of a 3-drug regimen for initial treatment of HIV-1: A randomized trial |
|  | 9 | Sax PE, et al | 2011 | Abacavir/lamivudine versus tenofovir DF/emtricitabine as part of combination regimens for initial treatment of HIV: final results. |
|  | 10 | Grant PM, et al | 2013 | Early virologic response to abacavir/lamivudine and tenofovir/emtricitabine during ACTG A5202 |
|  | 11 | Smith KY, et al | 2014 | Outcomes by sex following treatment initiation with atazanavir plus ritonavir or efavirenz with abacavir/lamivudine or tenofovir/emtricitabine. |
|  | 12 | Bisson GP, et al | 2017 | Risk factors for early mortality on antiretroviral therapy in advanced HIV-infected adults |
|  | 13 | Steytler J, et al | 2017 | Analysis of Site Heterogeneity and HIV Outcomes Across Rural and Urban Study Sites in Phidisa II - A Multi-site Randomized Controlled Antiretroviral Treatment Trial in a South African Military Cohort |
|  | 14 | Pepperrell T, et al | 2019 | Time to rethink endpoints for new antiretroviral regimen registration? HIV RNA re-suppression in the ADVANCE trial |
| Treatments not of interest | 1 | MacArthur RD, et al | 2001 | The rationale and design of the CPCRA (Terry Beirn Community Programs for Clinical Research on AIDS) 058 FIRST (Flexible Initial Retrovirus Suppressive Therapies) trial |
|  | 2 | DeJesus E, et al | 2004 | Once-daily versus twice-daily lamivudine, in combination with zidovudine and efavirenz, for the treatment of antiretroviral-naive adults with HIV infection: a randomized equivalence trial |
|  | 3 | Furco A, et al | 2004 | 5-Year follow-up of once-daily combination therapy with FTC, ddI and EFV in treatment of naive HIV-infected adults (MONTANA ANRS 091 trial) |
|  | 4 | Clifford DB, et al | 2005 | Impact of efavirenz on neuropsychological performance and symptoms in HIV-infected individuals. |
|  | 5 | Moyle GJ | 2005 | Abacavir once or twice daily combined with once-daily lamivudine and efavirenz for the treatment of antiretroviral-naive HIV-infected adults: results of the Ziagen Once Daily in Antiretroviral Combination Study |
|  | 6 | Nuesch R, et al | 2006 | Monitoring the toxicity of antiretroviral therapy in resource limited settings: A prospective clinical trial cohort in Thailand. |
|  | 7 | Marcellin F, et al | 2008 | Depressive symptoms and exposure to efavirenz in West African HIV-infected adults |
|  | 8 | Ribaudo HJ, et al | 2008 | Efavirenz-based regimens in treatment-naive patients with a range of pretreatment HIV-1 RNA levels and CD4 cell counts |
|  | 9 | van den Berg-Wolf M, et al | 2008 | Virologic, immunologic, clinical, safety, and resistance outcomes from a long-term comparison of efavirenz-based versus nevirapine-based antiretroviral regimens as initial therapy in HIV-1-infected persons |
|  | 10 | Manosuthi W, et al | 2009 | A randomized trial comparing plasma drug concentrations and efficacies between 2 nonnucleoside reverse-transcriptase inhibitor-based regimens in HIV-infected patients receiving rifampicin: the N2R Study |
|  | 11 | Eron JJ Jr, et al | 2011 | Raltegravir once daily or twice daily in previously untreated patients with HIV-1: a randomised, active-controlled, phase 3 non-inferiority trial |
|  | 12 | Lartey M, et al | 2011 | Viral decay rates are similar in HIV-infected patients with and without TB coinfection during treatment with an Efavirenz-based regimen |
|  | 13 | Bonnet M, et al | 2013 | Incidence of paradoxical tuberculosis-associated immune reconstitution inflammatory syndrome and impact on patient outcome |
|  | 14 | Sinha S, et al | 2013 | Nevirapine versus efavirenz-based antiretroviral therapy regimens in antiretroviral-naive patients with HIV and tuberculosis infections in India: a pilot study |
|  | 15 | Wester CW, et al | 2014 | Large-scale gene-centric exploration of risk for NRTI/NNRTI-associated toxicities in Botswana |
|  | 16 | Ofotokun I, et al. | 2015 | Comparison of the metabolic effects of ritonavir-boosted darunavir or atazanavir versus raltegravir, and the impact of ritonavir plasma exposure: ACTG 5257 |
|  | 17 | Bussmann H, et al | 2015 | Long-term ART outcomes in botswana encouraging treatment as prevention approach |
|  | 18 | Cohan D, et al | 2015 | Efficacy and safety of lopinavir/ritonavir versus efavirenz-based antiretroviral therapy in HIV-infected pregnant Ugandan women |
|  | 19 | Li L, et al | 2018 | Dual therapy with lopinavir/ritonavir plus lamivudine could be a viable alternative for antiretroviral-therapy-naive adults with HIV-1 infection regardless of HIV viral load or subgenotype in resource-limited settings: A randomised, open-label and non-inferiority study from China |
|  | 20 | Sarkar J, et al | 2018 | Lamivudine plus tenofovir versus lamivudine plus adefovir for the treatment of hepatitis B virus in HIV-coinfected patients, starting antiretroviral therapy |
|  | 21 | Sued O, et al | 2019 | Safety and efficacy of dolutegravir-based ART in TB/HIV coinfected adults at week 48 |
|  | 22 | MacArthur RD, etal | 2006 | A comparison of three highly active antiretroviral treatment strategies consisting of non-nucleoside reverse transcriptase inhibitors, protease inhibitors, or both in the presence of nucleoside reverse transcriptase inhibitors as initial therapy (CPCRA 058 FIRST Study): a long-term randomised trial |
|  | 23 | Montaner JS, etal | 2006 | Efficacy, safety and pharmacokinetics of once-daily saquinavir soft-gelatin capsule/ritonavir in antiretroviral-naive, HIV-infected patients |
|  | 24 | Rizzardini G, etal | 2006 | An immunological comparison of third companion in advanced drug-naive HIV-infected patients |
|  | 25 |  | 2008 | Ninety-six week data released on TMC278 |
|  | 26 | Bussmann H, etal | 2009 | Response to zidovudine/didanosine-containing combination antiretroviral therapy among HIV-1 subtype C-infected adults in Botswana: two-year outcomes from a randomized clinical trial |
|  | 27 | Sax PE, etal | 2009 | Abacavir-lamivudine versus tenofovir-emtricitabine for initial HIV-1 therapy |
|  | 28 | Cohen C, etal | 2010 | Pooled week 48 safety and efficacy results from the ECHO and THRIVE phase III trials comparing TMC278 vs EFV in treatment-naive, HIV-1-infected patients |
|  | 29 | Josephson F, etal | 2010 | The relation between treatment outcome and efavirenz, atazanavir or lopinavir exposure in the NORTHIV trial of treatment-naïve HIV-1 infected patients |
|  | 30 | Nct, etal | 2010 | PI or NNRTI as First-line Treatment of HIV in West Africa - the PIONA Trial |
|  | 31 | Wester CW, etal | 2010 | Non-nucleoside reverse transcriptase inhibitor outcomes among combination antiretroviral therapy-treated adults in Botswana |
|  | 32 | Nelson M, etal | 2011 | Pooled week-48 safety and efficacy results from ECHO and THRIVE Phase III trials comparing TMC278 vs EFV in treatment-naive HIV-1-infected patients receiving FTC/TDF |
|  | 33 | Hodder S, etal | 2012 | Effect of gender and race on the week 48 findings in treatment-naive, HIV-1-infected patients enrolled in the randomized, phase III trials ECHO and THRIVE |
|  | 34 | Molina JM, etal | 2012 | Rilpivirine efficacy, virology and safety in ARV treatmentnaïve patients with viral load5100,000 HIV-1 RNA c/mL: ECHO and THRIVE 96-week results |
|  | 35 | Nelson M, etal | 2012 | Efficacy and safety of rilpivirine in treatment-naive, HIV-1-infected patients with hepatitis B virus/hepatitis C virus coinfection enrolled in the Phase III randomized, double-blind ECHO and THRIVE trials |
|  | 36 | Orkin C, etal | 2012 | Poster Abstracts |
|  | 37 | Andersson LM, etal | 2013 | Lopinavir/ritonavir, atazanavir/ritonavir, and efavirenz in antiretroviral-naïve HIV-1-infected individuals over 144 weeks: an open-label randomized controlled trial |
|  | 38 | Molina JM, etal | 2013 | Rilpivirine vs. efavirenz in HIV-1 patients with baseline viral load 100,000 copies/ml or less: week 48 phase III analysis |
|  | 39 | Lima VD, etal | 2014 | Comparing the efficacy of efavirenz and boosted lopinavir using viremia copy-years |
|  | 40 | Dutertre M, etal | 2017 | Initiation of Antiretroviral Therapy Containing Integrase Inhibitors Increases the Risk of IRIS Requiring Hospitalization |
|  | 41 | Sinha S, etal | 2017 | Nevirapine- versus Efavirenz-based antiretroviral therapy regimens in antiretroviral-naive patients with HIV and Tuberculosis infections in India: a multi-centre study |
|  | 42 | Jespersen S, etal | 2018 | Protease Inhibitors or NNRTIs as First-Line HIV-1 Treatment in West Africa (PIONA): A Randomized Controlled Trial |
|  | 43 | Murray M, etal | 2018 | Improvements in patient-reported outcomes of dolutegravir (DTG)-based second-line treatment compared to lopinavir/ ritonavir (LPV/r)-based treatment: results from the DAWNING study |
|  | 44 | Aboud M, etal | 2019 | Superior Efficacy of Dolutegravir (DTG) Plus 2 Nucleoside Reverse Transcriptase Inhibitors (NRTIs) Compared with lopinavir/ritonavir (LPV/r) Plus 2 NRTIs in Second-Line Treatment — 48-week Data from the DAWNING Study |
|  | 45 | Venter WDF, et al | 2019 | Efficacy and Safety of Tenofovir Disoproxil Fumarate Versus Low-Dose Stavudine Over 96 Weeks: a Multicountry Randomized, Noninferiority Trial |
|  | 46 | Porteiro N, et al | 2019 | Efficacy of dolutegravir (DTG) plus lamivudine (3TC) versus DTG plus tenofovir/emtricitabine (TDF/FTC) in antiretroviral treatment-naive adults with HIV-1 infection: 48-week subgroup results from the GEMINI studies in Latin American participants |
|  | 47 | Orkin C, et al | 2019 | Doravirine/Lamivudine/Tenofovir Disoproxil Fumarate is Non-inferior to Efavirenz/Emtricitabine/Tenofovir Disoproxil Fumarate in Treatment-naive Adults With Human Immunodeficiency Virus-1 Infection: Week 48 Results of the DRIVE-AHEAD Trial |
|  | 48 | Orkin C, et al | 2019 | Two-drug regimen of dolutegravir plus lamivudine (DTG+3TC) is noninferior to dolutegravir plus tenofovir/emtricitabine (DTG+TDF/FTC) at 48 weeks in antiretroviral treatment-naive adults with HIV-1 infection: subgroup analyses in the GEMINI studies. |
|  | 49 | Mussini C, et al | 2019 | A prospective randomized trial on abacavir/ lamivudine plus darunavir/ritonavir or raltegravir in HIV-positive drug-naive patients with CD4<200 cells/uL (the PRADAR study |
|  | 50 | Orkin C, et al | 2019 | Long-active cabotegravir + rilpivirine for HIV maintenance: FLAIR week-48 results. |
|  | 51 | Murray M, et al | 2019 | Patient views on long acting HIV treatment: Cabotegravir + rilpivirine as maintenance therapy (ATLAS 48 week results) |
|  | 52 | Hocqueloux L, et al | 2019 | Dolutegravir Monotherapy Versus Dolutegravir/Abacavir/Lamivudine for Virologically Suppressed People Living with Chronic Human Immunodeficiency Virus Infection: The Randomized Noninferiority MONotherapy of TiviCAY Trial |
| outcomes not of interest | 1 | Kim AC, et al | 2004 | Strongyloides stercoralis infection as a manifestation of immune restoration syndrome. |
|  | 2 | van Leth, F, et al | 2004 | Quality of life in patients treated with first-line antiretroviral therapy containing nevirapine and/or efavirenz. |
|  | 3 | Kassutto S, et al | 2005 | Incomplete HIV type 1 antibody evolution and seroreversion in acutely infected individuals treated with early antiretroviral therapy |
|  | 4 | Gallant JE, etal | 2006 | Investigating new antiretroviral combinations - Reply to Carr |
|  | 5 | Markowitz M, etal | 2007 | Rapid and durable antiretroviral effect of the HIV-1 integrase inhibitor raltegravir as part of combination therapy in treatment-naive patients with HIV-1 infection: Results of a 48-week controlled study |
|  | 6 | Domingos H, et al | 2009 | Metabolic Effects Associated to the Highly Active Antiretroviral Therapy (HAART) in AIDS Patients. |
|  | 7 | Haubrich RH, et al | 2009 | Metabolic outcomes in a randomized trial of nucleoside, nonnucleoside and protease inhibitor-sparing regimens for initial HIV treatment |
|  | 8 | Ingiliz P, et al | 2009 | Liver Damage Underlying Unexplained Transaminase Elevation in Human Immunodeficiency Virus-1 Mono-infected Patients on Antiretroviral Therapy |
|  | 9 | Lanzafame M, etal | 2010 | Raltegravir: is a 400 mg once-daily dose enough? |
|  | 10 | Lampe FC, et al | 2010 | Changes in Lipids and Lipoprotein Particle Concentrations After Interruption of Antiretroviral Therapy |
|  | 11 | Nelson M, etal | 2010 | Efficacy and safety of TMC278 in treatment-naïve, HIV-1-infected patients with HBV/HCV co-infection enrolled in the phase III ECHO and THRIVE trials |
|  | 12 | Hulgan T, etal | 2011 | European mitochondrial DNA haplogroups and metabolic changes during antiretroviral therapy in AIDS Clinical Trials Group Study A5142 |
|  | 13 | Tebas P, etal | 2011 | Results from the pooled DEXA substudies of the double-blind, randomised, Phase III trials comparing rilpivirine (RPV, TMC278) versus efavirenz (EFV) in treatment-naive, HIV-1-infected adults |
|  | 14 | Wohl D, et al | 2011 | Change in vitamin D levels smaller, and risk of development of severe vitamin D deficiency lower, among HIV-1-infected, treatment-naïve adults receiving TMC278 compared with efavirenz: 48-week results from the Phase III ECHO trial |
|  | 15 | Hulgan T, etal | 2012 | T Cell Activation Markers and African Mitochondrial DNA Haplogroups among Non-Hispanic Black Participants in AIDS Clinical Trials Group Study 384 |
|  | 16 | Robertson K, etal | 2012 | Improved neuropsychological and neurological functioning across three antiretroviral regimens in diverse resource-limited settings: AIDS Clinical Trials Group study a5199, the International Neurological Study |
|  | 17 | Haas DW, et al | 2014 | Secondary metabolism pathway polymorphisms and plasma efavirenz concentrations in HIV-infected adults with CYP2B6 slow metabolizer genotypes |
|  | 18 | Touzard Romo F, et al | 2014 | Renal and metabolic toxicities following initiation of HIV-1 treatment regimen in a diverse, multinational setting: a focused safety analysis of ACTG PEARLS (A5175) |
|  | 19 | Callegaro A, etal | 2014 | Early clinical response and presence of viral resistant minority variants: a proof of concept study |
|  | 20 | Chen J, et al | 2014 | Anti-Retroviral Therapy Decreases but Does Not Normalize Indoleamine 2,3-Dioxygenase Activity in HIV-Infected Patients |
|  | 21 | Martin-Moreno JM, etal | 2014 | Erratum: Once-daily dolutegravir versus raltegravir in antiretroviral-naive adults with HIV-1 infection: 48 week results from the randomised, double-blind, non-inferiority SPRING-2 study (Lancet (2013) 381 (735-43)) |
|  | 22 | Safren SA, etal | 2014 | Psychosocial predictors of non-adherence and treatment failure in a large scale multi-national trial of antiretroviral therapy for HIV: data from the ACTG A5175/PEARLS trial |
|  | 23 | Yang S-P, etal | 2014 | Effectiveness of a reduced dose of efavirenz plus 2 NRTIs as maintenance antiretroviral therapy with the guidance of therapeutic drug monitoring |
|  | 24 | Barber TJ, etal | 2015 | FLAMINGO: still in the pink? |
|  | 25 | Blonk MI, etal | 2015 | Raltegravir in HIV-1-Infected Pregnant Women: Pharmacokinetics, Safety, and Efficacy |
|  | 26 | Ndjoyi-Mbiguino A, et al | 2015 | Performance of rapid HIV-1/HIV-2 INSTI (R) on plasma and capillary blood for serological diagnosis of non B subtypes and circulating recombinant forms of HIV-1 in Gabon |
|  | 27 | Dickinson L, etal | 2016 | Comprehensive Pharmacokinetic, Pharmacodynamic and Pharmacogenetic Evaluation of Once-Daily Efavirenz 400 and 600 mg in Treatment-Naïve HIV-Infected Patients at 96 Weeks: results of the ENCORE1 Study |
|  | 28 | Ma Q, et al | 2016 | Long-term efavirenz use is associated with worse neurocognitive functioning in HIV-infected patients |
|  | 29 | Erlandson KM, etal | 2017 | Insulin-Like Growth Factor Is Associated with Changes in Body Composition with Antiretroviral Therapy Initiation |
|  | 30 | Harper KN, etal | 2017 | Conference on Retroviruses and Opportunistic Infections 2017 highlights |
|  | 31 | Okoli C, etal | 2017 | Safety and efficacy of dolutegravir in treatment naive patients, 50 years and over: Subgroup analysis of 48-week results from SPRING-2, SINGLE, FLAMINGO and ARIA |
|  | 32 | Squires K, etal | 2017 | Influence of sex/gender and race on responses to raltegravir combined with tenofovir-emtricitabine in treatment-naive human immunodeficiency virus-1 infected patients: Pooled analyses of the STARTMRK and QDMRK studies |
|  | 33 | Serrano-Villar S, et al | 2017 | Different impact of raltegravir versus efavirenz on CD4/CD8 ratio recovery in HIV-infected patients. |
|  | 34 | Palumbo PJ, et al | 2018 | HIV Drug Resistance in Adults Receiving Early vs. Delayed Antiretroviral Therapy: HPTN 052. |
|  | 35 | Trevillyan JM, et al | 2018 | Changes in plasma lipidome following initiation of antiretroviral therapy |
|  | 36 | Adedeji TA, etal | 2019 | Effects of Highly Active Antiretroviral Therapy on Renal Function and Renal Phosphate Handling in African Adults with Advanced HIV and CKD |
|  | 37 | Elliot ER, et al | 2019 | Increased Dolutegravir Peak Concentrations in People Living With Human Immunodeficiency Virus Aged 60 and Over, and Analysis of Sleep Quality and Cognition. Clinical Infectious Diseases |
|  | 38 | Johnson M, etal | 2019 | Hepatic safety of bictegravir/emtricitabine/tenofovir alafenamide (B/F/TAF) |
|  | 39 | Podzamczer D, etal | 2019 | B/F/TAF versus ABC/DTG/3TC or DTG + F/TAF in treatmentnaïve adults with high baseline viral load or low baseline CD4 cell count: Week 96 results |
|  | 40 | Torgersen J, et al | 2019 | Impact of Efavirenz Metabolism on Loss to Care in Older HIV plus Africans. European Journal of Drug Metabolism and Pharmacokinetics |
|  | 41 | Wohl D, etal | 2019 | Patient-reported outcomes among adults living with HIV-1 who were randomly allocated to B/F/TAF versus DTG/ABC/3TC in two Phase 3 controlled clinical trials over 48 weeks |
| Not RCTs | 1 | De Santis M, etal | 2002 | Periconceptional exposure to efavirenz and neural tube defects |
|  | 2 |  | 2004 | For first-line antiretroviral therapy: wait and see |
|  | 3 | Berger P, etal | 2004 | Mycobacterium avium brain abscess at the initiation of highly active antiretroviral therapy |
|  | 4 | Mira JA, etal | 2008 | Efficacy of pegylated interferon plus ribavirin treatment in HIV/hepatitis C virus co-infected patients receiving abacavir plus lamivudine or tenofovir plus either lamivudine or emtricitabine as nucleoside analogue backbone |
|  | 5 | Parkes-Ratanshi R, etal | 2009 | Anaemia in a rural cohort of HIV-infected ugandans receiving either AZT- or non AZT-containing antiretroviral regimens |
|  | 6 | Apisarnthanarak A, etal | 2010 | Long-term outcomes of HIV-infected patients with <95% rates of adherence to nonnucleoside reverse-transcriptase inhibitors |
|  | 7 | Mbayiha AH, etal | 2010 | Evaluation of adult immunological outcomes from kimironko health center art program, 2007 to 2008 |
|  | 8 | Yotsumoto M, etal | 2010 | Central nervous system T-cell lymphoma in acquired immunodeficiency syndrome |
|  | 9 | Zhang XY, etal | 2010 | HIV microbicides: innovation and challenge |
|  | 10 | Fakunle O, etal | 2011 | Evaluation of Hiv Treatment Outcomes in Southwestern Nigeria |
|  | 11 | Swindells S, etal | 2011 | The critical need for alternative antiretroviral formulations, and obstacles to their development |
|  | 12 | von Reyn CF, etal | 2011 | Optimal treatment of Codisease due to HIV and tuberculosis |
|  | 13 | Chishinga N, etal | 2012 | Suppressed or unsuppressed HIV in adults on antiretroviral therapy in Zambia: who is at risk? |
|  | 14 | Croci L, etal | 2012 | Pharmacokinetic and safety of raltegravir in pregnancy |
|  | 15 | Franzeck F, etal | 2012 | cART prescription trends in a prospective HIV cohort in rural Tanzania from 2007 to 2011 |
|  | 16 | Arribas JR, etal | 2013 | Initial antiretroviral therapy: the dilemmas ahead |
|  | 17 | Mocroft A, etal | 2013 | CD4 cell count and viral load-specific rates of AIDS, non-AIDS and deaths according to current antiretroviral use |
|  | 18 | Smurzynski M, etal | 2013 | Factors associated with remaining on initial randomized efavirenz-containing regimens |
|  | 19 | Abramowicz M, etal | 2014 | Dolutegravir (Tivicay) for HIV |
|  | 20 | Lanzafame M, etal | 2014 | Efavirenz dose reduction in HIV-infected patients: a long-term follow-up |
|  | 21 | Ogbuagu O, etal | 2014 | Single-Pill Regimens for HIV-1 Infection |
|  | 22 | Bruyand M, etal | 2015 | Cancer Risk and Use of Protease Inhibitor or Nonnucleoside Reverse Transcriptase Inhibitor-Based Combination Antiretroviral Therapy: The D:A:D Study |
|  | 23 | Cohn J, etal | 2015 | Hit me with your best shot: dolutegravir - a space in the next WHO guidelines? |
|  | 24 | Ekat MH, etal | 2015 | ANTIRETROVIRAL THERAPY-RELATED NEPHROTOXICITY IN HIV INFECTED PATIENTS WITH LOW BODY MASS INDEX OUTPATIENT FOLLOW-UP IN BRAZZAVILLE, CONGO |
|  | 25 | Good BL, etal | 2015 | An unexpected interaction between warfarin and cobicistat-boosted elvitegravir |
|  | 26 | Samuel M, etal | 2015 | Drug treatment for adults with HIV infection |
|  | 27 |  | 2016 | Genvoya A New 4-Drug Combination for HIV |
|  | 28 | Artigues F, etal | 2016 | Neurological involvement in patients with acute/recent HIV-1 infection |
|  | 29 | Casado JL, etal | 2016 | Bone mineral density decline according to renal tubular dysfunction and phosphaturia in tenofovir-exposed HIV-infected patients |
|  | 30 | Kamara DA, etal | 2016 | Longitudinal analysis of the associations between antiretroviral therapy, viraemia and immunosuppression with lipid levels: the D:A:D study |
|  | 31 | Ohata PJ, etal | 2016 | Updates on HIV treatment and prevention from Asia's HIV symposium: the 18th Bangkok International Symposium on HIV Medicine |
|  | 32 | Bhagwat P, etal | 2017 | Changes in abdominal fat following antiretroviral therapy initiation in HIV-infected individuals correlate with waist circumference and self-reported changes |
|  | 33 | Katlama C, etal | 2017 | Individualized antiretroviral therapeutic approaches: less can be more |
|  | 34 | Lee GQ, etal | 2017 | Should Abacavir Be a First-Line Alternative for Adults With HIV in Sub-Saharan Africa? |
|  | 35 | Liu W, etal | 2017 | A Case Study in Personalized Medicine: Rilpivirine Versus Efavirenz for Treatment-Naive HIV Patients |
|  | 36 | Nozza S, etal | 2017 | Efficacy of elvitegravir/cobicistat/emtricitabine/tenofovir disoproxil fumarate as treatment for primary or recent HIV infection |
|  | 37 | Badii VS, etal | 2018 | Tenofovir-Based Highly Active Antiretroviral Therapy Is Associated with Superior CD4 T Cells Repopulation Compared to Zidovudine-Based HAART in HIV 1 Infected Adults |
|  | 38 | Rijnders BJA, etal | 2019 | Antiretroviral Monotherapy for HIV: Game Over or Future Perspectives? |
|  | 39 | Xie X, etal | 2019 | Repurposing an HIV Drug for Zika Virus Therapy |
| Phase not of interest | 1 | Nct, etal | 2009 | Study of the Safety and Efficacy of Stribild Versus Atripla in Human Immunodeficiency Virus, Type 1 (HIV-1) Infected, Antiretroviral Treatment-Naive Adults |
|  | 2 | Orkin C, etal | 2010 | Changes in patient-reported outcomes during the SENSE Trial: first-line treatment with 2 NRTIs plus etravirine or efavirenz |
|  | 3 | Castagna A, etal | 2011 | CHANGES IN PATIENT-REPORTED OUTCOMES DURING THE SENSE TRIAL: FIRST-LINE TREATMENT WITH 2 NRTIS PLUS ETRAVIRINE OR EFAVIRENZ |
|  | 4 | Fatkenheuer G, etal | 2011 | The SENSE trial week 48 results: etravirine shows lower prevalence of lipid abnormalities, compared to efavirenz in treatment-naive patients |
|  | 5 | Miralles Alvarez C, etal | 2011 | Changes in patient-reported outcomes during the SENSE Trial: first-line treatment with 2 NRTIs plus etravirine or efavirenz for 48 weeks |
|  | 6 | Grinsztejn B, etal | 2012 | A randomized multicentre open-label trial to estimate the efficacy and safety of two doses of raltegravir (RAL) to efavirenz (EFV) for the treatment of HIV-TB co-infected patients: results of the ANRS 12 180 Reflate TB trial |
|  | 7 | Maek-a-Nantawat W, etal | 2012 | Safety and efficacy of once-daily single generic fixed-drug combination tablet of tenofovir, lamivudine and efavirenz among HIV-infected Thais |
|  | 8 | Orkin C, etal | 2012 | Changes in patient-reported neuropsychiatric outcomes during the SENSE trial: First-line treatment with two nucleoside analogues plus etravirine or efavirenz |
|  | 9 | Sax PE, etal | 2017 | Randomized trial of bictegravir or dolutegravir with FTC/TAF for initial HIV therapy |
|  | 10 | Sax PE, etal | 2017 | Randomised trial of bictegravir or dolutegravir with FTC/TAF for initial HIV therapy |
| Not 48- or 96-week duration | 1 | Moggiolo F, etal | 2003 | Once-a-day therapy for HIV infection: A controlled, randomized study in antiretroviral-naive HIV-1-infected patients |
|  | 2 | Cooper V, etal | 2010 | The impact of once-nightly versus twice-daily dosing and baseline beliefs about HAART on adherence to efavirenz-based HAART over 48 weeks: The NOCTE study |
|  | 3 | Rockstroh JK, etal | 2011 | Long-term treatment with raltegravir or efavirenz combined with tenofovir/emtricitabine for treatment-naive human immunodeficiency virus-1-infected patients: 156-week results from STARTMRK |
|  | 4 | Uglietti A, etal | 2011 | The Sense Trial: Etravirine Shows Lower Prevalence and Severity of Neuropsychiatric Adverse Events Compared To Efavirenz in Treatment-Naive Patients |
|  | 5 | DeJesus E, etal | 2012 | Efficacy of raltegravir versus efavirenz when combined with tenofovir/emtricitabine in treatment-naïve HIV-1-infected patients: week-192 overall and subgroup analyses from STARTMRK |
|  | 6 | Uttayamakul S, etal | 2012 | CYP2B6 G516T and ABCB-1 C3435T polymorphisms: implications for efavirenz-associated liver toxicity in HIV/tuberculosis co-infected Thai adults |
|  | 7 | Menezes CN, etal | 2013 | The early effects of stavudine compared with tenofovir on adipocyte gene expression, mitochondrial DNA copy number and metabolic parameters in South African HIV-infected patients: a randomized trial |
|  | 8 | White KL, etal | 2014 | Efficacy of first-line ARV regimens: An exploratory “target not detected” analysis |
|  | 9 | Manosuthi W, etal | 2015 | Comparison of Thai Government Manufactured Tenofovir (Tenofovir Gpo300) with Privately Manufactured Tenofovir (Viread) Used Along with Lamivudine and Efavirenz To Treat Thai Hiv Patients |
|  | 10 | Arribas J, etal | 2017 | Significant efficacy and long term safety difference with TAF-based STR in naïve adults |
|  | 11 | Clarke A, etal | 2017 | Significant efficacy and long term safety difference with TAF-based STR in naive adults |
|  | 12 | Kaboggoza JP, etal | 2019 | A lower dose of efavirenz can be coadministered with rifampicin and isoniazid in tuberculosis patients |
| Had the same population | 1 |  | 2005 | Emtricitabine is more effective than stavudine for combination therapy in people with HIV |
|  | 2 | Izzedine H, etal | 2005 | Long-term renal safety of tenofovir disoproxil fumarate in antiretroviral-naïve HIV-1-infected patients. Data from a double-blind randomized active-controlled multicentre study |
|  | 3 | Cassetti I, etal | 2007 | The safety and efficacy of tenofovir DF in combination with lamivudine and efavirenz through 6 years in antiretroviral-naive HIV-1-infected patients |
|  | 4 | Euctr GB, etal | 2010 | A randomised, double-blind, placebo-controlled, clinical trial to compare the safety and efficacy of reduced dose efavirenz (EFV) with standard dose EFV plus two nucleotide reverse transcriptase inhibitors (N(t)RTI) in antiretroviral-naïve HIV-infected individuals over 96 weeks |
|  | 5 | Euctr PT, etal | 2011 | A Phase 3B, Randomized, Open-label Study to Evaluate the Safety and Efficacy of a Single Tablet Regimen of Emtricitabine/Rilpivirine/Tenofovir Disoproxil Fumarate Compared with a Single Tablet Regimen of Efavirenz/Emtricitabine/Tenofovir Disoproxil Fumarate in HIV-1 Infected, Antiretroviral Treatment-Naïve Adults |
|  | 6 | Nct, etal | 2011 | Study to Evaluate the Safety and Efficacy of a Single Tablet Regimen of Emtricitabine/Rilpivirine/Tenofovir Disoproxil Fumarate Compared With a Single Tablet Regimen of Efavirenz/Emtricitabine/Tenofovir Disoproxil Fumarate in HIV-1 Infected, Antiretroviral Treatment-Naive Adults |
|  | 7 | Cohen C, etal | 2012 | STAR Study: single tablet regimen emtricitabine/rilpivirine/ tenofovir DF is non-inferior to efavirenz/emtricitabine/ tenofovir DF in ART-naive adults |
|  | 8 | DeJesus E, etal | 2012 | Indirect treatment comparison of efficacy, safety and resistance of EVG/COBI/FTC/TDF (Quad) vs. RAL plus FTC/TDF in treatment- na ve HIV patients |
|  | 9 | Raffi F, etal | 2012 | Once-daily dolutegravir (DTG; S/GSK1349572) is non-inferior to raltegravir (RAL) in antiretroviral-naive adults: 48 week results from SPRING-2 (ING113086) |
|  | 10 | Cohen C, etal | 2013 | STAR: rilpivirine/Emtricitabine/TenofovirDF is non-inferior to Efavirenz/Emtricitabine/TenofovirDF in naive adult Latino, black & white subpopulations |
|  | 11 | Cohen C, etal | 2013 | STaR study: Single-tablet regimen rilpivirine/ emtricitabine/tenofovir DF maintains noninferiority to efavirenz/emtricitabine/tenofovir DF and has minimal impact on fasting lipids in ART-naive adults through week 96 |
|  | 12 | Rockstroh JK, etal | 2013 | Durable efficacy and safety of raltegravir versus efavirenz when combined with tenofovir/emtricitabine in treatment-naive HIV-1-infected patients: Final 5-year results from STARTMRK |
|  | 13 | Ryan R, etal | 2013 | Outcomes in older versus younger patients over 96 weeks in HIV-1- infected patients treated with rilpivirine or efavirenz in ECHO and THRIVE |
|  | 14 | Trottier B, etal | 2013 | Star study: Single-tablet regimen rilpivirine/ emtricitabine /tenofovir DF is non-inferior to efavirenz/emtricitabine /tenofovir DF in ART-naïve HIV-1 infected adults |
|  | 15 | Wilkins E, etal | 2013 | STaR Study: Single tablet regimen rilpivirine/emtricitabine/ tenofovir DF is non-inferior to efavirenz/emtricitabine/ tenofovir DF in ART-naïve adults regardless of baseline viral load and CD4 + count |
|  | 16 | Carey D, etal | 2014 | Efavirenz 400 mg daily remains non-inferior to 600 mg: 96 week data from the double-blind, placebo-controlled ENCORE1 study |
|  | 17 | Molina JM, etal | 2014 | Once-daily dolutegravir is superior to once-daily darunavir/ritonavir in treatment-naive HIV-1-positive individuals: 96 week results from FLAMINGO |
|  | 18 | Molina JM, etal | 2014 | Week 96 analysis of rilpivirine or efavirenz in HIV-1-infected patients with baseline viral load </= 100 000 copies/mL in the pooled ECHO and THRIVE phase 3, randomized, double-blind trials |
|  | 19 | Trottier B, etal | 2014 | Star study: Single tablet regimen Rilpivirine/Emtricitabine/Tenofovir DF maintains non-inferiority to Efavirenz/Emtricitabine/Tenofovir DF in ART-naive adults through week 96 |
|  | 20 | Wohl DA, etal | 2014 | A Randomized, Double-Blind Comparison of Single-Tablet Regimen Elvitegravir/Cobicistat/Emtricitabine/Tenofovir DF Versus Single-Tablet Regimen Efavirenz/Emtricitabine/Tenofovir DF for Initial Treatment of HIV-1 Infection: Analysis of Week 144 Results |
|  | 21 | Euctr BE, etal | 2015 | This study will test an experimental drug called GS-9883/emtricitabine/tenofovir alafenamide (GS-9883/F/TAF) for the treatment of HIV-1 infection |
|  | 22 | Wohl D, etal | 2015 | Tenofovir alafenamide (TAF) in a single-tablet regimen in initial HIV-1 therapy |
|  | 23 | Arenas-Pinto A, et al | 2016 | Increased risk of suicidal behaviour with use of efavirenz: results from the START trial |
|  | 24 | Clarke A, etal | 2016 | A randomized, double-blind comparison of tenofovir alafenamide (TAF) vs tenofovir disoproxil fumarate (TDF), each coformulated with elvitegravir, cobicistat and emtricitabine (E/C/F) for initial HIV-1 treatment: week 96 results |
|  | 25 | J ML, etal | 2016 | An Indirect Comparison of Efficacy and Safety of Elvitegravir/Cobicistat/Emtricitabine/Tenofovir Disoproxil Fumarate and Abacavir/Lamivudine + Dolutegravir in Initial Therapy |
|  | 26 | Nct, etal | 2016 | Efficacy and Safety of a Dolutegravir-based Regimen for the Initial Management of HIV Infected Adults in Resource-limited Settings |
|  | 27 | Oka S, etal | 2016 | Efficacy and safety of tenofovir alafenamide versus tenofovir disoproxil fumarate in treatment-naïve Asian adults |
|  | 28 | Villanueva JA, etal | 2016 | Influence of age on outcomes in HIV-positive adults initiating tenofovir alafenamide fumarate (TAF) versus tenofovir disoproxil fumarate (TDF) with elvitegravir, cobicistat and emtricitabine (E/C/F/TAF vs. E/C/F/TDF) |
|  | 29 | Wilkins EL, etal | 2016 | Patient-reported outcomes in the single-tablet regimen (STaR) trial of rilpivirine/emtricitabine/tenofovir disoproxil fumarate versus efavirenz/emtricitabine/tenofovir disoproxil fumarate in antiretroviral treatment-naive adults infected with HIV-1 through 48 weeks of treatment |
|  | 30 | Wohl D, etal | 2016 | A randomized, double-blind comparison of tenofovir alafenamide (TAF) versus tenofovir disoproxil fumarate (TDF), each co-formulated with elvitegravir, cobicistat and emtricitabine (E/C/F) for initial HIV-1 treatment: Week 96 results |
|  | 31 | Wohl D, etal | 2016 | Renal safety of tenofovir alafenamide in patients at high risk of kidney disease |
|  | 32 | Arribas JR, etal | 2017 | Brief Report: Randomized, Double-Blind Comparison of Tenofovir Alafenamide (TAF) vs Tenofovir Disoproxil Fumarate (TDF), Each Coformulated With Elvitegravir, Cobicistat, and Emtricitabine (E/C/F) for Initial HIV-1 Treatment: Week 144 Results |
|  | 33 | Gallant J, etal | 2017 | A Phase 3 randomized controlled clinical trial of bictegravir in a fixed dose combination, B/F/TAF, vs ABC/DTG/3TC in treatment-naive adults at Week 48 |
|  | 34 | Ward D, etal | 2017 | Week 96 efficacy and safety of tenofovir alafenamide (TAF) versus tenofovir disoproxil fumarate (TDF) in older, hivinfected treatment-naïve adults |
|  | 35 | Cournil A, etal | 2018 | Dolutegravir-versus an efavirenz 400 mg-based regimen for the initial treatment of HIV-infected patients in Cameroon: 48-week efficacy results of the NAMSAL ANRS 12313 trial |
|  | 36 | Podzamczer D, etal | 2018 | HIV Glasgow 2018, 28-31 October 2018, Glasgow, UK |
|  | 37 | Sax P, etal | 2018 | Virological outcomes of EVG/COBI/FTC/TDF and EVG/COBI/FTC/TAF in antiretroviral-naive HIV-1-infected participants with baseline HIV-1 RNA >/=1,000,000 copies/ml: a post hoc analysis of Phase III clinical trials |
|  | 38 | Stellbrink H, etal | 2018 | HIV Glasgow 2018, 28-31 October 2018, Glasgow, UK |
|  | 39 | Wohl D, etal | 2018 | Patient-Reported Symptoms Over 48 Weeks Among Participants in Randomized, Double-Blind, Phase III Non-inferiority Trials of Adults with HIV on Co-formulated Bictegravir, Emtricitabine, and Tenofovir Alafenamide versus Co-formulated Abacavir, Dolutegravir, and Lamivudine |
|  | 40 | Wohl DA, etal | 2019 | A Phase 3, Randomized, Controlled Clinical Trial of Bictegravir in a Fixed-Dose Combination, B/F/TAF, vs. ABC/DTG/3TC in Treatment-Naïve Adults at Week 96 |
|  | 41 | Waters L, etal | 2019 | A Phase 3, randomised, controlled clinical trial of bictegravir in a fixed-dose combination, B/F/TAF, versus ABC/DTG/3TC in treatment-naive adults at week 96 |
|  | 42 | Wohl D, etal | 2019 | HIV & Hepatitis in the Americas, 4-6 April 2019, Bogota, Colombia |


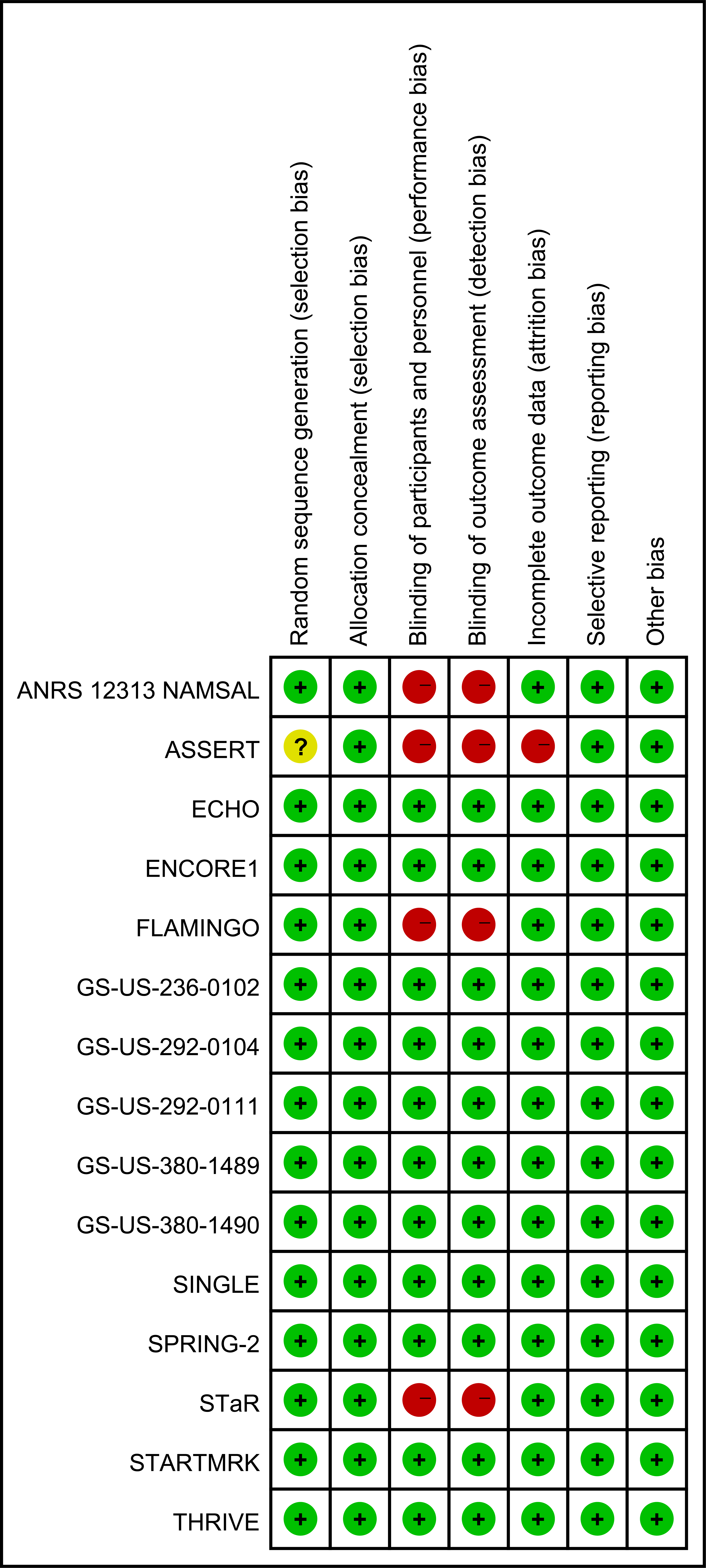


**Supplementary Figure S1.** Risk of bias summary

+, low risk of bias; ?, unclear risk of bias; -, high risk of bias.


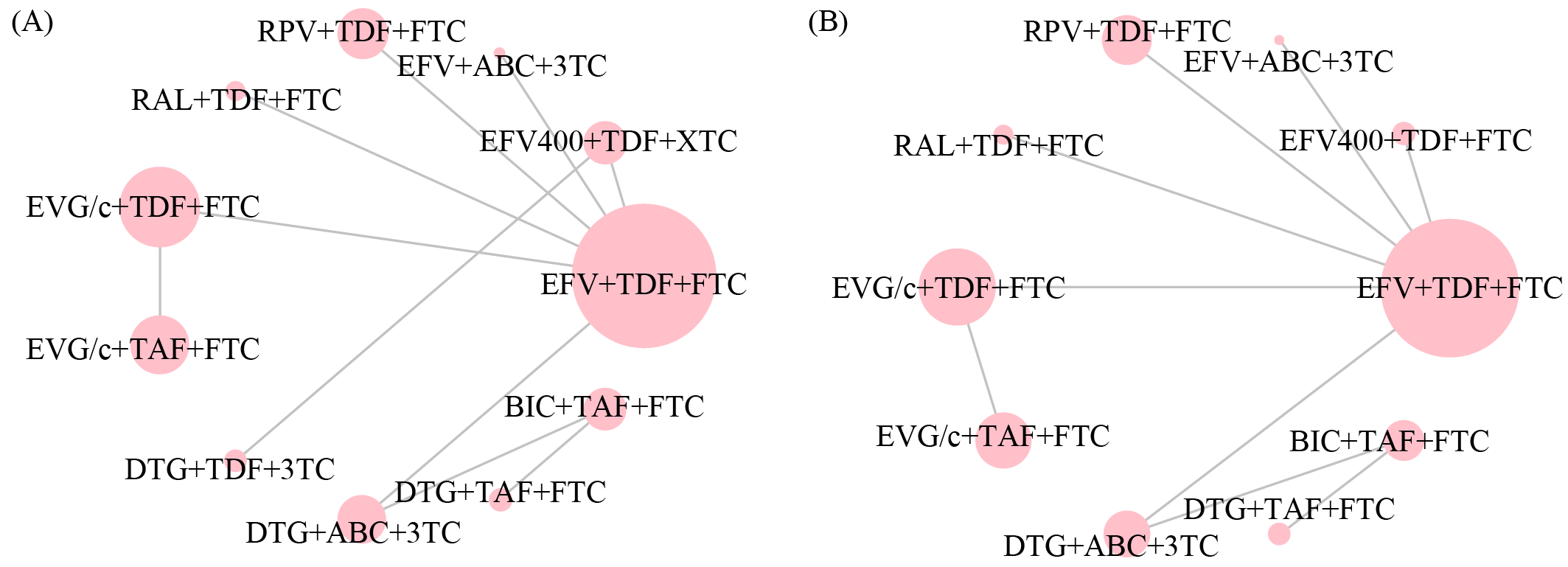


**Supplementary Figure S2.** Network plot of treatment comparisons for outcomes of CD4^+^ cell recovery (A) at 48 weeks and (B) at 96 weeks.

The larger the circle, the more participants included.

ABC, abacavir; BIC, bictegravir; DTG, dolutegravir; EFV, efavirenz; EFV400, 400mg efavirenz; EVG/c, cobicistat-boosted elvitegravir; FTC, emtricitabine; RAL, raltegravir; RPV, rilpivirine; TAF, tenofovir alafenamide; TDF, tenofovir disoproxil fumarate; XTC, FTC/3TC; 3TC, lamivudine.

**Supplementary Table S6**. The relative efficacy of antiretroviral regimens for CD4^+^ cell recovery at 48 weeks and 96 weeks.

| EFV+ TDF+FTC | **25.94 (2.61, 49.39)** | 23.87 (-19.07, 66.69) | 5.31 (-14.34, 24.93) | 14.84 (-12.58, 42.27) | 22.11 (-9.91, 54.12) | 36.15 (-1.37, 73.77) | ‒ | **36.64 (6.02, 67.46)** | **79.6 (20.01, 139.06)** | 35.69 (-12.57, 84.17) |
| --- | --- | --- | --- | --- | --- | --- | --- | --- | --- | --- |
| **-24.94 (-44.12, -5.7)** | EFV400+ TDF+XTC | -2.06 (-51.07, 46.58) | -20.64 (-51.17, 9.83) | -11.07 (-47.15, 25) | -3.85 (-43.54, 35.83) | 10.18 (-34.01, 54.23) | ‒ | 10.63 (-27.86, 49.36) | 53.62 (-10.21, 117.23) | 9.73 (-43.89, 63.43) |
| -24.61 (-51.73, 2.53) | 0.3 (-32.86, 33.58) | EFV+ ABC+3TC | -18.53 (-65.68, 28.69) | -9.06 (-59.93, 42.03) | -1.75 (-55.3, 51.76) | 12.29 (-44.58, 69.37) | ‒ | 12.78 (-40.04, 65.44) | 55.64 (-17.63, 128.97) | 11.83 (-52.63, 76.54) |
| -11.4 (-27.43, 4.64) | 13.54 (-11.42, 38.56) | 13.2  (-18.25, 44.72) | RPV+ TDF+FTC | 9.55  (-24.25, 43.22) | 16.79 (-20.77, 54.29) | 30.85 (-11.54, 73.17) | ‒ | 31.34 (-5.01, 67.79) | **74.21 (11.52, 136.93)** | 30.37 (-21.78, 82.69) |
| **-25.99 (-47.26, -4.8)** | -1.04 (-29.67, 27.42) | -1.39 (-35.82, 32.98) | -14.58 (-41.27, 12.03) | RAL+ TDF+FTC | 7.27  (-34.76, 49.32) | 21.33 (-25.06, 67.73) | ‒ | 21.8 (-19.3, 62.9) | 64.71 (-0.71, 129.97) | 20.87 (-34.68, 76.44) |
| **-33.03 (-57.79, -8.09)** | -8.06 (-39.44, 23.39) | -8.41 (-45.11, 28.43) | -21.61 (-51.1, 7.98) | -7 (-39.52, 25.71) | EVG/c+ TDF+FTC | 14.05 (-5.4, 33.45) | ‒ | 14.54 (-29.97, 58.86) | 57.53 (-10.25, 124.92) | 13.56 (-44.51, 71.82) |
| **-51.98 (-82.04, -21.95)** | -27.04 (-62.66, 8.62) | -27.34 (-67.91, 13.06) | **-40.56 (-74.61, -6.49)** | -25.97 (-62.74, 10.87) | **-18.99 (-35.93, -2.08)** | EVG/c+ TAF+FTC | ‒ | 0.49 (-47.97, 48.89) | 43.48 (-27.13, 113.41) | -0.52 (-61.69, 60.78) |
| **-55  (-83.07, -27.05)** | **-30.08 (-50.47, -9.72)** | -30.36 (-69.58, 8.55) | **-43.62 (-75.91, -11.52)** | -29.03 (-64.17, 6.14) | -22  (-59.57, 15.29) | -3.02 (-44.15, 37.95) | DTG+ TDF+3TC | ‒ | ‒ | ‒ |
| **-58.03 (-84.24, -31.73)** | **-33.08 (-65.45, -0.54)** | -33.39 (-71.13, 4.37) | **-46.59 (-77.3, -15.88)** | -32 (-65.74, 1.81) | -24.99 (-61.12, 11.09) | -6.03 (-45.88, 33.85) | -2.97 (-41.19, 35.38) | DTG+ ABC+3TC | 42.91 (-8.03, 93.85) | -0.99 (-38.32, 36.65) |
| **-83.04 (-131.14, -35.19)** | **-58.14 (-109.76, -6.72)** | **-58.39 (-113.53, -3.61)** | **-71.6 (-122.34, -21.32)** | **-57.06 (-109.51, -4.59)** | -50.02 (-104.01, 3.71) | -31.03 (-87.59, 25.3) | -28.02 (-83.6, 27.31) | -25.07 (-65.24, 15.08) | DTG+ TAF+FTC | **-43.8 (-78.33, -9.47)** |
| **-62.07 (-101.94, -22.33)** | -37.14 (-81.09, 6.99) | -37.51 (-85.64, 10.79) | **-50.66 (-93.59, -7.85)** | -36.06 (-81.06, 9.03) | -29.05 (-76.06, 17.82) | -10.08 (-59.93, 39.58) | -7.04 (-55.55, 41.4) | -4.05 (-34.16, 25.91) | 20.98 (-5.76, 47.82) | BIC+ TAF+FTC |

Data are mean differences (95% credible interval) of the antiretroviral regimen column versus the antiretroviral regimen row (e.g., the result of EFV+TDF+FTC versus EFV400+TDF+XTC is -24.94 with respect to CD4^+^ cell recovery at 48 weeks). Different antiretroviral regimens are on the diagonal. The values below the diagonal are the 48-week results and those above the diagonal are the 96-week results. Values in bold indicate statistically significant comparisons.

ABC, abacavir; BIC, bictegravir; DTG, dolutegravir; EFV, efavirenz; EFV400, 400mg efavirenz; EVG/c, cobicistat-boosted elvitegravir; FTC, emtricitabine; RAL, raltegravir; RPV, rilpivirine; TAF, tenofovir alafenamide; TDF, tenofovir disoproxil fumarate; XTC, FTC/3TC; 3TC, lamivudine.

**Supplementary Table S7.** Possibility of each treatment in each rank and the SUCRA value for each treatment for the CD4+ cell recovery outcome at 48 weeks.

| Treatment | Rank1 | Rank2 | Rank 3 | Rank 4 | Rank 5 | Rank 6 | Rank 7 | Rank 8 | Rank 9 | Rank 10 | Rank 11 | SUCRA |
| --- | --- | --- | --- | --- | --- | --- | --- | --- | --- | --- | --- | --- |
| EFV+TDF+FTC | 0.00 | 0.00 | 0.00 | 0.00 | 0.00 | 0.00 | 0.00 | 0.01 | 0.59 | 12.92 | 86.47 | 0.01 |
| EFV400+TDF+XTC | 0.00 | 0.09 | 0.38 | 1.08 | 4.86 | 12.98 | 23.11 | 26.60 | 21.98 | 8.43 | 0.50 | 0.33 |
| EFV+ABC+3TC | 0.16 | 0.56 | 1.29 | 2.99 | 7.43 | 13.14 | 17.96 | 19.36 | 20.70 | 12.72 | 3.69 | 0.33 |
| RPV+TDF+FTC | 0.00 | 0.00 | 0.00 | 0.03 | 0.20 | 0.94 | 3.44 | 9.60 | 24.89 | 52.87 | 8.01 | 0.15 |
| RAL+TDF+FTC | 0.04 | 0.26 | 0.78 | 2.24 | 6.96 | 14.55 | 22.56 | 23.87 | 19.63 | 8.36 | 0.76 | 0.35 |
| EVG/c+TDF+FTC | 0.01 | 0.78 | 2.36 | 4.59 | 11.85 | 29.95 | 20.99 | 15.11 | 9.97 | 3.97 | 0.42 | 0.43 |
| EVG/c+TAF+FTC | 8.80 | 13.70 | 15.11 | 21.88 | 26.15 | 8.39 | 3.63 | 1.68 | 0.55 | 0.10 | 0.02 | 0.70 |
| DTG+TDF+3TC | 11.12 | 16.06 | 16.75 | 24.24 | 18.44 | 8.86 | 3.21 | 1.10 | 0.21 | 0.03 | 0.00 | 0.73 |
| DTG+ABC+3TC | 4.19 | 15.75 | 35.75 | 24.64 | 12.59 | 4.76 | 1.58 | 0.54 | 0.17 | 0.03 | 0.00 | 0.75 |
| DTG+TAF+FTC | 73.13 | 13.08 | 6.21 | 3.45 | 1.88 | 1.03 | 0.56 | 0.35 | 0.20 | 0.08 | 0.02 | 0.94 |
| BIC+TAF+FTC | 2.54 | 39.73 | 21.36 | 14.86 | 9.64 | 5.41 | 2.97 | 1.78 | 1.11 | 0.48 | 0.10 | 0.76 |

Units of values except SUCRA values: %

ABC, abacavir; BIC, bictegravir; DTG, dolutegravir; EFV, efavirenz; EFV400, 400mg efavirenz; EVG/c, cobicistat-boosted elvitegravir; FTC, emtricitabine; RAL, raltegravir; RPV, rilpivirine; TAF, tenofovir alafenamide; TDF, tenofovir disoproxil fumarate; 3TC, lamivudine.

**Supplementary Table S8.** Possibility of each treatment in each rank and the SUCRA value for each treatment for the outcome of CD4^+^ cell recovery at 96 weeks.

| Treatment | Rank 1 | Rank 2 | Rank 3 | Rank 4 | Rank 5 | Rank 6 | Rank 7 | Rank 8 | Rank 9 | Rank 10 | SUCRA |
| --- | --- | --- | --- | --- | --- | --- | --- | --- | --- | --- | --- |
| EFV+TDF+FTC | 0.00 | 0.00 | 0.00 | 0.00 | 0.05 | 0.57 | 3.22 | 14.30 | 39.16 | 42.69 | 0.09 |
| EFV400+TDF+FTC | 1.49 | 6.97 | 10.65 | 16.71 | 18.80 | 18.48 | 15.10 | 8.26 | 2.69 | 0.87 | 0.53 |
| EFV+ABC+3TC | 3.97 | 11.29 | 9.96 | 11.86 | 11.55 | 11.67 | 11.97 | 10.23 | 6.61 | 10.89 | 0.48 |
| RPV+TDF+FTC | 0.02 | 0.13 | 0.45 | 1.36 | 3.34 | 7.40 | 14.28 | 23.64 | 26.77 | 22.63 | 0.20 |
| RAL+TDF+FTC | 0.44 | 2.24 | 4.12 | 7.41 | 11.05 | 14.81 | 18.51 | 19.69 | 11.32 | 10.41 | 0.35 |
| EVG/c+TDF+FTC | 0.19 | 2.89 | 9.35 | 11.50 | 16.18 | 18.03 | 16.97 | 12.38 | 6.72 | 5.80 | 0.44 |
| EVG/c+TAF+FTC | 8.36 | 22.31 | 16.45 | 17.22 | 13.63 | 10.03 | 6.21 | 3.00 | 1.95 | 0.85 | 0.67 |
| DTG+ABC+3TC | 1.64 | 17.26 | 28.19 | 19.56 | 14.14 | 9.60 | 5.71 | 2.62 | 0.98 | 0.30 | 0.67 |
| DTG+TAF+FTC | 83.71 | 7.80 | 3.51 | 1.93 | 1.19 | 0.78 | 0.50 | 0.29 | 0.25 | 0.06 | 0.96 |
| BIC+TAF+FTC | 0.18 | 29.11 | 17.32 | 12.46 | 10.07 | 8.65 | 7.53 | 5.60 | 3.57 | 5.50 | 0.61 |

Units of values except SUCRA values: %

ABC, abacavir; BIC, bictegravir; DTG, dolutegravir; EFV, efavirenz; EFV400, 400mg efavirenz; EVG/c, cobicistat-boosted elvitegravir; FTC, emtricitabine; RAL, raltegravir; RPV, rilpivirine; TAF, tenofovir alafenamide; TDF, tenofovir disoproxil fumarate; 3TC, lamivudine.


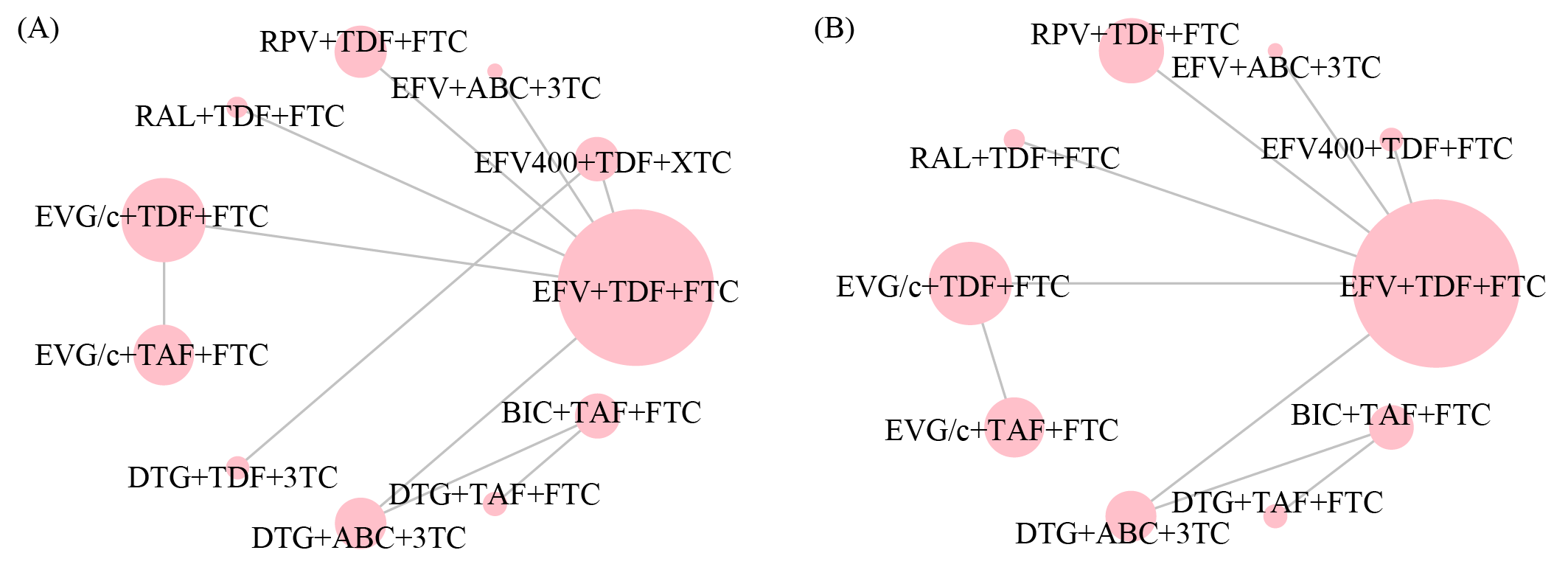


**Supplementary Figure S3.** Network plot of treatment comparisons for outcomes of discontinuations (A) at 48 weeks and (B) at 96 weeks.

The larger the circle, the more participants included.

ABC, abacavir; BIC, bictegravir; DTG, dolutegravir; EFV, efavirenz; EFV400, 400mg efavirenz; EVG/c, cobicistat-boosted elvitegravir; FTC, emtricitabine; RAL, raltegravir; RPV, rilpivirine; TAF, tenofovir alafenamide; TDF, tenofovir disoproxil fumarate; XTC, FTC/3TC; 3TC, lamivudine.

**Supplementary Table S9.** Relative results of antiretroviral regimens for discontinuations at 48 weeks and 96 weeks.

| EFV+  TDF+FTC | 0.74 (0.47, 1.15) | 1.52 (1, 2.32) | **0.68 (0.53, 0.86)** | 0.68 (0.42, 1.08) | 0.86 (0.57, 1.28) | 0.7 (0.43, 1.15) | ‒ | **0.6 (0.43, 0.83)** | 0.5 (0.23, 1.07) | 0.71 (0.39, 1.31) |
| --- | --- | --- | --- | --- | --- | --- | --- | --- | --- | --- |
| 1.41 (0.82, 2.43) | EFV400+ TDF+XTC | **2.06 (1.12, 3.8)** | 0.91 (0.55, 1.52) | 0.92 (0.48, 1.75) | 1.16 (0.64, 2.11) | 0.95  (0.49, 1.85) | ‒ | 0.81 (0.46, 1.42) | 0.68 (0.28, 1.64) | 0.96 (0.45, 2.05) |
| **0.6 (0.38, 0.95)** | **0.43 (0.21, 0.86)** | EFV+ ABC+3TC | **0.44 (0.27, 0.72)** | **0.45 (0.24, 0.83)** | 0.56 (0.31, 1) | **0.46 (0.24, 0.88)** | ‒ | **0.39 (0.23, 0.67)** | **0.33 (0.14, 0.78)** | **0.47 (0.22, 0.98)** |
| 1.29 (0.97, 1.71) | 0.92 (0.5, 1.68) | **2.14 (1.26, 3.65)** | RPV+ TDF+FTC | 1.01 (0.59, 1.69) | 1.27 (0.79, 2.02) | 1.04  (0.6, 1.81) | ‒ | 0.89 (0.59, 1.34) | 0.74 (0.33, 1.65) | 1.05 (0.55, 2.03) |
| 1.52 (0.89, 2.67) | 1.08 (0.5, 2.35) | **2.53 (1.25, 5.21)** | 1.18 (0.64, 2.21) | RAL+ TDF+FTC | 1.26 (0.68, 2.34) | 1.03 (0.52, 2.05) | ‒ | 0.88 (0.5, 1.57) | 0.74 (0.3, 1.81) | 1.05 (0.48, 2.26) |
| 1.27 (0.8, 2.02) | 0.9 (0.44, 1.83) | **2.1 (1.1, 4.02)** | 0.98 (0.57, 1.69) | 0.83 (0.4, 1.7) | RAL+ ABC+3TC | 0.82  (0.61, 1.1) | ‒ | 0.7 (0.41, 1.18) | 0.58 (0.24, 1.39) | 0.83 (0.4, 1.73) |
| **2.07  (1.13, 3.8)** | 1.47 (0.65, 3.3) | **3.43 (1.62, 7.32)** | 1.6 (0.82, 3.13) | 1.35 (0.6, 3.05) | **1.63 (1.11, 2.42)** | EVG/c+ TDF+FTC | ‒ | 0.85 (0.47, 1.55) | 0.71 (0.29, 1.78) | 1.01 (0.46, 2.23) |
| 2.19 (0.93, 5.3) | 1.55 (0.79, 3.12) | **3.64 (1.37, 9.81)** | 1.7 (0.68, 4.3) | 1.44 (0.51, 4.04) | 1.73 (0.65, 4.68) | 1.06 (0.37, 3.08) | DTG+ TDF+3TC | ‒ | ‒ | ‒ |
| **1.79 (1.23, 2.62)** | 1.27 (0.65, 2.46) | **2.97 (1.65, 5.38)** | 1.39 (0.86, 2.23) | 1.17 (0.6, 2.27) | 1.41 (0.78, 2.57) | 0.87 (0.42, 1.77) | 0.82 (0.31, 2.1) | DTG+ ABC+3TC | 0.84 (0.42, 1.66) | 1.19 (0.71, 1.98) |
| 2.18 (0.81, 5.91) | 1.55 (0.5, 4.81) | **3.62 (1.21, 10.8)** | 1.69 (0.6, 4.76) | 1.43 (0.46, 4.42) | 1.72 (0.58, 5.15) | 1.05 (0.33, 3.37) | 0.99 (0.26, 3.71) | 1.22 (0.49, 3.05) | DTG+ TAF+FTC | 1.42 (0.89, 2.27) |
| 1.49 (0.67, 3.29) | 1.06 (0.4, 2.76) | 2.47 (0.99, 6.14) | 1.15 (0.5, 2.67) | 0.98 (0.37, 2.54) | 1.18 (0.47, 2.94) | 0.72 (0.27, 1.95) | 0.68 (0.21, 2.19) | 0.83 (0.41, 1.66) | 0.68 (0.37, 1.24) | BIC+ TAF+FTC |

Data are odds ratios (95% credible interval) of the antiretroviral regimen column versus the antiretroviral regimen row. Different antiretroviral regimens are on the diagonal. The values below the diagonal are the 48-week results and those above the diagonal are the 96-week results. Values in bold indicate statistically significant comparisons.

ABC, abacavir; BIC, bictegravir; DTG, dolutegravir; EFV, efavirenz; EFV400, 400mg efavirenz; EVG/c, cobicistat-boosted elvitegravir; FTC, emtricitabine; RAL, raltegravir; RPV, rilpivirine; TAF, tenofovir alafenamide; TDF, tenofovir disoproxil fumarate; XTC, FTC/3TC; 3TC, lamivudine.

**Supplementary Table S10.** Possibility of each treatment in each rank and the SUCRA value for each treatment the outcome of discontinuations at 48 weeks

| Treatment | Rank 1 | Rank 2 | Rank 3 | Rank 4 | Rank 5 | Rank 6 | Rank 7 | Rank 8 | Rank 9 | Rank 10 | Rank 11 | SUCRA |
| --- | --- | --- | --- | --- | --- | --- | --- | --- | --- | --- | --- | --- |
| EFV+TDF+FTC | 0.00 | 0.00 | 0.00 | 0.00 | 0.05 | 0.44 | 2.70 | 11.26 | 30.11 | 54.67 | 0.77 | 0.16 |
| EFV400+TDF+XTC | 0.40 | 4.63 | 8.34 | 10.55 | 12.84 | 15.28 | 15.62 | 13.81 | 10.92 | 7.01 | 0.62 | 0.47 |
| EFV+ABC+3TC | 0.00 | 0.00 | 0.00 | 0.00 | 0.01 | 0.03 | 0.10 | 0.33 | 1.33 | 4.15 | 94.04 | 0.01 |
| RPV+TDF+FTC | 0.10 | 0.61 | 2.04 | 4.95 | 9.89 | 16.64 | 22.67 | 24.05 | 16.40 | 2.58 | 0.08 | 0.40 |
| RAL+TDF+FTC | 4.15 | 8.66 | 11.16 | 12.38 | 13.58 | 13.90 | 12.88 | 10.63 | 7.71 | 4.57 | 0.39 | 0.55 |
| EVG/c+TDF+FTC | 0.01 | 1.29 | 3.62 | 6.20 | 9.50 | 13.62 | 17.14 | 18.65 | 17.38 | 11.67 | 0.93 | 0.38 |
| EVG/c+TAF+FTC | 22.39 | 23.71 | 17.03 | 12.65 | 9.90 | 6.87 | 4.36 | 2.33 | 0.63 | 0.12 | 0.01 | 0.78 |
| DTG+TDF+3TC | 33.49 | 19.43 | 12.16 | 9.03 | 7.69 | 5.91 | 4.43 | 3.31 | 2.18 | 2.01 | 0.35 | 0.78 |
| DTG+ABC+3TC | 3.87 | 12.99 | 22.32 | 24.14 | 18.37 | 10.81 | 5.09 | 1.92 | 0.46 | 0.02 | 0.00 | 0.69 |
| DTG+TAF+FTC | 34.62 | 18.84 | 11.70 | 8.22 | 6.45 | 5.29 | 4.56 | 3.75 | 3.59 | 2.36 | 0.61 | 0.77 |
| BIC+TAF+FTC | 0.98 | 9.83 | 11.64 | 11.88 | 11.72 | 11.20 | 10.44 | 9.96 | 9.29 | 10.86 | 2.20 | 0.50 |

Units of values except SUCRA values: %

ABC, abacavir; BIC, bictegravir; DTG, dolutegravir; EFV, efavirenz; EFV400, 400mg efavirenz; EVG/c, cobicistat-boosted elvitegravir; FTC, emtricitabine; RAL, raltegravir; RPV, rilpivirine; TAF, tenofovir alafenamide; TDF, tenofovir disoproxil fumarate; 3TC, lamivudine.

**Supplementary Table S11.** Possibility of each treatment in each rank and the SUCRA value for each treatment the outcome of discontinuations at 96 weeks

| Treatment | Rank 1 | Rank 2 | Rank 3 | Rank 4 | Rank 5 | Rank 6 | Rank 7 | Rank 8 | Rank 9 | Rank 10 | SUCRA |
| --- | --- | --- | --- | --- | --- | --- | --- | --- | --- | --- | --- |
| EFV+TDF+FTC | 0.00 | 0.00 | 0.00 | 0.04 | 0.41 | 2.61 | 12.02 | 27.77 | 55.76 | 1.40 | 0.18 |
| EFV400+TDF+FTC | 5.77 | 8.36 | 9.44 | 11.98 | 14.15 | 16.00 | 14.16 | 12.80 | 6.65 | 0.70 | 0.52 |
| EFV+ABC+3TC | 0.00 | 0.00 | 0.01 | 0.02 | 0.07 | 0.21 | 0.56 | 1.60 | 4.33 | 93.21 | 0.01 |
| RPV+TDF+FTC | 4.85 | 10.79 | 15.68 | 20.62 | 20.85 | 15.49 | 8.41 | 3.27 | 0.04 | 0.00 | 0.62 |
| RAL+TDF+FTC | 11.35 | 12.90 | 12.15 | 13.76 | 13.82 | 13.14 | 10.69 | 8.23 | 3.59 | 0.36 | 0.61 |
| EVG/c+TDF+FTC | 0.14 | 1.57 | 3.23 | 5.15 | 8.96 | 14.72 | 22.04 | 26.11 | 16.17 | 1.91 | 0.34 |
| EVG/c+TAF+FTC | 9.92 | 11.48 | 11.14 | 12.97 | 14.27 | 15.77 | 14.74 | 6.50 | 2.83 | 0.38 | 0.59 |
| DTG+ABC+3TC | 11.81 | 25.73 | 26.55 | 17.11 | 10.24 | 5.55 | 2.38 | 0.61 | 0.02 | 0.00 | 0.76 |
| DTG+TAF+FTC | 55.02 | 13.23 | 8.06 | 6.21 | 5.32 | 4.33 | 3.64 | 2.57 | 1.34 | 0.29 | 0.84 |
| BIC+TAF+FTC | 1.15 | 15.94 | 13.74 | 12.15 | 11.91 | 12.18 | 11.36 | 10.55 | 9.27 | 1.76 | 0.53 |

Units of values except SUCRA values: %

ABC, abacavir; BIC, bictegravir; DTG, dolutegravir; EFV, efavirenz; EFV400, 400mg efavirenz; EVG/c, cobicistat-boosted elvitegravir; FTC, emtricitabine; RAL, raltegravir; RPV, rilpivirine; TAF, tenofovir alafenamide; TDF, tenofovir disoproxil fumarate; 3TC, lamivudine.


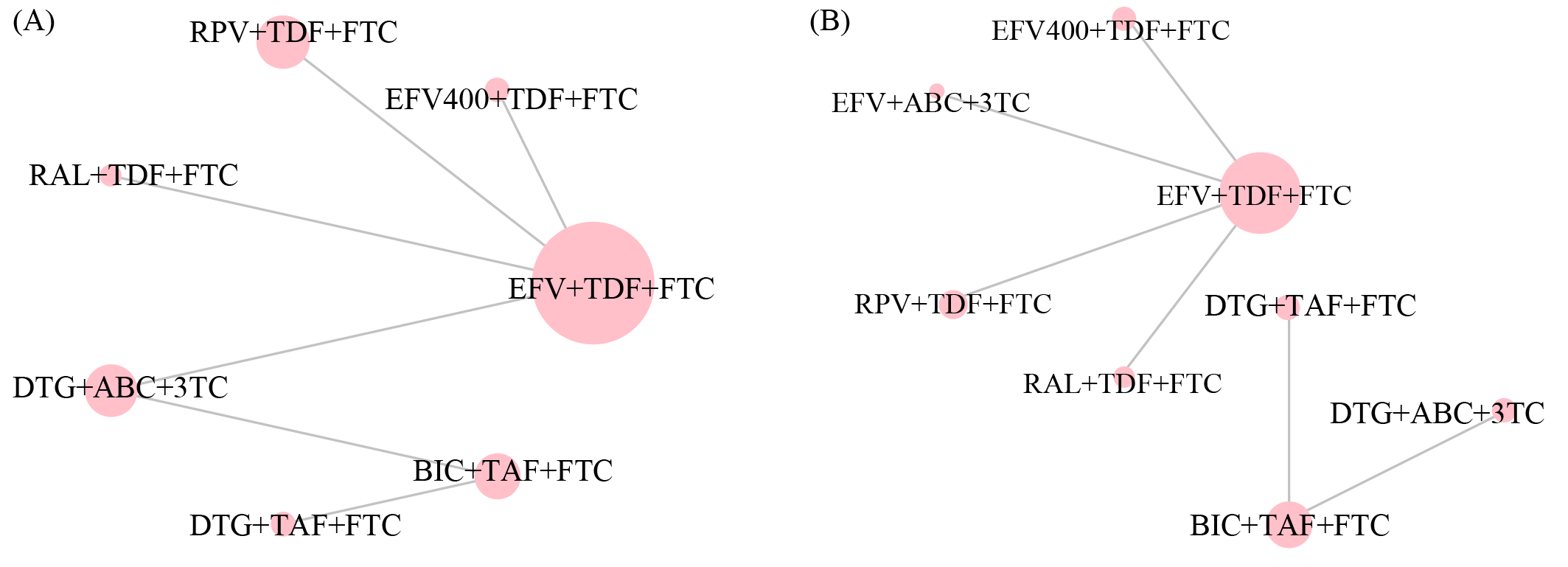


**Supplementary Figure S4.** Network plot of treatment comparisons for the outcome of adverse events (A) at 48 weeks and (B) at 96 weeks.

The larger the circle, the more participants included.

ABC, abacavir; BIC, bictegravir; DTG, dolutegravir; EFV, efavirenz; EFV400, 400mg efavirenz; FTC, emtricitabine; RAL, raltegravir; RPV, rilpivirine; TAF, tenofovir alafenamide; TDF, tenofovir disoproxil fumarate; 3TC, lamivudine.

**Supplementary Table S12.** Relative results of antiretroviral regimens for adverse events at 48 weeks and 96 weeks.

| EFV+  TDF+FTC | 0.81  (0.46, 1.43) | 0.88 (0.45, 1.74) | 0.74  (0.42, 1.27) | 0.44  (0.17, 1.08) | ‒ | ‒ | ‒ |
| --- | --- | --- | --- | --- | --- | --- | --- |
| 0.93  (0.57, 1.53) | EFV400+  TDF+FTC | 1.09  (0.45, 2.63) | 0.9  (0.41, 1.99) | 0.54  (0.18, 1.56) | ‒ | ‒ | ‒ |
| ‒ | ‒ | EFV+  ABC+3TC | 0.83  (0.35, 1.99) | 0.5  (0.15, 1.53) | ‒ | ‒ | ‒ |
| **1.71  (1.2, 2.45)** | 1.84 (1, 3.4) | ‒ | RPV+  TDF+FTC | 0.6  (0.2, 1.72) | ‒ | ‒ | ‒ |
| **3.07  (1.5, 6.85)** | **3.32  (1.38, 8.5)** | ‒ | 1.8  (0.8, 4.31) | RAL+ TDF+FTC | ‒ | ‒ | ‒ |
| 1.48  (0.92, 2.4) | 1.59  (0.8, 3.19) | ‒ | 0.86  (0.48, 1.57) | 0.48  (0.19, 1.14) | DTG+  ABC+3TC | 0.58  (0.24, 1.35) | 0.57  (0.27, 1.14) |
| **2.23  (1.01, 4.92)** | 2.4  (0.94, 6.12) | ‒ | 1.3  (0.55, 3.09) | 0.72  (0.24, 2.11) | 1.51  (0.8, 2.84) | DTG+  TAF+FTC | 0.98  (0.61, 1.6) |
| **2.43 (1.24, 4.8)** | **2.61  (1.13, 6.09)** | ‒ | 1.42  (0.66, 3.05) | 0.79  (0.28, 2.12) | **1.64  (1.02, 2.66)** | 1.09  (0.72, 1.65) | BIC+  TAF+FTC |

Data are odds ratios (95% credible interval) of the antiretroviral regimen column versus the antiretroviral regimen row. Different antiretroviral regimens are on the diagonal. The values below the diagonal are the 48-week results and those above the diagonal are the 96-week results. Values in bold indicate statistically significant comparisons.

ABC, abacavir; BIC, bictegravir; DTG, dolutegravir; EFV, efavirenz; EFV400, 400mg efavirenz; FTC, emtricitabine; RAL, raltegravir; RPV, rilpivirine; TAF, tenofovir alafenamide; TDF, tenofovir disoproxil fumarate; 3TC, lamivudine.

**Supplementary Table S13.** Possibility of each treatment in each rank and the SUCRA value for each treatment for the outcome of adverse events at 48 weeks

| Treatment | Rank 1 | Rank 2 | Rank 3 | Rank 4 | Rank 5 | Rank 6 | Rank 7 | SUCRA |
| --- | --- | --- | --- | --- | --- | --- | --- | --- |
| EFV+TDF+FTC | 0.00 | 0.00 | 0.17 | 0.64 | 3.89 | 59.66 | 35.64 | 0.12 |
| EFV400+TDF+FTC | 0.02 | 0.23 | 0.93 | 2.13 | 7.25 | 28.97 | 60.47 | 0.09 |
| RPV+TDF+FTC | 2.05 | 14.92 | 14.84 | 38.24 | 28.36 | 1.52 | 0.07 | 0.53 |
| RAL+TDF+FTC | 63.13 | 11.37 | 17.77 | 5.05 | 2.43 | 0.21 | 0.04 | 0.88 |
| DTG+ABC+3TC | 0.06 | 0.75 | 6.63 | 31.52 | 50.80 | 7.68 | 2.56 | 0.39 |
| DTG+TAF+FTC | 14.35 | 28.61 | 31.97 | 15.81 | 6.35 | 1.74 | 1.18 | 0.70 |
| BIC+TAF+FTC | 20.41 | 44.12 | 27.68 | 6.61 | 0.92 | 0.22 | 0.05 | 0.79 |

Units of values except SUCRA values: %

ABC, abacavir; BIC, bictegravir; DTG, dolutegravir; EFV, efavirenz; EFV400, 400mg efavirenz; FTC, emtricitabine; RAL, raltegravir; RPV, rilpivirine; TAF, tenofovir alafenamide; TDF, tenofovir disoproxil fumarate; 3TC, lamivudine.

**Supplementary Table S14.** Possibility of each treatment in each rank and the SUCRA value for each treatment for the outcome of adverse events at 96 weeks

| Treatment | Rank 1 | Rank 2 | Rank 3 | Rank 4 | Rank 5 | SUCRA |
| --- | --- | --- | --- | --- | --- | --- |
| EFV+TDF+FTC | 0.04 | 1.63 | 14.41 | 43.56 | 40.36 | 0.19 |
| EFV400+TDF+FTC | 7.28 | 26.19 | 29.28 | 19.89 | 17.37 | 0.47 |
| EFV+ABC+3TC | 6.35 | 20.27 | 22.87 | 19.54 | 30.97 | 0.38 |
| RPV+TDF+FTC | 12.19 | 37.97 | 26.99 | 13.97 | 8.88 | 0.58 |
| RAL+TDF+FTC | 74.14 | 13.94 | 6.46 | 3.05 | 2.42 | 0.89 |
| Treatment | Rank 1 | Rank 2 | Rank 3 | SUCRA |  |  |
| DTG+ABC+3TC | 3.96 | 7.90 | 88.14 | 0.08 |  |  |
| DTG+TAF+FTC | 46.27 | 44.45 | 9.28 | 0.68 |  |  |
| BIC+TAF+FTC | 49.77 | 47.65 | 2.58 | 0.74 |  |  |

Units of values except SUCRA values: %

ABC, abacavir; BIC, bictegravir; DTG, dolutegravir; EFV, efavirenz; EFV400, 400mg efavirenz; FTC, emtricitabine; RAL, raltegravir; RPV, rilpivirine; TAF, tenofovir alafenamide; TDF, tenofovir disoproxil fumarate; 3TC, lamivudine.


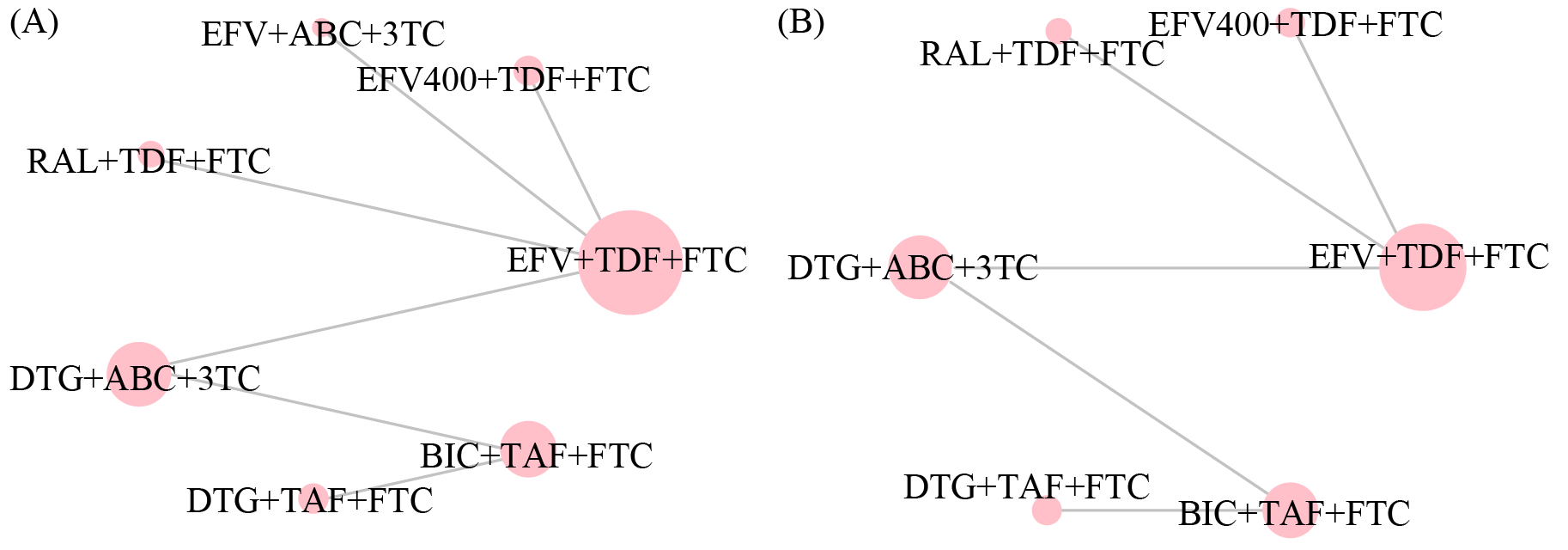


**Supplementary Figure S5.** Network plot of treatment comparisons for the outcome of drug-related adverse events (A) at 48 weeks and (B) at 96 weeks.

The larger the circle, the more participants included.

ABC, abacavir; BIC, bictegravir; DTG, dolutegravir; EFV, efavirenz; EFV400, 400mg efavirenz; FTC, emtricitabine; RAL, raltegravir; RPV, rilpivirine; TAF, tenofovir alafenamide; TDF, tenofovir disoproxil fumarate; 3TC, lamivudine.

**Supplementary Table S15**. Relative results of antiretroviral regimens for drug-related adverse events at 48 weeks and 96 weeks.

| EFV+  TDF+FTC | **0.7  (0.51, 0.96)** | ‒ | **0.25  (0.17, 0.36)** | **0.39  (0.29, 0.51)** | **0.36  (0.2, 0.64)** | **0.23  (0.15, 0.35)** |
| --- | --- | --- | --- | --- | --- | --- |
| **1.54  (1.12, 2.13)** | EFV400+  TDF+FTC | ‒ | **0.35  (0.22, 0.57)** | **0.55  (0.36, 0.85)** | **0.51  (0.27, 0.98)** | **0.32  (0.19, 0.55)** |
| 0.86  (0.57, 1.28) | **0.55  (0.33, 0.92)** | EFV+  ABC+3TC | ‒ | ‒ | ‒ | ‒ |
| **4.25  (2.96, 6.14)** | **2.75  (1.7, 4.47)** | **4.97  (2.89, 8.55)** | RAL+ TDF+FTC | 1.56  (0.99, 2.49) | 1.45  (0.74, 2.86) | 0.91  (0.52, 1.62) |
| **2.59  (1.96, 3.43)** | **1.68  (1.1, 2.57)** | **3.03  (1.86, 4.96)** | **0.61  (0.38, 0.96)** | DTG+  ABC+3TC | 0.92  (0.56, 1.52) | **0.58  (0.42, 0.81)** |
| **3.13  (1.75, 5.61)** | **2.02  (1.05, 3.93)** | **3.66  (1.81, 7.42)** | 0.73  (0.37, 1.47) | 1.21  (0.72, 2.01) | DTG+  TAF+FTC | **0.63  (0.44, 0.91)** |
| **4.96  (3.2, 7.74)** | **3.22  (1.87, 5.55)** | **5.8  (3.21, 10.56)** | 1.17  (0.66, 2.07) | **1.92  (1.37, 2.69)** | **1.59  (1.09, 2.33)** | BIC+  TAF+FTC |

Data are odds ratios (95% credible interval) of the antiretroviral regimen column versus the antiretroviral regimen row. Different antiretroviral regimens are on the diagonal. The values below the diagonal are the 48-week results and those above the diagonal are the 96-week results. Values in bold indicate statistically significant comparisons.

ABC, abacavir; BIC, bictegravir; DTG, dolutegravir; EFV, efavirenz; EFV400, 400mg efavirenz; FTC, emtricitabine; RAL, raltegravir; RPV, rilpivirine; TAF, tenofovir alafenamide; TDF, tenofovir disoproxil fumarate; 3TC, lamivudine.

**Supplementary Table S16.** Possibility of each treatment in each rank and the SUCRA value for each treatment for the outcome of drug-related adverse events at 48 weeks

| Treatment | Rank 1 | Rank 2 | Rank 3 | Rank 4 | Rank 5 | Rank 6 | Rank 7 | SUCRA |
| --- | --- | --- | --- | --- | --- | --- | --- | --- |
| EFV+TDF+FTC | 0.00 | 0.00 | 0.00 | 0.00 | 0.28 | 77.67 | 22.05 | 0.13 |
| EFV400+TDF+FTC | 0.00 | 0.00 | 0.32 | 2.32 | 95.94 | 1.31 | 0.12 | 0.34 |
| EFV+ABC+3TC | 0.00 | 0.00 | 0.00 | 0.01 | 1.16 | 21.01 | 77.83 | 0.04 |
| RAL+TDF+FTC | 30.79 | 50.82 | 17.08 | 1.31 | 0.00 | 0.00 | 0.00 | 0.85 |
| DTG+ABC+3TC | 0.00 | 0.37 | 23.99 | 74.90 | 0.74 | 0.00 | 0.00 | 0.54 |
| DTG+TAF+FTC | 0.64 | 17.65 | 58.35 | 21.47 | 1.89 | 0.02 | 0.00 | 0.66 |
| BIC+TAF+FTC | 68.57 | 31.17 | 0.26 | 0.00 | 0.00 | 0.00 | 0.00 | 0.95 |

Units of values except SUCRA values: %

ABC, abacavir; BIC, bictegravir; DTG, dolutegravir; EFV, efavirenz; EFV400, 400mg efavirenz; FTC, emtricitabine; RAL, raltegravir; RPV, rilpivirine; TAF, tenofovir alafenamide; TDF, tenofovir disoproxil fumarate; 3TC, lamivudine

**Supplementary Table S17.** Possibility of each treatment in each rank and the SUCRA value for each treatment for the outcome of drug-related adverse events at 96 weeks

| Treatment | Rank 1 | Rank 2 | Rank 3 | Rank 4 | Rank 5 | Rank 6 | SUCRA |
| --- | --- | --- | --- | --- | --- | --- | --- |
| EFV+TDF+FTC | 0.00 | 0.00 | 0.00 | 0.00 | 1.46 | 98.54 | 0.00 |
| EFV400+TDF+FTC | 0.00 | 0.00 | 0.15 | 2.24 | 96.17 | 1.43 | 0.20 |
| RAL+TDF+FTC | 37.74 | 47.52 | 12.67 | 2.06 | 0.00 | 0.00 | 0.84 |
| DTG+ABC+3TC | 0.01 | 1.17 | 38.64 | 59.98 | 0.20 | 0.00 | 0.48 |
| DTG+TAF+FTC | 0.46 | 13.43 | 48.21 | 35.71 | 2.17 | 0.02 | 0.55 |
| BIC+TAF+FTC | 61.80 | 37.88 | 0.33 | 0.00 | 0.00 | 0.00 | 0.92 |

Units of values except SUCRA values: %

ABC, abacavir; BIC, bictegravir; DTG, dolutegravir; EFV, efavirenz; EFV400, 400mg efavirenz; FTC, emtricitabine; RAL, raltegravir; RPV, rilpivirine; TAF, tenofovir alafenamide; TDF, tenofovir disoproxil fumarate; 3TC, lamivudine


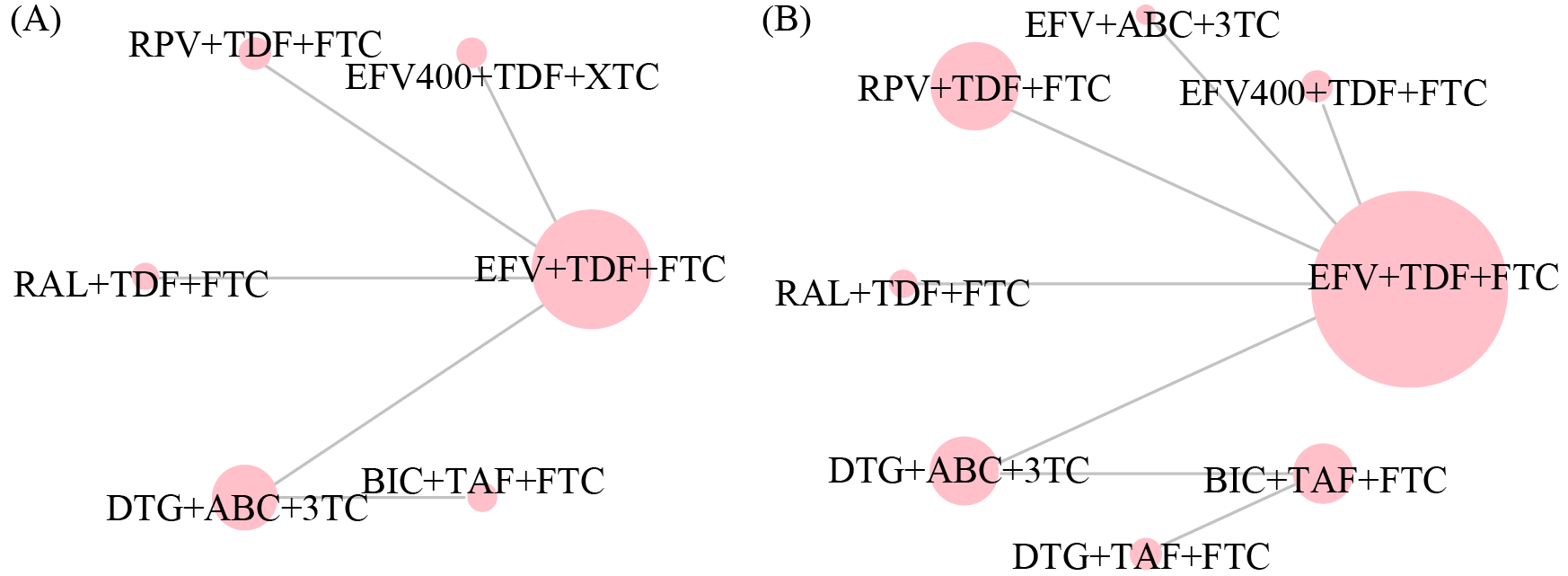


**Supplementary Figure S6.** Network plot of treatment comparisons for the outcome of serious adverse events (A) at 48 weeks and (B) at 96 weeks.

The larger the circle, the more participants included.

ABC, abacavir; BIC, bictegravir; DTG, dolutegravir; EFV, efavirenz; EFV400, 400mg efavirenz; FTC, emtricitabine; RAL, raltegravir; RPV, rilpivirine; TAF, tenofovir alafenamide; TDF, tenofovir disoproxil fumarate; 3TC, lamivudine.

**Supplementary Table S18.** Relative results of antiretroviral regimens for serious adverse events at 48 weeks and 96 weeks.

| EFV+  TDF+FTC | 0.7  (0.4, 1.21) | 1.68  (0.92, 3.12) | 0.78  (0.58, 1.05) | 1.21  (0.74, 1.98) | 0.86  (0.56, 1.32) | **0.43  (0.19, 0.94)** | 0.78  (0.41, 1.5) |
| --- | --- | --- | --- | --- | --- | --- | --- |
| 1  (0.54, 1.84) | EFV400+  TDF+FTC | **2.41  (1.07, 5.56)** | 1.12  (0.6, 2.12) | 1.74  (0.83, 3.67) | 1.23  (0.61, 2.51) | 0.61  (0.23, 1.62) | 1.13  (0.48, 2.66) |
| ‒ | ‒ | EFV+  ABC+3TC | **0.47  (0.23, 0.91)** | 0.72  (0.33, 1.57) | 0.51  (0.24, 1.07) | **0.25  (0.09, 0.68)** | 0.47  (0.19, 1.12) |
| 1.4  (0.8, 2.48) | 1.4  (0.61, 3.23) | ‒ | RPV+ TDF+FTC | 1.55  (0.87, 2.77) | 1.1  (0.65, 1.85) | 0.54  (0.23, 1.27) | 1  (0.49, 2.05) |
| 0.96  (0.55, 1.68) | 0.96  (0.43, 2.19) | ‒ | 0.68  (0.31, 1.52) | RAL+ TDF+FTC | 0.71  (0.37, 1.36) | **0.35  (0.14, 0.89)** | 0.65  (0.29, 1.46) |
| 0.93  (0.57, 1.51) | 0.93  (0.43, 2.03) | ‒ | 0.66  (0.31, 1.39) | 0.97  (0.46, 2.04) | DTG+  ABC+3TC | **0.5  (0.25, 0.97)** | 0.91  (0.56, 1.48) |
| ‒ | ‒ | ‒ | ‒ | ‒ | ‒ | DTG+ TAF+FTC | **1.84  (1.16, 2.96)** |
| 1.26  (0.57, 2.78) | 1.26  (0.47, 3.44) | ‒ | 0.9  (0.34, 2.37) | 1.31  (0.5, 3.45) | 1.35  (0.73, 2.54) | ‒ | BIC+ TAF+FTC |

Data are odds ratios (95% credible interval) of the antiretroviral regimen column versus the antiretroviral regimen row. Different antiretroviral regimens are on the diagonal. The values below the diagonal are the 48-week results and those above the diagonal are the 96-week results. Values in bold indicate statistically significant comparisons.

ABC, abacavir; BIC, bictegravir; DTG, dolutegravir; EFV, efavirenz; EFV400, 400mg efavirenz; FTC, emtricitabine; RAL, raltegravir; RPV, rilpivirine; TAF, tenofovir alafenamide; TDF, tenofovir disoproxil fumarate; 3TC, lamivudine.

**Supplementary Table S19.** Possibility of each treatment in each rank and the SUCRA value for each treatment for the outcome of serious adverse events at 48 weeks

| Treatment | Rank 1 | Rank 2 | Rank 3 | Rank 4 | Rank 5 | Rank 6 | SUCRA |
| --- | --- | --- | --- | --- | --- | --- | --- |
| EFV+TDF+FTC | 0.87 | 9.08 | 24.95 | 34.05 | 24.17 | 6.88 | 0.42 |
| EFV400+TDF+XTC | 10.47 | 17.06 | 16.24 | 14.49 | 16.43 | 25.31 | 0.43 |
| RPV+TDF+FTC | 46.18 | 26.82 | 12.54 | 6.69 | 4.56 | 3.20 | 0.79 |
| RAL+TDF+FTC | 7.08 | 14.39 | 15.73 | 15.61 | 18.26 | 28.93 | 0.38 |
| DTG+ABC+3TC | 1.26 | 9.06 | 17.56 | 19.36 | 25.96 | 26.80 | 0.32 |
| BIC+TAF+FTC | 34.15 | 23.59 | 12.97 | 9.79 | 10.61 | 8.89 | 0.67 |

Units of values except SUCRA values: %

ABC, abacavir; BIC, bictegravir; DTG, dolutegravir; EFV, efavirenz; EFV400, 400mg efavirenz; FTC, emtricitabine; RAL, raltegravir; RPV, rilpivirine; TAF, tenofovir alafenamide; TDF, tenofovir disoproxil fumarate; 3TC, lamivudine

**Supplementary Table S20.** Possibility of each treatment in each rank and the SUCRA value for each treatment for the outcome of serious adverse events at 96 weeks

| Treatment | Rank 1 | Rank 2 | Rank 3 | Rank 4 | Rank 5 | Rank 6 | Rank 7 | Rank 8 | SUCRA |
| --- | --- | --- | --- | --- | --- | --- | --- | --- | --- |
| EFV+TDF+FTC | 0.00 | 0.19 | 2.32 | 11.00 | 23.12 | 48.64 | 14.15 | 0.58 | 0.34 |
| EFV400+TDF+FTC | 14.24 | 33.18 | 19.26 | 13.32 | 10.42 | 5.67 | 3.36 | 0.55 | 0.71 |
| EFV+ABC+3TC | 0.04 | 0.20 | 0.52 | 1.04 | 1.82 | 3.45 | 16.49 | 76.44 | 0.05 |
| RPV+TDF+FTC | 3.71 | 19.13 | 28.69 | 22.92 | 19.51 | 4.62 | 1.32 | 0.10 | 0.64 |
| RAL+TDF+FTC | 0.21 | 1.31 | 3.11 | 5.43 | 8.06 | 13.00 | 49.80 | 19.08 | 0.21 |
| DTG+ABC+3TC | 0.32 | 6.15 | 20.44 | 27.69 | 24.35 | 13.69 | 6.46 | 0.91 | 0.51 |
| DTG+TAF+FTC | 81.35 | 11.95 | 4.05 | 1.32 | 0.78 | 0.42 | 0.12 | 0.01 | 0.96 |
| BIC+TAF+FTC | 0.13 | 27.90 | 21.61 | 17.29 | 11.93 | 10.51 | 8.30 | 2.33 | 0.59 |

Units of values except SUCRA values: %

ABC, abacavir; BIC, bictegravir; DTG, dolutegravir; EFV, efavirenz; EFV400, 400mg efavirenz; FTC, emtricitabine; RAL, raltegravir; RPV, rilpivirine; TAF, tenofovir alafenamide; TDF, tenofovir disoproxil fumarate; 3TC, lamivudine


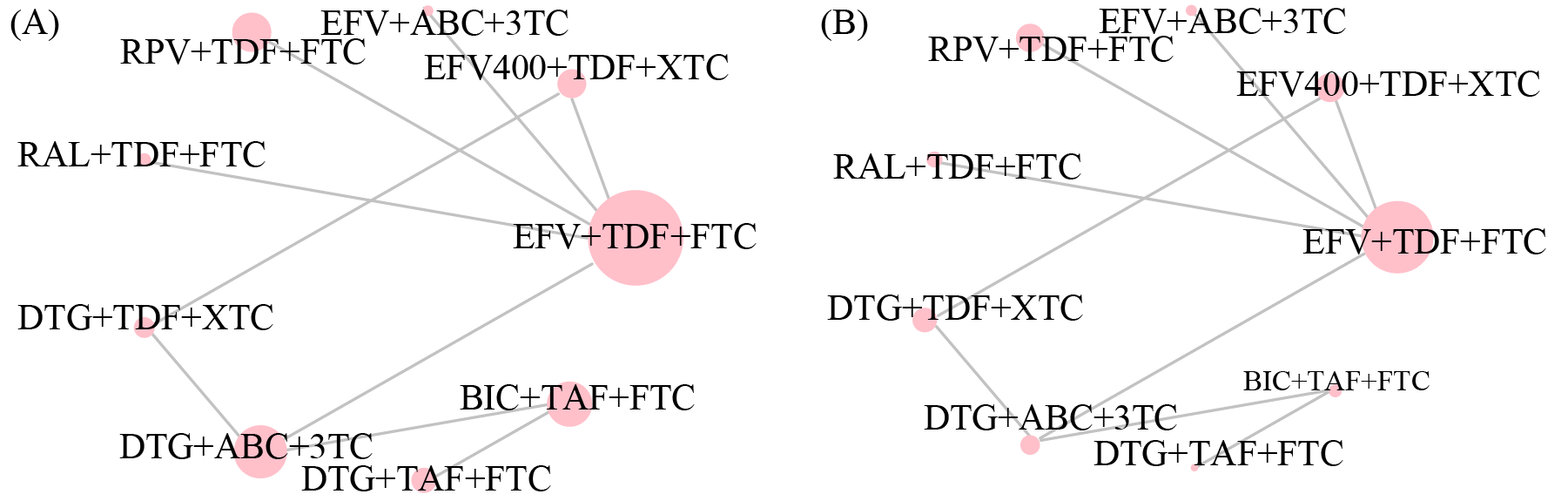


**Supplementary Figure S7.** Network plot of treatment comparisons for the subgroup analysis at 48 weeks.

The larger the circle, the more participants included.

ABC, abacavir; BIC, bictegravir; DTG, dolutegravir; EFV, efavirenz; EFV400, 400mg efavirenz; EVG/c, cobicistat-boosted elvitegravir; FTC, emtricitabine; RAL, raltegravir; RPV, rilpivirine; TAF, tenofovir alafenamide; TDF, tenofovir disoproxil fumarate; XTC, FTC/3TC; 3TC, lamivudine.


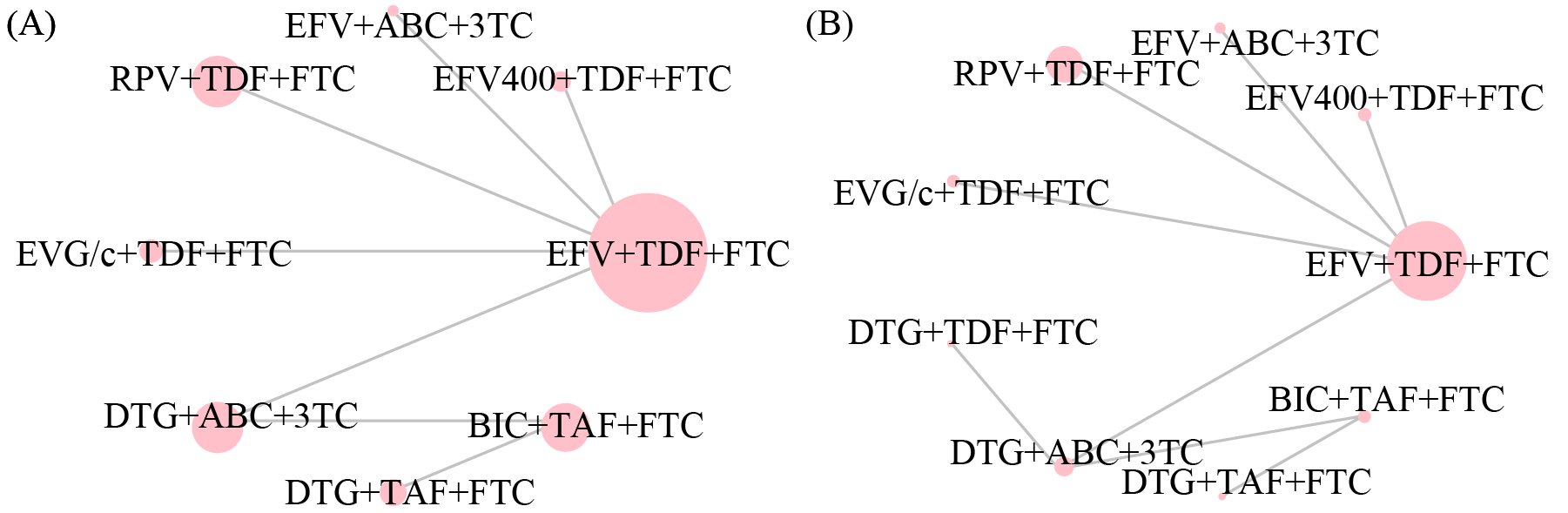


**Supplementary Figure S8.** Network plot of treatment comparisons for the subgroup analysis at 96 weeks.

The larger the circle, the more participants included.

ABC, abacavir; BIC, bictegravir; DTG, dolutegravir; EFV, efavirenz; EFV400, 400mg efavirenz; EVG/c, cobicistat-boosted elvitegravir; FTC, emtricitabine; RAL, raltegravir; RPV, rilpivirine; TAF, tenofovir alafenamide; TDF, tenofovir disoproxil fumarate; XTC, FTC/3TC; 3TC, lamivudine.

**Supplementary Table S21.** Relative results of antiretroviral regimens for the 48-week subgroup analysis.

| EFV+ TDF+FTC | 1.21  (0.63, 2.32) | **0.56  (0.32, 0.99)** | 0.76  (0.51, 1.12) | 1.27  (0.69, 2.34) | 1.49  (0.71, 3.15) | 1.49  (0.83, 2.71) | 3.13  (0.44, 24.92) | 1.07  (0.25, 4.21) |
| --- | --- | --- | --- | --- | --- | --- | --- | --- |
| 0.75  (0.47, 1.19) | EFV400+ TDF+XTC | 0.46  (0.19, 1.1) | 0.63  (0.29, 1.34) | 1.05  (0.43, 2.55) | 1.23  (0.83, 1.85) | 1.23  (0.53, 2.9) | 2.6  (0.33, 22.55) | 0.89  (0.19, 3.96) |
| 1.65  (0.87, 3.21) | 2.22  (0.99, 5) | EFV+ ABC+3TC | 1.35  (0.68, 2.7) | 2.26  (0.98, 5.24) | **2.65  (1.04, 6.84)** | **2.66  (1.18, 6.06)** | 5.58  (0.73, 48.11) | 1.91  (0.41, 8.41) |
| **0.57  (0.38, 0.84)** | 0.76  (0.41, 1.4) | **0.34  (0.16, 0.73)** | RPV+ TDF+FTC | 1.68  (0.81, 3.48) | 1.97  (0.84, 4.6) | 1.97  (0.98, 4.03) | 4.14  (0.56, 34.36) | 1.41  (0.32, 5.91) |
| 0.65  (0.32, 1.28) | 0.87  (0.38, 2) | 0.39  (0.15, 1) | 1.15  (0.51, 2.52) | RAL+ TDF+FTC | 1.18  (0.45, 3.09) | 1.18  (0.5, 2.75) | 2.48  (0.32, 21.45) | 0.84  (0.18, 3.76) |
| **0.43  (0.21, 0.9)** | 0.58  (0.29, 1.16) | **0.26  (0.1, 0.7)** | 0.77  (0.33, 1.75) | 0.67  (0.25, 1.83) | DTG+ TDF+XTC | 1  (0.4, 2.53) | 2.11  (0.26, 18.73) | 0.72  (0.14, 3.36) |
| **0.47  (0.29, 0.75)** | 0.63  (0.34, 1.17) | **0.28  (0.13, 0.64)** | 0.83  (0.45, 1.54) | 0.72  (0.31, 1.68) | 1.08  (0.52, 2.22) | DTG+ ABC+3TC | 2.1  (0.33, 15.41) | 0.72  (0.19, 2.46) |
| **0.35  (0.12, 0.99)** | 0.47  (0.15, 1.44) | **0.21  (0.06, 0.72)** | 0.61  (0.2, 1.88) | 0.53  (0.15, 1.88) | 0.8  (0.24, 2.6) | 0.74  (0.29, 1.89) | DTG+ TAF+FTC | 0.35  (0.07, 1.28) |
| 0.48  (0.2, 1.12) | 0.64  (0.25, 1.64) | **0.29  (0.1, 0.84)** | 0.84  (0.33, 2.15) | 0.73  (0.25, 2.21) | 1.1  (0.4, 2.99) | 1.02  (0.5, 2.05) | 1.37  (0.74, 2.57) | BIC+ TAF+FTC |

Data are odds ratios (95% credible interval) of the antiretroviral regimen column versus the antiretroviral regimen row. Different antiretroviral regimens are on the diagonal. The values below the diagonal are the results of the low viral load group and those above the diagonal are the results of the high viral load group. Values in bold indicate statistically significant comparisons.

ABC, abacavir; BIC, bictegravir; DTG, dolutegravir; EFV, efavirenz; EFV400, 400mg efavirenz; EVG/c, cobicistat-boosted elvitegravir; FTC, emtricitabine; RAL, raltegravir; RPV, rilpivirine; TAF, tenofovir alafenamide; TDF, tenofovir disoproxil fumarate; XTC, FTC/3TC; 3TC, lamivudine.

**Supplementary Table S22.** Relative results of antiretroviral regimens for the 96-week subgroup analysis.

| EFV+  TDF+FTC | 1.07  (0.54, 2.11) | 0.8  (0.46, 1.39) | 0.87  (0.64, 1.19) | 0.91  (0.46, 1.78) | 0.67  (0.08, 3.59) | 0.96  (0.56, 1.64) | 1.18  (0.25, 5.7) | 0.79  (0.24, 2.51) |
| --- | --- | --- | --- | --- | --- | --- | --- | --- |
| 0.8  (0.49, 1.31) | EFV400+  TDF+FTC | 0.75  (0.31, 1.79) | 0.82  (0.39, 1.73) | 0.85  (0.33, 2.22) | 0.63  (0.07, 3.87) | 0.9  (0.38, 2.13) | 1.11  (0.2, 6.12) | 0.74  (0.19, 2.82) |
| 1.48  (0.82, 2.7) | 1.85  (0.86, 4.02) | EFV+  ABC+3TC | 1.1  (0.58, 2.06) | 1.14  (0.48, 2.73) | 0.84  (0.1, 4.97) | 1.21  (0.56, 2.6) | 1.49  (0.29, 7.82) | 0.99  (0.27, 3.56) |
| **0.74  (0.55, 0.99)** | 0.92  (0.52, 1.65) | **0.5  (0.26, 0.97)** | RPV+  TDF+FTC | 1.04  (0.5, 2.18) | 0.77  (0.09, 4.25) | 1.1  (0.59, 2.04) | 1.36  (0.28, 6.71) | 0.9  (0.27, 3) |
| 0.71  (0.43, 1.16) | 0.89  (0.44, 1.78) | 0.48  (0.22, 1.03) | 0.96  (0.54, 1.71) | EVG/c+  TDF+FTC | 0.74  (0.08, 4.53) | 1.06  (0.45, 2.49) | 1.3  (0.24, 7.15) | 0.86  (0.22, 3.3) |
| - | - | - | - | - | DTG+  TDF+FTC | 1.42  (0.29, 10.93) | 1.79  (0.2, 21.38) | 1.18  (0.17, 11.37) |
| **0.48  (0.31, 0.72)** | 0.6  (0.31, 1.14) | **0.32  (0.16, 0.66)** | 0.65  (0.39, 1.08) | 0.67  (0.35, 1.29) | - | DTG+  ABC+3TC | 1.23  (0.29, 5.4) | 0.82  (0.28, 2.3) |
| 0.5  (0.21, 1.19) | 0.63  (0.23, 1.7) | **0.34  (0.12, 0.97)** | 0.68  (0.27, 1.7) | 0.71  (0.26, 1.92) | - | 1.05  (0.49, 2.25) | DTG+  TAF+FTC | 0.67  (0.23, 1.81) |
| 0.58  (0.28, 1.18) | 0.72  (0.3, 1.72) | **0.39  (0.15, 0.99)** | 0.78  (0.36, 1.69) | 0.81  (0.34, 1.94) | - | 1.21  (0.68, 2.16) | 1.15  (0.7, 1.88) | BIC+  TAF+FTC |

Data are odds ratios (95% credible interval) of the antiretroviral regimen column versus the antiretroviral regimen row. Different antiretroviral regimens are on the diagonal. The values below the diagonal are the results of the low viral load group and those above the diagonal are the results of the high viral load group. Values in bold indicate statistically significant comparisons.

ABC, abacavir; BIC, bictegravir; DTG, dolutegravir; EFV, efavirenz; EFV400, 400mg efavirenz; EVG/c, cobicistat-boosted elvitegravir; FTC, emtricitabine; RAL, raltegravir; RPV, rilpivirine; TAF, tenofovir alafenamide; TDF, tenofovir disoproxil fumarate; XTC, FTC/3TC; 3TC, lamivudine.

**Supplementary Table S23.** Possibility of each treatment in each rank and the SUCRA value for each treatment for the subgroup analysis at 48 weeks. the group of subjects with low baseline viral loads

| Treatment | Rank 1 | Rank 2 | Rank 3 | Rank 4 | Rank 5 | Rank 6 | Rank 7 | Rank 8 | Rank 9 | SUCRA |
| --- | --- | --- | --- | --- | --- | --- | --- | --- | --- | --- |
| EFV+TDF+FTC | 0.00 | 0.00 | 0.00 | 0.05 | 0.49 | 3.36 | 20.22 | 71.26 | 4.63 | 0.15 |
| EFV400+TDF+XTC | 0.24 | 1.31 | 3.09 | 6.12 | 14.08 | 28.15 | 36.62 | 9.09 | 1.31 | 0.35 |
| EFV+ABC+3TC | 0.00 | 0.01 | 0.04 | 0.08 | 0.24 | 0.71 | 2.22 | 5.54 | 91.17 | 0.02 |
| RPV+TDF+FTC | 4.77 | 9.24 | 12.95 | 17.54 | 27.27 | 20.01 | 7.96 | 0.25 | 0.02 | 0.57 |
| RAL+TDF+FTC | 5.41 | 6.97 | 8.31 | 10.57 | 16.39 | 21.46 | 20.36 | 8.75 | 1.77 | 0.47 |
| DTG+TDF+XTC | 25.48 | 19.66 | 17.23 | 15.99 | 11.00 | 7.34 | 2.40 | 0.77 | 0.13 | 0.75 |
| DTG+ABC+3TC | 6.88 | 18.43 | 30.03 | 26.63 | 12.34 | 4.58 | 1.07 | 0.03 | 0.00 | 0.70 |
| DTG+TAF+FTC | 52.70 | 16.96 | 9.53 | 7.14 | 5.46 | 4.07 | 2.44 | 1.33 | 0.36 | 0.84 |
| BIC+TAF+FTC | 4.51 | 27.41 | 18.82 | 15.89 | 12.73 | 10.32 | 6.72 | 2.98 | 0.62 | 0.65 |

Units of values except SUCRA values: %

ABC, abacavir; BIC, bictegravir; DTG, dolutegravir; EFV, efavirenz; EFV400, 400mg efavirenz; EVG/c, cobicistat-boosted elvitegravir; FTC, emtricitabine; RAL, raltegravir; RPV, rilpivirine; TAF, tenofovir alafenamide; TDF, tenofovir disoproxil fumarate; 3TC, lamivudine.

**Supplementary Table S24.** Possibility of each treatment in each rank and the SUCRA value for each treatment for the subgroup analysis at 48 weeks. the group of subjects with high baseline viral loads

| Treatment | Rank 1 | Rank 2 | Rank 3 | Rank 4 | Rank 5 | Rank 6 | Rank 7 | Rank 8 | Rank 9 | SUCRA |
| --- | --- | --- | --- | --- | --- | --- | --- | --- | --- | --- |
| EFV+TDF+FTC | 0.07 | 0.76 | 3.51 | 10.55 | 22.49 | 31.54 | 28.18 | 2.87 | 0.04 | 0.40 |
| EFV400+TDF+XTC | 1.19 | 8.48 | 16.36 | 19.47 | 20.71 | 15.85 | 10.82 | 5.66 | 1.46 | 0.53 |
| EFV+ABC+3TC | 0.01 | 0.07 | 0.21 | 0.50 | 1.10 | 2.87 | 8.46 | 22.02 | 64.76 | 0.07 |
| RPV+TDF+FTC | 0.04 | 0.21 | 0.70 | 1.86 | 4.37 | 11.15 | 22.85 | 45.20 | 13.62 | 0.20 |
| RAL+TDF+FTC | 6.80 | 14.45 | 16.21 | 18.17 | 15.55 | 14.43 | 9.01 | 4.43 | 0.95 | 0.59 |
| DTG+TDF+XTC | 13.91 | 24.40 | 20.21 | 17.38 | 11.30 | 6.47 | 3.95 | 1.86 | 0.52 | 0.71 |
| DTG+ABC+3TC | 8.04 | 23.81 | 26.97 | 19.20 | 12.66 | 6.23 | 2.33 | 0.67 | 0.09 | 0.70 |
| DTG+TAF+FTC | 68.78 | 7.69 | 4.92 | 3.83 | 2.99 | 3.24 | 3.37 | 3.47 | 1.71 | 0.86 |
| BIC+TAF+FTC | 1.15 | 20.13 | 10.91 | 9.05 | 8.84 | 8.21 | 11.04 | 13.82 | 16.85 | 0.45 |

Units of values except SUCRA values: %

ABC, abacavir; BIC, bictegravir; DTG, dolutegravir; EFV, efavirenz; EFV400, 400mg efavirenz; EVG/c, cobicistat-boosted elvitegravir; FTC, emtricitabine; RAL, raltegravir; RPV, rilpivirine; TAF, tenofovir alafenamide; TDF, tenofovir disoproxil fumarate; 3TC, lamivudine.

**Supplementary Table S25.** Possibility of each treatment in each rank and the SUCRA value for each treatment for the subgroup analysis at 96 weeks. the group of subjects with low baseline viral loads

| Treatment | Rank 1 | Rank 2 | Rank 3 | Rank 4 | Rank 5 | Rank 6 | Rank 7 | Rank 8 | SUCRA |
| --- | --- | --- | --- | --- | --- | --- | --- | --- | --- |
| EFV+TDF+FTC | 0.00 | 0.00 | 0.07 | 0.93 | 5.42 | 25.07 | 61.95 | 6.56 | 0.19 |
| EFV400+TDF+FTC | 3.00 | 5.93 | 8.23 | 17.92 | 21.77 | 24.78 | 14.60 | 3.77 | 0.43 |
| EFV+ABC+3TC | 0.03 | 0.13 | 0.27 | 0.77 | 1.69 | 3.80 | 7.61 | 85.71 | 0.04 |
| RPV+TDF+FTC | 1.73 | 6.74 | 11.35 | 26.61 | 32.06 | 19.09 | 2.17 | 0.25 | 0.50 |
| EVG/c+TDF+FTC | 6.96 | 10.62 | 11.85 | 25.63 | 21.37 | 15.67 | 6.48 | 1.43 | 0.54 |
| DTG+ABC+3TC | 43.11 | 28.22 | 23.39 | 4.20 | 0.90 | 0.18 | 0.01 | 0.00 | 0.87 |
| DTG+TAF+FTC | 38.19 | 21.59 | 15.58 | 9.12 | 6.45 | 4.45 | 3.38 | 1.24 | 0.78 |
| BIC+TAF+FTC | 6.98 | 26.78 | 29.26 | 14.81 | 10.35 | 6.96 | 3.82 | 1.05 | 0.67 |

Units of values except SUCRA values: %

ABC, abacavir; BIC, bictegravir; DTG, dolutegravir; EFV, efavirenz; EFV400, 400mg efavirenz; EVG/c, cobicistat-boosted elvitegravir; FTC, emtricitabine; RAL, raltegravir; RPV, rilpivirine; TAF, tenofovir alafenamide; TDF, tenofovir disoproxil fumarate; 3TC, lamivudine.

**Supplementary Table S26.** Possibility of each treatment in each rank and the SUCRA value for each treatment for the subgroup analysis at 96 weeks. the group of subjects with high baseline viral loads

| Treatment | Rank 1 | Rank 2 | Rank 3 | Rank 4 | Rank 5 | Rank 6 | Rank 7 | Rank 8 | Rank 9 | SUCRA |
| --- | --- | --- | --- | --- | --- | --- | --- | --- | --- | --- |
| EFV+TDF+FTC | 3.48 | 12.84 | 21.59 | 23.71 | 19.91 | 12.46 | 4.90 | 1.02 | 0.08 | 0.62 |
| EFV400+TDF+FTC | 19.84 | 17.49 | 13.48 | 10.83 | 9.72 | 8.92 | 8.32 | 7.18 | 4.21 | 0.63 |
| EFV+ABC+3TC | 3.28 | 5.82 | 7.20 | 8.81 | 11.32 | 14.33 | 16.81 | 18.92 | 13.51 | 0.37 |
| RPV+TDF+FTC | 1.75 | 4.98 | 9.12 | 13.73 | 17.39 | 19.11 | 17.60 | 12.00 | 4.32 | 0.43 |
| EVG/c+TDF+FTC | 10.01 | 11.90 | 10.93 | 10.47 | 10.84 | 11.57 | 12.46 | 12.95 | 8.86 | 0.50 |
| DTG+TDF+FTC | 15.78 | 8.37 | 5.52 | 4.19 | 3.98 | 4.69 | 7.27 | 9.35 | 40.85 | 0.37 |
| DTG+ABC+3TC | 4.83 | 11.35 | 16.34 | 16.81 | 15.82 | 15.51 | 11.61 | 6.23 | 1.50 | 0.55 |
| DTG+TAF+FTC | 37.66 | 12.74 | 6.33 | 4.73 | 4.75 | 5.62 | 8.73 | 11.27 | 8.17 | 0.65 |
| BIC+TAF+FTC | 3.36 | 14.51 | 9.49 | 6.72 | 6.26 | 7.79 | 12.31 | 21.07 | 18.49 | 0.39 |

Units of values except SUCRA values: %

ABC, abacavir; BIC, bictegravir; DTG, dolutegravir; EFV, efavirenz; EFV400, 400mg efavirenz; EVG/c, cobicistat-boosted elvitegravir; FTC, emtricitabine; RAL, raltegravir; RPV, rilpivirine; TAF, tenofovir alafenamide; TDF, tenofovir disoproxil fumarate; 3TC, lamivudine.

**Supplementary Table S27.** Summary of our confidence in relative results and ranking of treatments for virologic suppression.

| Comparison | 48 weeks | | |  | 96 weeks | | |
| --- | --- | --- | --- | --- | --- | --- | --- |
|  | **Nature of the evidence** | **Confidence** | **Downgrading due to** |  | **Nature of the evidence** | **Confidence** | **Downgrading due to** |
| EFV+TDF+FTC vs EFV400+TDF+FTC/3TC | Mixed | High | ‒ |  | Mixed | Moderate | Imprecision |
| EFV+TDF+FTC vs EFV+ABC+3TC | Mixed | Low | Study Limitations; Imprecision |  | Mixed | Low | Study Limitations; Imprecision |
| EFV+TDF+FTC vs RPV+TDF+FTC | Mixed | Moderate | Imprecision |  | Mixed | Moderate | Imprecision |
| EFV+TDF+FTC vs RPV+ABC+3TC | Mixed | Moderate | Imprecision |  | Mixed | Moderate | Imprecision |
| EFV+TDF+FTC vs RAL+TDF+FTC | Mixed | Moderate | Imprecision |  | Mixed | Moderate | Imprecision |
| EFV+TDF+FTC vs RAL+ABC+3TC | Indirect | Moderate | Imprecision |  | Indirect | Moderate | Imprecision |
| EFV+TDF+FTC vs EVG/c+TDF+FTC | Mixed | Moderate | Imprecision |  | Mixed | Moderate | Imprecision |
| EFV+TDF+FTC vs EVG/c+TAF+FTC | Indirect | Moderate | Imprecision |  | Indirect | Moderate | Imprecision |
| EFV+TDF+FTC vs DTG+TDF+FTC/3TC | Indirect | High | ‒ |  | Indirect | High | ‒ |
| EFV+TDF+FTC vs DTG+ABC+3TC | Mixed | High | ‒ |  | Mixed | High | ‒ |
| EFV+TDF+FTC vs DTG+TAF+FTC | Indirect | Moderate | Imprecision |  | Indirect | Moderate | Imprecision |
| EFV+TDF+FTC vs BIC+TAF+FTC | Indirect | Moderate | Imprecision |  | Indirect | Moderate | Imprecision |
| EFV400+TDF+FTC/3TC vs EFV+ABC+3TC | Indirect | Moderate | Study Limitations |  | Indirect | Low | Study Limitations; Imprecision |
| EFV400+TDF+FTC/3TC vs RPV+TDF+FTC | Indirect | Moderate | Imprecision |  | Indirect | Moderate | Imprecision |
| EFV400+TDF+FTC/3TC vs RPV+ABC+3TC | Indirect | Moderate | Imprecision |  | Indirect | Moderate | Imprecision |
| EFV400+TDF+FTC/3TC vs RAL+TDF+FTC | Indirect | Moderate | Imprecision |  | Indirect | Moderate | Imprecision |
| EFV400+TDF+FTC/3TC vs RAL+ABC+3TC | Indirect | Moderate | Imprecision |  | Indirect | Moderate | Imprecision |
| EFV400+TDF+FTC/3TC vs EVG/c+TDF+FTC | Indirect | Moderate | Imprecision |  | Indirect | Moderate | Imprecision |
| EFV400+TDF+FTC/3TC vs EVG/c+TAF+FTC | Indirect | Moderate | Imprecision |  | Indirect | Moderate | Imprecision |
| EFV400+TDF+FTC/3TC vs DTG+TDF+FTC/3TC | Mixed | Moderate | Study Limitations |  | Indirect | High | ‒ |
| EFV400+TDF+FTC/3TC vs DTG+ABC+3TC | Indirect | Moderate | Imprecision |  | Indirect | Moderate | Imprecision |
| EFV400+TDF+FTC/3TC vs DTG+TAF+FTC | Indirect | Moderate | Imprecision |  | Indirect | Moderate | Imprecision |
| EFV400+TDF+FTC/3TC vs BIC+TAF+FTC | Indirect | Moderate | Imprecision |  | Indirect | Moderate | Imprecision |
| EFV+ABC+3TC vs RPV+TDF+FTC | Mixed | Moderate | Study Limitations |  | Mixed | Moderate | Study Limitations |
| EFV+ABC+3TC vs RPV+ABC+3TC | Mixed | Low | Study Limitations; Imprecision |  | Mixed | Low | Study Limitations; Imprecision |
| EFV+ABC+3TC vs RAL+TDF+FTC | Indirect | Moderate | Study Limitations |  | Indirect | Moderate | Study Limitations |
| EFV+ABC+3TC vs RAL+ABC+3TC | Indirect | Moderate | Study Limitations |  | Indirect | Moderate | Imprecision |
| EFV+ABC+3TC vs EVG/c+TDF+FTC | Indirect | Moderate | Study Limitations |  | Indirect | Low | Study Limitations; Imprecision |
| EFV+ABC+3TC vs EVG/c+TAF+FTC | Indirect | Moderate | Study Limitations |  | Indirect | Low | Study Limitations; Imprecision |
| EFV+ABC+3TC vs DTG+TDF+FTC/3TC | Indirect | Moderate | Study Limitations |  | Indirect | High | ‒ |
| EFV+ABC+3TC vs DTG+ABC+3TC | Indirect | Moderate | Study Limitations |  | Indirect | Moderate | Study Limitations |
| EFV+ABC+3TC vs DTG+TAF+FTC | Indirect | High | ‒ |  | Indirect | Moderate | Imprecision |
| EFV+ABC+3TC vs BIC+TAF+FTC | Indirect | Moderate | Study Limitations |  | Indirect | Moderate | Imprecision |
| RPV+TDF+FTC vs RPV+ABC+3TC | Mixed | Moderate | Imprecision |  | Mixed | Moderate | Imprecision |
| RPV+TDF+FTC vs RAL+TDF+FTC | Indirect | Moderate | Imprecision |  | Indirect | Moderate | Imprecision |
| RPV+TDF+FTC vs RAL+ABC+3TC | Indirect | Moderate | Imprecision |  | Indirect | Moderate | Imprecision |
| RPV+TDF+FTC vs EVG/c+TDF+FTC | Indirect | Moderate | Imprecision |  | Indirect | Moderate | Imprecision |
| RPV+TDF+FTC vs EVG/c+TAF+FTC | Indirect | Moderate | Imprecision |  | Indirect | Moderate | Imprecision |
| RPV+TDF+FTC vs DTG+TDF+FTC/3TC | Indirect | High | ‒ |  | Indirect | High | ‒ |
| RPV+TDF+FTC vs DTG+ABC+3TC | Indirect | Moderate | Imprecision |  | Indirect | Moderate | Imprecision |
| RPV+TDF+FTC vs DTG+TAF+FTC | Indirect | Moderate | Imprecision |  | Indirect | Moderate | Imprecision |
| RPV+TDF+FTC vs BIC+TAF+FTC | Indirect | Moderate | Imprecision |  | Indirect | Moderate | Imprecision |
| RPV+ABC+3TC vs RAL+TDF+FTC | Indirect | Moderate | Imprecision |  | Indirect | Moderate | Imprecision |
| RPV+ABC+3TC vs RAL+ABC+3TC | Indirect | Moderate | Imprecision |  | Indirect | Moderate | Imprecision |
| RPV+ABC+3TC vs EVG/c+TDF+FTC | Indirect | Moderate | Imprecision |  | Indirect | Moderate | Imprecision |
| RPV+ABC+3TC vs EVG/c+TAF+FTC | Indirect | Moderate | Imprecision |  | Indirect | Moderate | Imprecision |
| RPV+ABC+3TC vs DTG+TDF+FTC/3TC | Indirect | Moderate | Imprecision |  | Indirect | High | ‒ |
| RPV+ABC+3TC vs DTG+ABC+3TC | Indirect | Moderate | Imprecision |  | Indirect | Moderate | Imprecision |
| RPV+ABC+3TC vs DTG+TAF+FTC | Indirect | Moderate | Imprecision |  | Indirect | Moderate | Imprecision |
| RPV+ABC+3TC vs BIC+TAF+FTC | Indirect | Moderate | Imprecision |  | Indirect | Moderate | Imprecision |
| RAL+TDF+FTC vs RAL+ABC+3TC | Mixed | Moderate | Imprecision |  | Mixed | Moderate | Imprecision |
| RAL+TDF+FTC vs EVG/c+TDF+FTC | Indirect | Moderate | Imprecision |  | Indirect | Moderate | Imprecision |
| RAL+TDF+FTC vs EVG/c+TAF+FTC | Indirect | Moderate | Imprecision |  | Indirect | Moderate | Imprecision |
| RAL+TDF+FTC vs DTG+TDF+FTC/3TC | Mixed | Moderate | Imprecision |  | Mixed | High | ‒ |
| RAL+TDF+FTC vs DTG+ABC+3TC | Mixed | Moderate | Imprecision |  | Mixed | Moderate | Imprecision |
| RAL+TDF+FTC vs DTG+TAF+FTC | Indirect | Moderate | Imprecision |  | Indirect | Moderate | Imprecision |
| RAL+TDF+FTC vs BIC+TAF+FTC | Indirect | Moderate | Imprecision |  | Indirect | Moderate | Imprecision |
| RAL+ABC+3TC vs EVG/c+TDF+FTC | Indirect | Moderate | Imprecision |  | Indirect | Moderate | Imprecision |
| RAL+ABC+3TC vs EVG/c+TAF+FTC | Indirect | Moderate | Imprecision |  | Indirect | Moderate | Imprecision |
| RAL+ABC+3TC vs DTG+TDF+FTC/3TC | Mixed | Moderate | Imprecision |  | Mixed | High | ‒ |
| RAL+ABC+3TC vs DTG+ABC+3TC | Mixed | Moderate | Imprecision |  | Mixed | Moderate | Imprecision |
| RAL+ABC+3TC vs DTG+TAF+FTC | Indirect | Moderate | Imprecision |  | Indirect | Moderate | Imprecision |
| RAL+ABC+3TC vs BIC+TAF+FTC | Indirect | Moderate | Imprecision |  | Indirect | Moderate | Imprecision |
| EVG/c+TDF+FTC vs EVG/c+TAF+FTC | Mixed | Moderate | Imprecision |  | Mixed | Moderate | Imprecision |
| EVG/c+TDF+FTC vs DTG+TDF+FTC/3TC | Indirect | Moderate | Imprecision |  | Indirect | High | ‒ |
| EVG/c+TDF+FTC vs DTG+ABC+3TC | Indirect | Moderate | Imprecision |  | Indirect | Moderate | Imprecision |
| EVG/c+TDF+FTC vs DTG+TAF+FTC | Indirect | Moderate | Imprecision |  | Indirect | Moderate | Imprecision |
| EVG/c+TDF+FTC vs BIC+TAF+FTC | Indirect | Moderate | Imprecision |  | Indirect | Moderate | Imprecision |
| EVG/c+TAF+FTC vs DTG+TDF+FTC/3TC | Indirect | Moderate | Imprecision |  | Indirect | Moderate | Imprecision |
| EVG/c+TAF+FTC vs DTG+ABC+3TC | Indirect | Moderate | Imprecision |  | Indirect | Moderate | Imprecision |
| EVG/c+TAF+FTC vs DTG+TAF+FTC | Indirect | Moderate | Imprecision |  | Indirect | Moderate | Imprecision |
| EVG/c+TAF+FTC vs BIC+TAF+FTC | Indirect | Moderate | Imprecision |  | Indirect | Moderate | Imprecision |
| DTG+TDF+FTC/3TC vs DTG+ABC+3TC | Mixed | Moderate | Imprecision |  | Mixed | High | ‒ |
| DTG+TDF+FTC/3TC vs DTG+TAF+FTC | Indirect | Moderate | Imprecision |  | Indirect | Moderate | Imprecision |
| DTG+TDF+FTC/3TC vs BIC+TAF+FTC | Indirect | Moderate | Imprecision |  | Indirect | High | ‒ |
| DTG+ABC+3TC vs DTG+TAF+FTC | Indirect | Moderate | Imprecision |  | Indirect | Moderate | Imprecision |
| DTG+ABC+3TC vs BIC+TAF+FTC | Mixed | Moderate | Imprecision |  | Mixed | Moderate | Imprecision |
| DTG+TAF+FTC vs BIC+TAF+FTC | Mixed | Moderate | Imprecision |  | Mixed | Moderate | Imprecision |
| Ranking of treatments | ‒ | Moderate | Publication bias |  | ‒ | Moderate | Publication bias |

ABC, abacavir; BIC, bictegravir; DTG, dolutegravir; EFV, efavirenz; EFV400, 400mg efavirenz; EVG/c, cobicistat-boosted elvitegravir; FTC, emtricitabine; RAL, raltegravir; RPV, rilpivirine; TAF, tenofovir alafenamide; TDF, tenofovir disoproxil fumarate; vs, versus; 3TC, lamivudine.

**Supplementary Table S28.** Summary of our confidence in relative results and ranking of treatments for CD4^+^ cell recovery.

| Comparison | 48 weeks | | |  | 96 weeks | | |
| --- | --- | --- | --- | --- | --- | --- | --- |
|  | **Nature of the evidence** | **Confidence** | **Downgrading due to** |  | **Nature of the evidence** | **Confidence** | **Downgrading due to** |
| EFV+TDF+FTC vs EFV400+TDF+FTC/3TC | Mixed | High | ‒ |  | Mixed | High |  |
| EFV+TDF+FTC vs EFV+ABC+3TC | Mixed | Low | Study Limitations; Imprecision |  | Mixed | Low | Study Limitations; Imprecision |
| EFV+TDF+FTC vs RPV+TDF+FTC | Mixed | Low | Study Limitations; Imprecision |  | Mixed | Low | Study Limitations; Imprecision |
| EFV+TDF+FTC vs RAL+TDF+FTC | Mixed | High | ‒ |  | Mixed | Moderate | Imprecision |
| EFV+TDF+FTC vs EVG/c+TDF+FTC | Mixed | High | ‒ |  | Mixed | Moderate | Imprecision |
| EFV+TDF+FTC vs EVG/c+TAF+FTC | Indirect | High | ‒ |  | Indirect | Moderate | Imprecision |
| EFV+TDF+FTC vs DTG+TDF+3TC | Indirect | Moderate | Study Limitations |  | ‒ | ‒ | ‒ |
| EFV+TDF+FTC vs DTG+ABC+3TC | Mixed | High | ‒ |  | Mixed | High | ‒ |
| EFV+TDF+FTC vs DTG+TAF+FTC | Indirect | High | ‒ |  | Indirect | High | ‒ |
| EFV+TDF+FTC vs BIC+TAF+FTC | Indirect | High | ‒ |  | Indirect | Moderate | Imprecision |
| EFV400+TDF+FTC/3TC vs EFV+ABC+3TC | Indirect | Low | Study Limitations; Imprecision |  | Indirect | Low | Study Limitations; Imprecision |
| EFV400+TDF+FTC/3TC vs RPV+TDF+FTC | Indirect | Low | Study Limitations; Imprecision |  | Indirect | Low | Study Limitations; Imprecision |
| EFV400+TDF+FTC/3TC vs RAL+TDF+FTC | Indirect | Moderate | Imprecision |  | Indirect | Moderate | Imprecision |
| EFV400+TDF+FTC/3TC vs EVG/c+TDF+FTC | Indirect | Moderate | Imprecision |  | Indirect | Moderate | Imprecision |
| EFV400+TDF+FTC/3TC vs EVG/c+TAF+FTC | Indirect | Moderate | Imprecision |  | Indirect | Moderate | Imprecision |
| EFV400+TDF+FTC/3TC vs DTG+TDF+3TC | Mixed | Low | Study Limitations; Indirectness |  | ‒ | ‒ | ‒ |
| EFV400+TDF+FTC/3TC vs DTG+ABC+3TC | Indirect | High | ‒ |  | Indirect | Moderate | Imprecision |
| EFV400+TDF+FTC/3TC vs DTG+TAF+FTC | Indirect | High | ‒‒ |  | Indirect | Moderate | Imprecision |
| EFV400+TDF+FTC/3TC vs BIC+TAF+FTC | Indirect | Moderate | Imprecision |  | Indirect | Moderate | Imprecision |
| EFV+ABC+3TC vs RPV+TDF+FTC | Indirect | Low | Study Limitations; Imprecision |  | Indirect | Low | Study Limitations; Imprecision |
| EFV+ABC+3TC vs RAL+TDF+FTC | Indirect | Low | Study Limitations; Imprecision |  | Indirect | Low | Study Limitations; Imprecision |
| EFV+ABC+3TC vs EVG/c+TDF+FTC | Indirect | Low | Study Limitations; Imprecision |  | Indirect | Low | Study Limitations; Imprecision |
| EFV+ABC+3TC vs EVG/c+TAF+FTC | Indirect | Low | Study Limitations; Imprecision |  | Indirect | Low | Study Limitations; Imprecision |
| EFV+ABC+3TC vs DTG+TDF+3TC | Indirect | Low | Study Limitations; Imprecision |  | ‒ | ‒ | ‒ |
| EFV+ABC+3TC vs DTG+ABC+3TC | Indirect | Low | Study Limitations; Imprecision |  | Indirect | Low | Study Limitations; Imprecision |
| EFV+ABC+3TC vs DTG+TAF+FTC | Indirect | Moderate | Study Limitations |  | Indirect | Low | Study Limitations; Imprecision |
| EFV+ABC+3TC vs BIC+TAF+FTC | Indirect | Low | Study Limitations; Imprecision |  | Indirect | Low | Study Limitations; Imprecision |
| RPV+TDF+FTC vs RAL+TDF+FTC | Indirect | Low | Study Limitations; Imprecision |  | Indirect | Low | Study Limitations; Imprecision |
| RPV+TDF+FTC vs EVG/c+TDF+FTC | Indirect | Low | Study Limitations; Imprecision |  | Indirect | Low | Study Limitations; Imprecision |
| RPV+TDF+FTC vs EVG/c+TAF+FTC | Indirect | Moderate | Study Limitations |  | Indirect | Low | Study Limitations; Imprecision |
| RPV+TDF+FTC vs DTG+TDF+3TC | Indirect | Moderate | Study Limitations |  | ‒ | ‒ | ‒ |
| RPV+TDF+FTC vs DTG+ABC+3TC | Indirect | Moderate | Study Limitations |  | Indirect | Low | Study Limitations; Imprecision |
| RPV+TDF+FTC vs DTG+TAF+FTC | Indirect | Moderate | Study Limitations |  | Indirect | Moderate | Study Limitations |
| RPV+TDF+FTC vs BIC+TAF+FTC | Indirect | Moderate | Study Limitations |  | Indirect | Low | Study Limitations; Imprecision |
| RAL+TDF+FTC vs EVG/c+TDF+FTC | Indirect | Moderate | Imprecision |  | Indirect | Moderate | Imprecision |
| RAL+TDF+FTC vs EVG/c+TAF+FTC | Indirect | Moderate | Imprecision |  | Indirect | Moderate | Imprecision |
| RAL+TDF+FTC vs DTG+TDF+3TC | Indirect | Low | Study Limitations; Imprecision |  | ‒ | ‒ | ‒ |
| RAL+TDF+FTC vs DTG+ABC+3TC | Indirect | Moderate | Imprecision |  | Indirect | Moderate | Imprecision |
| RAL+TDF+FTC vs DTG+TAF+FTC | Indirect | High | ‒ |  | Indirect | Moderate | Imprecision |
| RAL+TDF+FTC vs BIC+TAF+FTC | Indirect | Moderate | Imprecision |  | Indirect | Moderate | Imprecision |
| EVG/c+TDF+FTC vs EVG/c+TAF+FTC | Mixed | High | ‒ |  | Mixed | Moderate | Imprecision |
| EVG/c+TDF+FTC vs DTG+TDF+3TC | Indirect | Low | Study Limitations; Imprecision |  | ‒ | ‒ | ‒ |
| EVG/c+TDF+FTC vs DTG+ABC+3TC | Indirect | Moderate | Imprecision |  | Indirect | Moderate | Imprecision |
| EVG/c+TDF+FTC vs DTG+TAF+FTC | Indirect | Moderate | Imprecision |  | Indirect | Moderate | Imprecision |
| EVG/c+TDF+FTC vs BIC+TAF+FTC | Indirect | Moderate | Imprecision |  | Indirect | Moderate | Imprecision |
| EVG/c+TAF+FTC vs DTG+TDF+3TC | Indirect | Low | Study Limitations; Imprecision |  | ‒ | ‒ | ‒ |
| EVG/c+TAF+FTC vs DTG+ABC+3TC | Indirect | Moderate | Imprecision |  | Indirect | Moderate | Imprecision |
| EVG/c+TAF+FTC vs DTG+TAF+FTC | Indirect | Moderate | Imprecision |  | Indirect | Moderate | Imprecision |
| EVG/c+TAF+FTC vs BIC+TAF+FTC | Indirect | Moderate | Imprecision |  | Indirect | Moderate | Imprecision |
| DTG+TDF+3TC vs DTG+ABC+3TC | Indirect | Low | Study Limitations; Imprecision |  | ‒ | ‒ | ‒ |
| DTG+TDF+3TC vs DTG+TAF+FTC | Indirect | Low | Study Limitations; Imprecision |  | ‒ | ‒ | ‒ |
| DTG+TDF+3TC vs BIC+TAF+FTC | Indirect | Low | Study Limitations; Imprecision |  | ‒ | ‒ | ‒ |
| DTG+ABC+3TC vs DTG+TAF+FTC | Indirect | Moderate | Imprecision |  | Indirect | Moderate | Imprecision |
| DTG+ABC+3TC vs BIC+TAF+FTC | Mixed | Moderate | Imprecision |  | Mixed | Moderate | Imprecision |
| DTG+TAF+FTC vs BIC+TAF+FTC | Mixed | Moderate | Imprecision |  | Mixed | High | ‒ |
| Ranking of treatments | ‒ | Moderate | Study Limitations |  |  | High | ‒ |

ABC, abacavir; BIC, bictegravir; DTG, dolutegravir; EFV, efavirenz; EFV400, 400mg efavirenz; EVG/c, cobicistat-boosted elvitegravir; FTC, emtricitabine; RAL, raltegravir; RPV, rilpivirine; TAF, tenofovir alafenamide; TDF, tenofovir disoproxil fumarate; vs, versus; 3TC, lamivudine.

**Supplementary Table S29.** Summary of our confidence in relative results and ranking of regimens for discontinuations.

| Comparison | 48 weeks | | |  | 96 weeks | | |
| --- | --- | --- | --- | --- | --- | --- | --- |
|  | **Nature of the evidence** | **Confidence** | **Downgrading due to** |  | **Nature of the evidence** | **Confidence** | **Downgrading due to** |
| EFV+TDF+FTC vs EFV400+TDF+FTC/3TC | Mixed | Moderate | Imprecision |  | Mixed | Moderate | Imprecision |
| EFV+TDF+FTC vs EFV+ABC+3TC | Mixed | Moderate | Study Limitations |  | Mixed | Low | Study Limitations; Imprecision |
| EFV+TDF+FTC vs RPV+TDF+FTC | Mixed | Low | Study Limitations; Imprecision |  | Mixed | High | ‒ |
| EFV+TDF+FTC vs RAL+TDF+FTC | Mixed | Moderate | Imprecision |  | Mixed | Moderate | Imprecision |
| EFV+TDF+FTC vs EVG/c+TDF+FTC | Mixed | Moderate | Imprecision |  | Mixed | Moderate | Imprecision |
| EFV+TDF+FTC vs EVG/c+TAF+FTC | Indirect | High | ‒ |  | Indirect | Moderate | Imprecision |
| EFV+TDF+FTC vs DTG+TDF+3TC | Indirect | Very low | Study Limitations; Imprecision; Indirectness |  | ‒ | ‒ | ‒ |
| EFV+TDF+FTC vs DTG+ABC+3TC | Mixed | High | ‒ |  | Mixed | High | ‒ |
| EFV+TDF+FTC vs DTG+TAF+FTC | Indirect | Moderate | Imprecision |  | Indirect | Moderate | Imprecision |
| EFV+TDF+FTC vs BIC+TAF+FTC | Indirect | Moderate | Imprecision |  | Indirect | Moderate | Imprecision |
| EFV400+TDF+FTC/3TC vs EFV+ABC+3TC | Indirect | Moderate | Study Limitations |  | Indirect | Moderate | Study Limitations |
| EFV400+TDF+FTC/3TC vs RPV+TDF+FTC | Indirect | Low | Study Limitations; Imprecision |  | Indirect | Moderate | Imprecision |
| EFV400+TDF+FTC/3TC vs RAL+TDF+FTC | Indirect | Moderate | Imprecision |  | Indirect | Moderate | Imprecision |
| EFV400+TDF+FTC/3TC vs EVG/c+TDF+FTC | Indirect | Moderate | Imprecision |  | Indirect | Moderate | Imprecision |
| EFV400+TDF+FTC/3TC vs EVG/c+TAF+FTC | Indirect | Moderate | Imprecision |  | Indirect | Moderate | Imprecision |
| EFV400+TDF+FTC/3TC vs DTG+TDF+3TC | Mixed | Very low | Study Limitations; Imprecision; Indirectness |  | ‒ | ‒ | ‒ |
| EFV400+TDF+FTC/3TC vs DTG+ABC+3TC | Indirect | Moderate | Imprecision |  | Indirect | Moderate | Imprecision |
| EFV400+TDF+FTC/3TC vs DTG+TAF+FTC | Indirect | Moderate | Imprecision |  | Indirect | Moderate | Imprecision |
| EFV400+TDF+FTC/3TC vs BIC+TAF+FTC | Indirect | Moderate | Imprecision |  | Indirect | Moderate | Imprecision |
| EFV+ABC+3TC vs RPV+TDF+FTC | Indirect | Moderate | Study Limitations |  | Indirect | Moderate | Study Limitations |
| EFV+ABC+3TC vs RAL+TDF+FTC | Indirect | Moderate | Study Limitations |  | Indirect | Moderate | Study Limitations |
| EFV+ABC+3TC vs EVG/c+TDF+FTC | Indirect | Moderate | Study Limitations |  | Indirect | Low | Study Limitations; Imprecision |
| EFV+ABC+3TC vs EVG/c+TAF+FTC | Indirect | Moderate | Study Limitations |  | Indirect | Moderate | Study Limitations |
| EFV+ABC+3TC vs DTG+TDF+3TC | Indirect | Moderate | Study Limitations |  | ‒ | ‒ | ‒ |
| EFV+ABC+3TC vs DTG+ABC+3TC | Indirect | High | ‒ |  | Indirect | Moderate | Study Limitations |
| EFV+ABC+3TC vs DTG+TAF+FTC | Indirect | High | ‒ |  | Indirect | Moderate | Study Limitations |
| EFV+ABC+3TC vs BIC+TAF+FTC | Indirect | Low | Study Limitations; Imprecision |  | Indirect | Moderate | Study Limitations |
| RPV+TDF+FTC vs RAL+TDF+FTC | Indirect | Low | Study Limitations; Imprecision |  | Indirect | Moderate | Imprecision |
| RPV+TDF+FTC vs EVG/c+TDF+FTC | Indirect | Low | Study Limitations; Imprecision |  | Indirect | Moderate | Imprecision |
| RPV+TDF+FTC vs EVG/c+TAF+FTC | Indirect | Low | Study Limitations; Imprecision |  | Indirect | Moderate | Imprecision |
| RPV+TDF+FTC vs DTG+TDF+3TC | Indirect | Low | Study Limitations; Imprecision |  | ‒ | ‒ | ‒ |
| RPV+TDF+FTC vs DTG+ABC+3TC | Indirect | Low | Study Limitations; Imprecision |  | Indirect | Moderate | Imprecision |
| RPV+TDF+FTC vs DTG+TAF+FTC | Indirect | Low | Study Limitations; Imprecision |  | Indirect | Moderate | Imprecision |
| RPV+TDF+FTC vs BIC+TAF+FTC | Indirect | Low | Study Limitations; Imprecision |  | Indirect | Moderate | Imprecision |
| RAL+TDF+FTC vs EVG/c+TDF+FTC | Indirect | Moderate | Imprecision |  | Indirect | Moderate | Imprecision |
| RAL+TDF+FTC vs EVG/c+TAF+FTC | Indirect | Moderate | Imprecision |  | Indirect | Moderate | Imprecision |
| RAL+TDF+FTC vs DTG+TDF+3TC | Indirect | Low | Study Limitations; Imprecision |  | ‒ | ‒ | ‒ |
| RAL+TDF+FTC vs DTG+ABC+3TC | Indirect | Moderate | Imprecision |  | Indirect | Moderate | Imprecision |
| RAL+TDF+FTC vs DTG+TAF+FTC | Indirect | Moderate | Imprecision |  | Indirect | Moderate | Imprecision |
| RAL+TDF+FTC vs BIC+TAF+FTC | Indirect | Moderate | Imprecision |  | Indirect | Moderate | Imprecision |
| EVG/c+TDF+FTC vs EVG/c+TAF+FTC | Mixed | Moderate | Study Limitations |  | Mixed | Moderate | Imprecision |
| EVG/c+TDF+FTC vs DTG+TDF+3TC | Indirect | Moderate | Imprecision |  | ‒ | ‒ | ‒ |
| EVG/c+TDF+FTC vs DTG+ABC+3TC | Indirect | Moderate | Imprecision |  | Indirect | Moderate | Imprecision |
| EVG/c+TDF+FTC vs DTG+TAF+FTC | Indirect | Moderate | Imprecision |  | Indirect | Moderate | Imprecision |
| EVG/c+TDF+FTC vs BIC+TAF+FTC | Indirect | Moderate | Imprecision |  | Indirect | Moderate | Imprecision |
| EVG/c+TAF+FTC vs DTG+TDF+3TC | Indirect | Moderate | Imprecision |  | ‒ | ‒ | ‒ |
| EVG/c+TAF+FTC vs DTG+ABC+3TC | Indirect | Moderate | Study Limitations |  | Indirect | Moderate | Imprecision |
| EVG/c+TAF+FTC vs DTG+TAF+FTC | Indirect | Moderate | Imprecision |  | Indirect | Moderate | Imprecision |
| EVG/c+TAF+FTC vs BIC+TAF+FTC | Indirect | Moderate | Imprecision |  | Indirect | Moderate | Imprecision |
| DTG+TDF+3TC vs DTG+ABC+3TC | Indirect | Moderate | Study Limitations |  | ‒ | ‒ | ‒ |
| DTG+TDF+3TC vs DTG+TAF+FTC | Indirect | Moderate | Study Limitations |  | ‒ | ‒ | ‒ |
| DTG+TDF+3TC vs BIC+TAF+FTC | Indirect | Moderate | Study Limitations |  | ‒ | ‒ | ‒ |
| DTG+ABC+3TC vs DTG+TAF+FTC | Indirect | Moderate | Study Limitations |  | Indirect | Moderate | Imprecision |
| DTG+ABC+3TC vs BIC+TAF+FTC | Mixed | Moderate | Imprecision |  | Mixed | Moderate | Imprecision |
| DTG+TAF+FTC vs BIC+TAF+FTC | Mixed | Moderate | Imprecision |  | Mixed | Moderate | Imprecision |
| Ranking of treatments | ‒ | High | ‒ |  | ‒ | High | ‒ |

ABC, abacavir; BIC, bictegravir; DTG, dolutegravir; EFV, efavirenz; EFV400, 400mg efavirenz; EVG/c, cobicistat-boosted elvitegravir; FTC, emtricitabine; RAL, raltegravir; RPV, rilpivirine; TAF, tenofovir alafenamide; TDF, tenofovir disoproxil fumarate; vs, versus; 3TC, lamivudine.

**Supplementary Table S30.** Summary of our confidence in relative results and ranking of regimens for adverse events.

| Comparison | 48 weeks | | |  | 96 weeks | | |
| --- | --- | --- | --- | --- | --- | --- | --- |
|  | **Nature of the evidence** | **Confidence** | **Downgrading due to** |  | **Nature of the evidence** | **Confidence** | **Downgrading due to** |
| EFV+TDF+FTC vs EFV400+TDF+FTC | Mixed | Moderate | Imprecision |  | Mixed | Moderate | Imprecision |
| EFV+TDF+FTC vs EFV+ABC+3TC | ‒ | ‒ | ‒ |  | Mixed | Low | Study Limitations; Imprecision |
| EFV+TDF+FTC vs RPV+TDF+FTC | Mixed | Moderate | Study Limitations |  | Mixed | Low | Study Limitations; Imprecision |
| EFV+TDF+FTC vs RAL+TDF+FTC | Mixed | High |  |  | Mixed | Moderate | Imprecision |
| EFV+TDF+FTC vs DTG+ABC+3TC | Mixed | Moderate | Imprecision |  | ‒ | ‒ | ‒ |
| EFV+TDF+FTC vs DTG+TAF+FTC | Indirect | Moderate | ‒ |  | ‒ | ‒ | ‒ |
| EFV+TDF+FTC vs BIC+TAF+FTC | Indirect | Moderate | ‒ |  | ‒ | ‒ | ‒ |
| EFV400+TDF+FTC vs EFV+ABC+3TC | ‒ | ‒ | ‒ |  | Indirect | Low | Study Limitations; Imprecision |
| EFV400+TDF+FTC vs RPV+TDF+FTC | Indirect | Low | Study Limitations; Imprecision |  | Indirect | Moderate | Imprecision |
| EFV400+TDF+FTC vs RAL+TDF+FTC | Indirect | High |  |  | Indirect | Low | Study Limitations; Imprecision |
| EFV400+TDF+FTC vs DTG+ABC+3TC | Indirect | Moderate | Imprecision |  | ‒ | ‒ | ‒ |
| EFV400+TDF+FTC vs DTG+TAF+FTC | Indirect | Moderate | Imprecision |  | ‒ | ‒ | ‒ |
| EFV400+TDF+FTC vs BIC+TAF+FTC | Indirect | High | ‒ |  | ‒ | ‒ | ‒ |
| EFV+ABC+3TC vs RPV+TDF+FTC | ‒ | ‒ | ‒ |  | Indirect | Low | Study Limitations; Imprecision |
| EFV+ABC+3TC vs RAL+TDF+FTC | ‒ | ‒ | ‒ |  | Indirect | Low | Study Limitations; Imprecision |
| RPV+TDF+FTC vs RAL+TDF+FTC | Indirect | Low | Study Limitations; Imprecision |  | Indirect | Low | Study Limitations; Imprecision |
| RPV+TDF+FTC vs DTG+ABC+3TC | Indirect | Low | Study Limitations; Imprecision |  | ‒ | ‒ | ‒ |
| RPV+TDF+FTC vs DTG+TAF+FTC | Indirect | Low | Study Limitations; Imprecision |  | ‒ | ‒ | ‒ |
| RPV+TDF+FTC vs BIC+TAF+FTC | Indirect | Low | Study Limitations; Imprecision |  | ‒ | ‒ | ‒ |
| RAL+TDF+FTC vs DTG+ABC+3TC | Indirect | Moderate | Imprecision |  | ‒ | ‒ | ‒ |
| RAL+TDF+FTC vs DTG+TAF+FTC | Indirect | Moderate | Imprecision |  | ‒ | ‒ | ‒ |
| RAL+TDF+FTC vs BIC+TAF+FTC | Indirect | Moderate | Imprecision |  | ‒ | ‒ | ‒ |
| DTG+ABC+3TC vs DTG+TAF+FTC | Indirect | High | ‒ |  | Indirect | Moderate | Imprecision |
| DTG+ABC+3TC vs BIC+TAF+FTC | Mixed | Moderate | Imprecision |  | Mixed | Moderate | Imprecision |
| DTG+TAF+FTC vs BIC+TAF+FTC | Mixed | Moderate | Imprecision |  | Mixed | Moderate | Imprecision |
| Ranking of treatments | ‒ | Moderate | Publication bias |  | ‒ | Low^a^; Moderate | Study Limitations^a^; Publication bias |

^a^ Ranking of five regimens.

ABC, abacavir; BIC, bictegravir; DTG, dolutegravir; EFV, efavirenz; EFV400, 400mg efavirenz; FTC, emtricitabine; RAL, raltegravir; RPV, rilpivirine; TAF, tenofovir alafenamide; TDF, tenofovir disoproxil fumarate; vs, versus; 3TC, lamivudine.

**Supplementary Table S31.** Summary of our confidence in relative results and ranking of regimens for drug-related adverse events.

| Comparisons | 48 weeks | | |  | 96 weeks | | |
| --- | --- | --- | --- | --- | --- | --- | --- |
|  | **Nature of the evidence** | **Confidence** | **Downgrading due to** |  | **Nature of the evidence** | **Confidence** | **Downgrading due to** |
| EFV+TDF+FTC vs EFV400+TDF+FTC | Mixed | High | ‒ |  | Mixed | High | ‒ |
| EFV+TDF+FTC vs EFV+ABC+3TC | Mixed | Low | Study Limitations; Imprecision |  | ‒ | ‒ | ‒ |
| EFV+TDF+FTC vs RAL+TDF+FTC | Mixed | High | ‒ |  | Mixed | High | ‒ |
| EFV+TDF+FTC vs DTG+ABC+3TC | Mixed | High | ‒ |  | Mixed | High | ‒ |
| EFV+TDF+FTC vs DTG+TAF+FTC | Indirect | High | ‒ |  | Indirect | High | ‒ |
| EFV+TDF+FTC vs BIC+TAF+FTC | Indirect | High | ‒ |  | Indirect | High | ‒ |
| EFV400+TDF+FTC vs EFV+ABC+3TC | Indirect | Moderate | Study Limitations |  | ‒ | ‒ | ‒ |
| EFV400+TDF+FTC vs RAL+TDF+FTC | Indirect | High | ‒ |  | Indirect | High | ‒ |
| EFV400+TDF+FTC vs DTG+ABC+3TC | Indirect | High | ‒ |  | Indirect | High | ‒ |
| EFV400+TDF+FTC vs DTG+TAF+FTC | Indirect | High | ‒ |  | Indirect | High | ‒ |
| EFV400+TDF+FTC vs BIC+TAF+FTC | Indirect | High | ‒ |  | Indirect | High | ‒ |
| EFV+ABC+3TC vs RAL+TDF+FTC | Indirect | Moderate | Study Limitations |  | ‒ | ‒ | ‒ |
| EFV+ABC+3TC vs DTG+ABC+3TC | Indirect | Moderate | Study Limitations |  | ‒ | ‒ | ‒ |
| EFV+ABC+3TC vs DTG+TAF+FTC | Indirect | Moderate | Study Limitations |  | ‒ | ‒ | ‒ |
| EFV+ABC+3TC vs BIC+TAF+FTC | Indirect | Moderate | Study Limitations |  | ‒ | ‒ | ‒ |
| RAL+TDF+FTC vs DTG+ABC+3TC | Indirect | High | ‒ |  | Indirect | Moderate | Imprecision |
| RAL+TDF+FTC vs DTG+TAF+FTC | Indirect | Moderate | Imprecision |  | Indirect | Moderate | Imprecision |
| RAL+TDF+FTC vs BIC+TAF+FTC | Indirect | Moderate | Imprecision |  | Indirect | Moderate | Imprecision |
| DTG+ABC+3TC vs DTG+TAF+FTC | Indirect | Moderate | Imprecision |  | Indirect | Moderate | Imprecision |
| DTG+ABC+3TC vs BIC+TAF+FTC | Mixed | High | ‒ |  | Mixed | High | ‒ |
| DTG+TAF+FTC vs BIC+TAF+FTC | Mixed | High | ‒ |  | Mixed | High | ‒ |
| Ranking of treatments | ‒ | Moderate | Publication bias |  | ‒ | Moderate | Publication bias |

ABC, abacavir; BIC, bictegravir; DTG, dolutegravir; EFV, efavirenz; EFV400, 400mg efavirenz; FTC, emtricitabine; RAL, raltegravir; TAF, tenofovir alafenamide; TDF, tenofovir disoproxil fumarate; vs, versus; 3TC, lamivudine.

**Supplementary Table S32.** Summary of our confidence in relative results and ranking of regimens for serious adverse events.

| **Comparison** | **48 weeks** | | |  | **96 weeks** | | |
| --- | --- | --- | --- | --- | --- | --- | --- |
|  | **Nature of the evidence** | **Confidence** | **Downgrading due to** |  | **Nature of the evidence** | **Confidence** | **Downgrading due to** |
| EFV+TDF+FTC vs EFV400+TDF+FTC | Mixed | Moderate | Imprecision |  | Mixed | Moderate | Imprecision |
| EFV+TDF+FTC vs EFV+ABC+3TC | ‒ | ‒ | ‒ |  | Mixed | Low | Study Limitations; Imprecision |
| EFV+TDF+FTC vs RPV+TDF+FTC | Mixed | Moderate | Imprecision |  | Mixed | Moderate | Imprecision |
| EFV+TDF+FTC vs RAL+TDF+FTC | Mixed | Moderate | Imprecision |  | Mixed | Moderate | Imprecision |
| EFV+TDF+FTC vs DTG+ABC+3TC | Mixed | Moderate | Imprecision |  | Mixed | Moderate | Imprecision |
| EFV+TDF+FTC vs DTG+TAF+FTC | ‒ | ‒ | ‒ |  | Indirect | High | ‒ |
| EFV+TDF+FTC vs BIC+TAF+FTC | Indirect | Moderate | Imprecision |  | Indirect | Moderate | Imprecision |
| EFV400+TDF+FTC vs EFV+ABC+3TC | ‒ | ‒ | ‒ |  | Indirect | Moderate | Study Limitations |
| EFV400+TDF+FTC vs RPV+TDF+FTC | Indirect | Moderate | Imprecision |  | Indirect | Moderate | Imprecision |
| EFV400+TDF+FTC vs RAL+TDF+FTC | Indirect | Moderate | Imprecision |  | Indirect | Moderate | Imprecision |
| EFV400+TDF+FTC vs DTG+ABC+3TC | Indirect | Moderate | Imprecision |  | Indirect | Moderate | Imprecision |
| EFV400+TDF+FTC vs DTG+TAF+FTC | ‒ | ‒ | ‒ |  | Indirect | Moderate | Imprecision |
| EFV400+TDF+FTC vs BIC+TAF+FTC | Indirect | Moderate | Imprecision |  | Indirect | Moderate | Imprecision |
| EFV+ABC+3TC vs RPV+TDF+FTC | ‒ | ‒ | ‒ |  | Indirect | Moderate | Study Limitations |
| EFV+ABC+3TC vs RAL+TDF+FTC | ‒ | ‒ | ‒ |  | Indirect | Low | Study Limitations; Imprecision |
| EFV+ABC+3TC vs DTG+ABC+3TC | ‒ | ‒ | ‒ |  | Indirect | Low | Study Limitations; Imprecision |
| EFV+ABC+3TC vs DTG+TAF+FTC | ‒ | ‒ | ‒ |  | Indirect | Low | Study Limitations |
| EFV+ABC+3TC vs BIC+TAF+FTC | ‒ | ‒ | ‒ |  | Indirect | Low | Study Limitations; Imprecision |
| RPV+TDF+FTC vs RAL+TDF+FTC | Indirect | Moderate | Imprecision |  | Indirect | Moderate | Imprecision |
| RPV+TDF+FTC vs DTG+ABC+3TC | Indirect | Moderate | Imprecision |  | Indirect | Moderate | Imprecision |
| RPV+TDF+FTC vs DTG+TAF+FTC | ‒ | ‒ | ‒ |  | Indirect | Moderate | Imprecision |
| RPV+TDF+FTC vs BIC+TAF+FTC | Indirect | Moderate | Imprecision |  | Indirect | Moderate | Imprecision |
| RAL+TDF+FTC vs DTG+ABC+3TC | Indirect | Moderate | Imprecision |  | Indirect | Moderate | Imprecision |
| RAL+TDF+FTC vs DTG+TAF+FTC | ‒ | ‒ | ‒ |  | Indirect | High | ‒ |
| RAL+TDF+FTC vs BIC+TAF+FTC | Indirect | Moderate | Imprecision |  | Indirect | Moderate | Imprecision |
| DTG+ABC+3TC vs DTG+TAF+FTC | ‒ | ‒ | ‒ |  | Indirect | High | ‒ |
| DTG+ABC+3TC vs BIC+TAF+FTC | ‒ | ‒ | ‒ |  | Mixed | Moderate | Imprecision |
| DTG+ABC+3TC vs BIC+TAF+FTC | Mixed | Moderate | Imprecision |  | Mixed | Moderate | Imprecision |
| Ranking of treatments | ‒ | High | ‒ |  | ‒ | Moderate | Publication bias |

ABC, abacavir; BIC, bictegravir; DTG, dolutegravir; EFV, efavirenz; EFV400, 400mg efavirenz; FTC, emtricitabine; RAL, raltegravir; RPV, rilpivirine; TAF, tenofovir alafenamide; TDF, tenofovir disoproxil fumarate; vs, versus; 3TC, lamivudine

**Supplementary Table S33.** Summary of our confidence in relative results and ranking of regimens for the 48-week subgroup analysis.

| Comparison | ≤100,000 copies/mL | | |  | >100,000 copies/mL | | |
| --- | --- | --- | --- | --- | --- | --- | --- |
|  | **Nature of the evidence** | **Confidence** | **Downgrading due to** |  | **Nature of the evidence** | **Confidence** | **Downgrading due to** |
| EFV+TDF+FTC vs EFV400+TDF+FTC/3TC | Mixed | Moderate | Imprecision |  | Mixed | Moderate | Imprecision |
| EFV+TDF+FTC vs EFV+ABC+3TC | Mixed | Low | Study Limitations; Imprecision |  | Mixed | Moderate | Study Limitations |
| EFV+TDF+FTC vs RPV+TDF+FTC | Mixed | Moderate | Study Limitations |  | Mixed | Moderate | Imprecision |
| EFV+TDF+FTC vs RAL+TDF+FTC | Mixed | Moderate | Imprecision |  | Mixed | Moderate | Imprecision |
| EFV+TDF+FTC vs DTG+TDF+FTC/3TC | Indirect | Moderate | Study Limitations |  | Indirect | Low | Study Limitations; Imprecision |
| EFV+TDF+FTC vs DTG+ABC+3TC | Mixed | Moderate | Study Limitations |  | Mixed | Moderate | Imprecision |
| EFV+TDF+FTC vs DTG+TAF+FTC | Indirect | High |  |  | Indirect | Moderate | Imprecision |
| EFV+TDF+FTC vs BIC+TAF+FTC | Indirect | Moderate | Imprecision |  | Indirect | Moderate | Imprecision |
| EFV400+TDF+FTC/3TC vs RPV+TDF+FTC | Indirect | Low | Study Limitations; Imprecision |  | Indirect | Low | Study Limitations; Imprecision |
| EFV400+TDF+FTC/3TC vs RAL+TDF+FTC | Indirect | Moderate | Imprecision |  | Indirect | Moderate | Imprecision |
| EFV400+TDF+FTC/3TC vs DTG+ABC+3TC | Indirect | Low | Study Limitations; Imprecision |  | Mixed | Low | Study Limitations; Indirectness |
| EFV400+TDF+FTC/3TC vs DTG+TAF+FTC | Indirect | Moderate | Imprecision |  | Indirect | Moderate | Imprecision |
| EFV400+TDF+FTC/3TC vs EFV+ABC+3TC | Indirect | Low | Study Limitations; Imprecision |  | Indirect | Moderate | Imprecision |
| EFV400+TDF+FTC/3TC vs DTG+TDF+FTC/3TC | Mixed | Low | Study Limitations; Imprecision |  | Indirect | Low | Study Limitations; Imprecision |
| EFV400+TDF+FTC/3TC vs BIC+TAF+FTC | Indirect | Moderate | Imprecision |  | Indirect | Moderate | Imprecision |
| EFV+ABC+3TC vs RPV+TDF+FTC | Indirect | Moderate | Study Limitations |  | Indirect | Low | Study Limitations; Imprecision |
| EFV+ABC+3TC vs RAL+TDF+FTC | Indirect | Low | Study Limitations; Imprecision |  | Indirect | Low | Study Limitations; Imprecision |
| EFV+ABC+3TC vs DTG+TDF+FTC/3TC | Indirect | Moderate | Study Limitations |  | Indirect | Moderate | Study Limitations |
| EFV+ABC+3TC vs DTG+ABC+3TC | Indirect | Moderate | Study Limitations |  | Indirect | Moderate | Study Limitations |
| EFV+ABC+3TC vs DTG+TAF+FTC | Indirect | Moderate | Study Limitations |  | Indirect | Low | Study Limitations; Imprecision |
| EFV+ABC+3TC vs BIC+TAF+FTC | Indirect | Moderate | Study Limitations |  | Indirect | Low | Study Limitations; Imprecision |
| RPV+TDF+FTC vs RAL+TDF+FTC | Indirect | Low | Study Limitations; Imprecision |  | Indirect | Low | Study Limitations; Imprecision |
| RPV+TDF+FTC vs DTG+TDF+FTC/3TC | Indirect | Low | Study Limitations; Imprecision |  | Indirect | Low | Study Limitations; Imprecision |
| RPV+TDF+FTC vs DTG+ABC+3TC | Indirect | Low | Study Limitations; Imprecision |  | Indirect | Low | Study Limitations; Imprecision |
| RPV+TDF+FTC vs DTG+TAF+FTC | Indirect | Low | Study Limitations; Imprecision |  | Indirect | Low | Study Limitations; Imprecision |
| RPV+TDF+FTC vs BIC+TAF+FTC | Indirect | Low | Study Limitations; Imprecision |  | Indirect | Low | Study Limitations; Imprecision |
| RAL+TDF+FTC vs DTG+TDF+FTC/3TC | Indirect | Low | Study Limitations; Imprecision |  | Indirect | Low | Study Limitations; Imprecision |
| RAL+TDF+FTC vs DTG+ABC+3TC | Indirect | Moderate | Imprecision |  | Indirect | Moderate | Imprecision |
| RAL+TDF+FTC vs DTG+TAF+FTC | Indirect | Moderate | Imprecision |  | Indirect | Moderate | Imprecision |
| RAL+TDF+FTC vs BIC+TAF+FTC | Indirect | Moderate | Imprecision |  | Indirect | Moderate | Imprecision |
| DTG+TDF+FTC/3TC vs DTG+ABC+3TC | Mixed | Low | Study Limitations; Imprecision |  | Mixed | Low | Study Limitations; Imprecision |
| DTG+TDF+FTC/3TC vs DTG+TAF+FTC | Indirect | Low | Study Limitations; Imprecision |  | Indirect | Moderate | Imprecision |
| DTG+TDF+FTC/3TC vs BIC+TAF+FTC | Indirect | Low | Study Limitations; Imprecision |  | Indirect | Low | Study Limitations; Imprecision |
| DTG+ABC+3TC vs DTG+TAF+FTC | Indirect | Moderate | Imprecision |  | Mixed | Moderate | Imprecision |
| DTG+ABC+3TC vs BIC+TAF+FTC | Mixed | Moderate | Imprecision |  | Indirect | Moderate | Imprecision |
| DTG+TAF+FTC vs BIC+TAF+FTC | Mixed | Moderate | Imprecision |  | Mixed | Moderate | Imprecision |
| Rank of treatments | ‒ | Low | Study Limitations; Publication bias |  | ‒ | Moderate | Study Limitations |

**Supplementary Table S34.** Summary of our confidence in relative results and ranking of regimens for the 96-week subgroup analysis.

| Comparison | ≤100,000 copies/mL | | |  | >100,000 copies/mL | | |
| --- | --- | --- | --- | --- | --- | --- | --- |
|  | **Nature of the evidence** | **Confidence** | **Downgrading due to** |  | **Nature of the evidence** | **Confidence** | **Downgrading due to** |
| EFV+TDF+FTC vs EFV400+TDF+FTC | Mixed | Moderate | Imprecision |  | Mixed | Moderate | Imprecision |
| EFV+TDF+FTC vs EFV+ABC+3TC | Mixed | Low | Study Limitations; Imprecision |  | Mixed | Low | Study Limitations; Imprecision |
| EFV+TDF+FTC vs RPV+TDF+FTC | Mixed | High | ‒ |  | Mixed | Moderate | Imprecision |
| EFV+TDF+FTC vs EVG/c+TDF+FTC | Mixed | Moderate | Imprecision |  | Mixed | Moderate | Imprecision |
| EFV+TDF+FTC vs DTG+TDF+FTC | ‒ | ‒ | ‒ |  | Indirect | Low | Study Limitations; Imprecision |
| EFV+TDF+FTC vs DTG+ABC+3TC | Mixed | Moderate | Imprecision |  | Mixed | Moderate | Imprecision |
| EFV+TDF+FTC vs DTG+TAF+FTC | Mixed | Moderate | Imprecision |  | Indirect | Moderate | Imprecision |
| EFV+TDF+FTC vs BIC+TAF+FTC | Indirect | Moderate | Imprecision |  | Indirect | Moderate | Imprecision |
| EFV400+TDF+FTC vs EFV+ABC+3TC | Indirect | Low | Study Limitations; Imprecision |  | Indirect | Low | Study Limitations; Imprecision |
| EFV400+TDF+FTC vs RPV+TDF+FTC | Indirect | Moderate | Imprecision |  | Indirect | Moderate | Imprecision |
| EFV400+TDF+FTC vs EVG/c+TDF+FTC | Indirect | Moderate | Imprecision |  | Indirect | Moderate | Imprecision |
| EFV400+TDF+FTC vs DTG+TDF+FTC | ‒ | ‒ | ‒ |  | Indirect | Low | Study Limitations; Imprecision |
| EFV400+TDF+FTC vs DTG+ABC+3TC | Indirect | Moderate | Imprecision |  | Indirect | Moderate | Imprecision |
| EFV400+TDF+FTC vs DTG+TAF+FTC | Indirect | Moderate | Imprecision |  | Indirect | Moderate | Imprecision |
| EFV400+TDF+FTC vs BIC+TAF+FTC | Indirect | Moderate | Imprecision |  | Indirect | Moderate | Imprecision |
| EFV+ABC+3TC vs RPV+TDF+FTC | Indirect | Moderate | Study Limitations |  | Indirect | Low | Study Limitations; Imprecision |
| EFV+ABC+3TC vs EVG/c+TDF+FTC | Indirect | Low | Study Limitations; Imprecision |  | Indirect | Low | Study Limitations; Imprecision |
| EFV+ABC+3TC vs DTG+TDF+FTC | ‒ | ‒ | ‒ |  | Indirect | Low | Study Limitations; Imprecision |
| EFV+ABC+3TC vs DTG+ABC+3TC | Indirect | Moderate | Study Limitations |  | Indirect | Low | Study Limitations; Imprecision |
| EFV+ABC+3TC vs DTG+TAF+FTC | Indirect | High | ‒ |  | Indirect | Low | Study Limitations; Imprecision |
| EFV+ABC+3TC vs BIC+TAF+FTC | Indirect | Moderate | Study Limitations |  | Indirect | Low | Study Limitations; Imprecision |
| RPV+TDF+FTC vs EVG/c+TDF+FTC | Indirect | Moderate | Imprecision |  | Indirect | Moderate | Imprecision |
| RPV+TDF+FTC vs DTG+TDF+FTC | ‒ | ‒ | ‒ |  | Indirect | Low | Study Limitations; Imprecision |
| RPV+TDF+FTC vs DTG+ABC+3TC | Indirect | Moderate | Imprecision |  | Indirect | Moderate | Imprecision |
| RPV+TDF+FTC vs DTG+TAF+FTC | Indirect | Moderate | Imprecision |  | Indirect | Moderate | Imprecision |
| RPV+TDF+FTC vs BIC+TAF+FTC | Indirect | Moderate | Imprecision |  | Indirect | Moderate | Imprecision |
| EVG/c+TDF+FTC vs DTG+TDF+FTC | ‒ | ‒ | ‒ |  | Indirect | Low | Study Limitations; Imprecision |
| EVG/c+TDF+FTC vs DTG+ABC+3TC | Indirect | Moderate | Imprecision |  | Indirect | Moderate | Imprecision |
| EVG/c+TDF+FTC vs DTG+TAF+FTC | Indirect | Moderate | Imprecision |  | Indirect | Moderate | Imprecision |
| EVG/c+TDF+FTC vs BIC+TAF+FTC | Indirect | Moderate | Imprecision |  | Indirect | Moderate | Imprecision |
| DTG+TDF+FTC vs DTG+ABC+3TC | ‒ | ‒ | ‒ |  | Mixed | Low | Study Limitations; Imprecision |
| DTG+TDF+FTC vs DTG+TAF+FTC | ‒ | ‒ | ‒ |  | Indirect | Low | Study Limitations; Imprecision |
| DTG+TDF+FTC vs BIC+TAF+FTC | ‒ | ‒ | ‒ |  | Indirect | Low | Study Limitations; Imprecision |
| DTG+ABC+3TC vs DTG+TAF+FTC | Indirect | Moderate | Imprecision |  | Indirect | Moderate | Imprecision |
| DTG+ABC+3TC vs BIC+TAF+FTC | Mixed | Moderate | Imprecision |  | Mixed | Moderate | Imprecision |
| DTG+TAF+FTC vs BIC+TAF+FTC | Mixed | Moderate | Imprecision |  | Mixed | Moderate | Imprecision |
| Ranking of treatments | ‒ | High | ‒ |  | ‒ | Moderate | Imprecision |

ABC, abacavir; BIC, bictegravir; DTG, dolutegravir; EFV, efavirenz; EFV400, 400mg efavirenz; EVG/c, cobicistat-boosted elvitegravir; FTC, emtricitabine; RAL, raltegravir; RPV, rilpivirine; TAF, tenofovir alafenamide; TDF, tenofovir disoproxil fumarate; vs, versus; 3TC, lamivudine.
